# Two Genomic Regions Contribute Disproportionately to Geographic Differentiation in Wild Barley

**DOI:** 10.1534/g3.114.010561

**Published:** 2014-04-22

**Authors:** Zhou Fang, Ana M. Gonzales, Michael T. Clegg, Kevin P. Smith, Gary J. Muehlbauer, Brian J. Steffenson, Peter L. Morrell

**Affiliations:** * Department of Agronomy and Plant Genetics, University of Minnesota, St. Paul, Minnesota 55108; † Department of Plant Pathology, University of Minnesota, St. Paul, Minnesota 55108; ‡ Department of Plant Biology, University of Minnesota, St. Paul, Minnesota 55108; § Department of Ecology and Evolutionary Biology, University of California, Irvine, California 92697

**Keywords:** environmental association, local adaptation, population structure, chromosome structural variation, wild barley

## Abstract

Genetic differentiation in natural populations is driven by geographic distance and by ecological or physical features within and between natural habitats that reduce migration. The primary population structure in wild barley differentiates populations east and west of the Zagros Mountains. Genetic differentiation between eastern and western populations is uneven across the genome and is greatest on linkage groups 2H and 5H. Genetic markers in these two regions demonstrate the largest difference in frequency between the primary populations and have the highest informativeness for assignment to each population. Previous cytological and genetic studies suggest there are chromosomal structural rearrangements (inversions or translocations) in these genomic regions. Environmental association analyses identified an association with both temperature and precipitation variables on 2H and with precipitation variables on 5H.

The wild progenitors of major crops have long been recognized as a valuable genetic resource (Harris 1990). Natural populations of crop wild relatives have the potential to serve as a source of alleles that contribute to valuable agronomic traits, including cold or drought tolerance (Volis *et al.* 2002a, 2004), improved disease resistance (Fetch *et al.* 2003), and yield increase (Cohen and Galinat 1984). Plant germplasm repositories have made substantial investments in the preservation of accessions of crops and their wild relatives (Schoen and Brown 2001). Modern genetic approaches have increased the value of these resources as quantitative trait locus (QTL) mapping, association studies, and molecular population genetic studies have combined to uncover specific alleles associated with traits of potential value for crop improvement (Ross-Ibarra *et al.* 2007; Takeda and Matsuoka 2008).

Wild barley presents an especially valuable source of potentially useful genes because of its broad geographic distribution and ecological adaptation, spanning ∼3500 km east to west from the Levant (the eastern Mediterranean) and Anatolia (present day Turkey) to Central Asia, occurring across much of Southwestern Asia. Volis *et al.* (2002c) identified four wild barley ecotypes that exhibit differences in their level and patterns of phenotypic plasticity. In common garden studies, water stress caused a greater plastic response in the desert ecotype than in the Mediterranean ecotype, whereas for nutrient stress, plasticity was higher in the Mediterranean ecotype than in the desert ecotype (Volis *et al.* 2002b). The observed differences in ecotype plasticity or local variation in phenotypic traits suggest environmentally induced local adaptation (Volis *et al.* 2000, 2002c). Moreover, alleles from wild barley (*Hordeum vulgare* ssp. *spontaneum*) have been used in barley breeding programs for cultivated barley improvement (Von Korff *et al.* 2005, 2008; Schmalenbach *et al.* 2009). QTL analyses in an advanced backcross double haploid population derived from a cross between a barley cultivar and a wild barley accession demonstrates that wild barley harbors valuable alleles that can improve yield (Von Korff *et al.* 2006; Schmalenbach *et al.* 2009) and malting quality traits (Von Korff *et al.* 2008) and strongly reduce disease symptoms (Von Korff *et al.* 2005). QTL analysis of a recombinant inbred line population and an advanced backcross population derived from crosses between a barley cultivar and a wild barley accession reveals that the wild barley accession contains alleles that confer resistance to multiple fungal pathogens (Yun *et al.* 2005, 2006). Recently, association and candidate gene resequencing studies have begun to uncover evidence that alleles contributing to agronomically important phenotypes, such as flowering time, may have been introduced from geographic regions outside the initial region of barley domestication, with locally adaptive variants contributing to successful cultivation at higher latitudes (Jones *et al.* 2008).

Examination of population structure in crop wild progenitors can result in a better understanding of the number and geographic region of domestication events (Morrell and Clegg 2007), which is fundamental to understanding the processes that drove human domestication of plants. The potential to fully exploit genetic variation in the wild relatives of a crop depends, in part, on determining which portions of the range of wild progenitors have and have not contributed to diversity in the domesticates (Zohary and Hopf 2000).

A number of previous studies have examined wild barley genetic diversity (Nevo *et al.* 1986; Morrell and Clegg 2007; Jakob *et al.* 2014). Analysis of 27 allozyme loci in 2125 individuals sampled from Israel, Turkey, and Iran suggested geographic differentiation in wild barley populations (Nevo *et al.* 1986) but did not sample the complete geographic range of wild barley. Genetic assignment analysis based on resequencing of 18 loci in a sample of 25 to 45 wild barley individuals identified a primary geographic partition of samples into regions east and west of the Zagros Mountains (Morrell and Clegg 2007). The assignment analysis was limited by sample size but suggested that other geographically distinct wild barley subpopulations might be identified in a larger sample (Morrell and Clegg 2007; Saisho and Purugganan 2007).

The natural range of wild barley includes a variety of environmental conditions, from arid regions in Central Asia and the Syrian desert to coastal regions along the Mediterranean with relatively high rainfall, and from the cold environments in the Zagros Mountains, the western reaches of the Himalayas, and the Iranian plateau to relatively warm lowland Mediterranean coastal regions. By associating the geographic distribution of sequence polymorphisms in georeferenced samples with environmental variables, genomic regions and individual polymorphisms correlated with differences in drought or cold tolerance or other environmental factors may be identified for further investigation.

The questions we seek to explore in this article are what ecological factors are most associated with observed population structure, how does the geographic distribution of populations relate to allele frequency differentiation genome-wide, and how can we use this information to improve conservation and utilization of wild barley genetic diversity? We report the examination of geographic structure and genetic differentiation using 3072 SNPs (Close *et al.* 2009) genotyped in a sample of 318 wild barley accessions. We detect two primary populations of wild barley separated by the Zagros Mountains and three subpopulations within each of the two primary populations. Comparison of the two primary populations reveals two pericentromeric regions on the long arms of 2H and 5H that are associated with a substantial fraction of the geographic differentiation in allele frequencies observed in wild barley. The genetic variation in these genomic regions suggests cryptic chromosomal structural rearrangements. Environmental association analyses reveal strong association between these genomic regions and precipitation or temperature.

## Materials and Methods

### Plant materials

The 318 sampled wild barley accessions are known as the Wild Barley Diversity Collection (WBDC) (Steffenson *et al.* 2007). WBDC accessions were selected to be representative of the geographic range of wild barley, accounting for multiple ecogeographic features (*e.g.*, latitude, elevation, temperature range, and rainfall). The majority of accessions (77.4%) are from the Fertile Crescent, with the balance from Central Asia (15.7%), North Africa (3.8%), and the Caucasus region (2.8%). Individual accessions were self-fertilized for three generations to create inbred lines. Accession numbers, latitude, and longitude information for each sample are in Supporting Information, Table S1.

### Genotypic data

The WBDC accessions were genotyped using the Illumina Golden Gate Genotyping Assay with two Barley Oligo Pool Assay (BOPA) chips (BOPA1 and BOPA2), each including 1536 SNPs (Close *et al.* 2009). The SNPs were discovered by comparison of DNA sequence from expressed sequence tags and sequenced PCR amplicons, derived principally from one wild barley accession and eight malting barley cultivars, primarily from Europe and the United States (Close *et al.* 2009).

The program ALCHEMY (Wright *et al.* 2010) was used to generate machine-scored, automated genotype calls. The program incorporates estimated inbreeding coefficients for each sample to improve accuracy of genotype estimation. Unlike programs such as GenomeStudio, ALCHEMY does not assume Hardy-Weinberg Equilibrium (HWE) genotypic frequencies at each SNP. ALCHEMY is based on a Bayesian model of the raw intensity data and can accurately call genotypes. We used three approaches to verify the accuracy of genotype calls. First, WBDC355 (OUH602) has been genotyped separately, with variants segregating in a mapping population (OUH602 by the cultivar Haruna Nijo) (Sato *et al.* 2009; Muñoz-Amatriaín *et al.* 2011). Second, genotypes from two lines (WBDC218 and WBDC228) were estimated from RNA-Seq (see below for details of RNA-Seq data processing), so these data were used for validation. Third, all SNPs on BOPA1 were also called manually in GenomeStudio. Only 5% of SNPs have posterior probability of genotype calls <0.95 from ALCHEMY. We considered these SNPs as missing data.

Before running ALCHEMY, SNPs in BOPA1 and BOPA2 with strong compression or multiple clusters were removed. Subsequent to initial SNP calling, the following quality control steps were applied to the genotyping data. First, we eliminated SNPs that were monomorphic in the wild barley sample. Second, we removed all SNPs that included ≥15% missing data based on the rationale that large amounts of missing data at a SNP could be associated with inaccurate genotypes. Finally, observed heterozygosity was used as an additional quality control measure. Wild barley is a highly self-fertilizing species (Brown *et al.* 1978) and the WBDC accessions have been subject to three rounds of inbreeding. SNPs with observed heterozygosity >10% were removed on the rationale that the genotypes were likely in error. SNPs with centromeric genetic map positions were identified based on the consensus genetic map of Muñoz-Amatriaín *et al.* (2011). In the inbred WBDC lines, observed heterozygosity was extremely low (0.2%), thus we treat the data as haploid genotypes.

SNPs were annotated using the program SNPMeta (Kono *et al.* 2014). Annotation for each SNP included GenBank ID, gene short name, whether the SNP occurs in coding or noncoding sequence, the SNP position within a codon, and determination of whether the SNP is silent or induces an amino acid replacement. Among the 3072 BOPA SNPs, 2508 were annotated, with 338 annotations derived from named genes.

Along with SNP data, we examined 29 microsatellite loci in all WBDC accessions (Ramsay *et al.* 2000; Li *et al.* 2003; Varshney *et al.* 2007). Microsatellites offer the advantage of reduced ascertainment bias, because the microsatellites are selected to be polymorphic, but the selection process is not conditional on the presence of an individual polymorphism (Haasl and Payseur 2010). Microsatellite locus names, repeat number, repeat unit length, total size, and heterozygosity for each microsatellite can be found in Table S2. This information was obtained from GrainGenes: A Database for Triticeae and *Avena* (Matthews *et al.* 2003). The program *R*_ST_ Calc (Goodman 1997) was used to compare allele frequency differences among partitions of the sample, assuming a stepwise mutation model (Slatkin 1995).

### RNA-Seq data processing

RNA-Seq reads from *Hordeum bulbosum* accession Cb2920/4 (used as outgroup to infer ancestral state) and wild barley WBDC218 and WBDC228 were trimmed of adapter contamination with Scythe (https://github.com/vsbuffalo/scythe) and then aligned to the Morex draft sequence (Mayer *et al.* 2012) with Bowtie 2 (Langmead and Salzberg 2012). We adjusted read mapping parameters to accommodate the expected divergence between *H. vulgare* lines (∼1%) and between *H. vulgare* and *H. bulbosum* (∼3%) (Morrell *et al.* 2014). Alignments were processed with Samtools (Li *et al.* 2009) and the Genome Analysis Toolkit (GATK) (McKenna *et al.* 2010; DePristo *et al.* 2011) according to GATK best practices (http://www.broadinstitute.org/gatk/guide/best-practices). For realignment around indels, we used a set of high-confidence indels reported in Sanger resequencing datasets (Caldwell *et al.* 2006; Morrell *et al.* 2006; Morrell and Clegg 2007; Morrell *et al.* 2014). We then extracted the base calls at each BOPA SNP location using tools from the GATK. For all SNPs where it can be inferred, the ancestral state is listed in Table S3.

### Geographic differentiation

We examined the geographic structure within the range of wild barley with Bayesian genetic assignment implemented in the program STRUCTURE (Pritchard *et al.* 2000; Falush *et al.* 2003). We treated individual samples as haploid and explored both admixture and no admixture models and models with correlated and uncorrelated allele frequencies with *K* = 2 − 10 clusters. Because a model with no admixture and uncorrelated allele frequencies resulted in higher likelihoods, it was used for the final analyses. For each value of *K*, we used 10 replicate runs with a burn-in length of 100,000 iterations and a run length of 100,000 iterations.

Haplotypes were defined based on five adjacent SNPs and used both for comparison of haplotype diversity and as input for genetic assignment analysis. Combining markers into haplotypes results in multi-allelic data that can improve inference of population structure (Haasl and Payseur 2010; Gattepaille and Jakobsson 2012). To infer missing data, we used the program fastPHASE (Scheet and Stephens 2006) with 20 random starts and 25 iterations of the Expectation-Maximization algorithm. The number of SNPs within each haplotype was determined based on optimal numbers from the simulation study of Gattepaille and Jakobsson (2012) and the total number of SNPs in our samples. To deal with label switching (where cluster names change between replicate runs) and with true multimodality (where individual samples switch clusters in replicate runs), we used CLUMPP (Jakobsson and Rosenberg 2007) to summarize assignment results across replicate runs. The program Infocalc was used to calculate the informativeness for assignment (*In*) (Rosenberg *et al.* 2003) for each haplotype using the clusters identified by STRUCTURE. Informativeness for assignment identifies the information content for genetic assignment for markers based on the degree to which each locus (or haplotype) contributes to discrimination among populations (Rosenberg *et al.* 2003).

The prcomp function in R (R Development Core Team 2012) was used to perform principal component analysis (PCA). Each WBDC accession was assigned to clusters based on significant principal components (PCs) using the Ward clustering method in the R hclust function. To compare the similarity between genetic variation on a PCA plot and geographic maps of sample locations, we used Procrustes analysis to find a rotation that maximizes the similarity (Wang *et al.* 2012).

Within and among inferred clusters, we estimated hierarchical F-statistics (Yang 1998) using the Hierfstat package (Goudet 2005) in R. We calculated summary statistics, including number of segregating sites, and number of private alleles in each cluster using tools from the libsequence library (Thornton 2003) and estimated average pairwise SNP diversity within each cluster using the R package ape (Paradis *et al.* 2004). Rarefaction was used to analyze allelic diversity across populations while correcting for sample size differences using the program ADZE (Szpiech *et al.* 2008).

Linkage disequilibrium (LD) as measured by *r*^2^ (Hill and Robertson 1968) was calculated for all possible pairwise comparisons on each linkage group based on SNPs with minor allele frequency (MAF) >5%. The LDheatmap package (Shin *et al.* 2006) was used to generate plots of LD relative to genetic distance.

### Environmental–genetic correlations

To identify genomic segments potentially contributing to local adaptation, we divided samples into the two primary clusters (the Eastern and Western populations) and six clusters identified by genetic assignment in STRUCTURE and calculated *F*_ST_ (Weir and Cockerham 1984). The assumption is that SNPs linked to genomic regions contributing to local adaptation will show greater allele frequency divergence (higher *F*_ST_) than those affected only by demography (Cavalli-Sforza 1966; Lewontin and Krakauer 1973).

Environmental variables, including altitude, monthly precipitation, monthly maximum and minimum temperatures, and 19 additional bioclimatic variables were downloaded from www.worldclim.org (Hijmans *et al.* 2005). DIVA-GIS (Hijmans *et al.* 2001) was used to extract climate data at 5 arc-min (∼10 km) resolution for each sample. We focused on measurements within the growing season for wild barley, from late autumn to spring, so we removed environmental variables related to summer temperature and precipitation. The environmental variables used are listed in Table S4A. Environmental variables were scaled to a mean of 0 and SD of 1 and summarized into principal components. The significant PCs of environmental variables were used for Bayenv (Coop *et al.* 2010).

We used Bayenv to identify the correlation between SNPs and environmental variables. Bayenv requires population information to account for population structure, making use of allele frequency within a set of samples representing a localized environment to correct for population structure in environmental association. We used PCA to group all samples into 17 clusters with a mean sample size of 17 accessions. The number of optimal stratifications in our data was determined using Velicer’s minimum average partial test (Shriner 2011). We used all SNPs to construct the covariance matrix. Two independent runs of 30,000 iterations were compared to control for convergence and the final covariance matrix is the mean of these two independent runs. Bayenv was used to estimate the Bayes factor for each SNP with each environmental variable using 50,000 iterations. SNPs were considered candidates contributing to local adaptation if they had an average Bayes factor above the 95^th^ percentile genome-wide for five separate runs.

Because the wild barley samples cover a large continuous geographic range, spatial ancestry analysis (SPA) (Yang *et al.* 2012) was used to model allele frequency change for each SNP as a function of the location of the individual in geographic space. SNPs contributing to local adaptation potentially have larger gradients in allele frequency, reflected in a high SPA score (Yang *et al.* 2012). SNPs were considered candidates for local adaptation if their SPA score was above the 95^th^ percentile genome-wide.

SNPs that are outliers (above the 95^th^ percentile genome-wide) in the *F*_ST_, environmental association, or SPA analyses can be considered candidate variants either causative or, more likely, linked to loci involved in local adaptation. Enrichment analysis of candidates among genic *vs.* nongenic and nonsynonymous *vs.* synonymous was performed by resampling the number of SNPs in each candidate list randomly from the genome 1000 times. Enrichment analyses included sets of SNPs genome-wide, centromeric regions, and the two regions that are putative structural rearrangements (inversions or translocations). This generated a distribution of ratios of the number of genic to the number of nongenic SNPs and ratios of the number of nonsynonymous to the number of synonymous SNPs in each analysis, which can be compared to the observed ratios from the data.

## Results

Initial screening of relatedness among accessions identified 30 wild barley accessions either with a large genetic distance from the majority of accessions or that appear to be duplicated within the sample. The large genetic distance was also associated with a high degree of identity to barley landraces genotyped on the same platform. The increase in genetic distance appears to result from ascertainment bias due to the discovery of barley SNPs primarily among cultivated samples, which results in a larger number of segregating polymorphisms in barley landrace accessions than in wild barley (Russell *et al.* 2011). The 30 accessions were excluded from further analysis because they constitute duplicate accessions or because genotypic composition suggests they either could be feral barley accessions or were subject to recent introgression. Four accessions do not have known latitude and longitude of origin, so they were removed from analyses; thus, 284 wild barley accessions from the WBDC were used in this study (Table S1). After all quality control measures, 2330 SNPs were assayed in each accession (Table S5).

Microsatellite loci were extremely polymorphic among wild barley accessions with an average of 20 alleles per locus. Allele size for individual microsatellites is normally distributed and thus accords with a stepwise mutation model (Ohta and Kimura 1973; Valdes *et al.* 1993).

### Population structure

A Mantel test identifies a positive correlation between geographic distance and genetic distance (Mantel statistic: 0.37; significance at 0.001), consistent with isolation by distance among these wild barley accessions.

**Figure 1:**
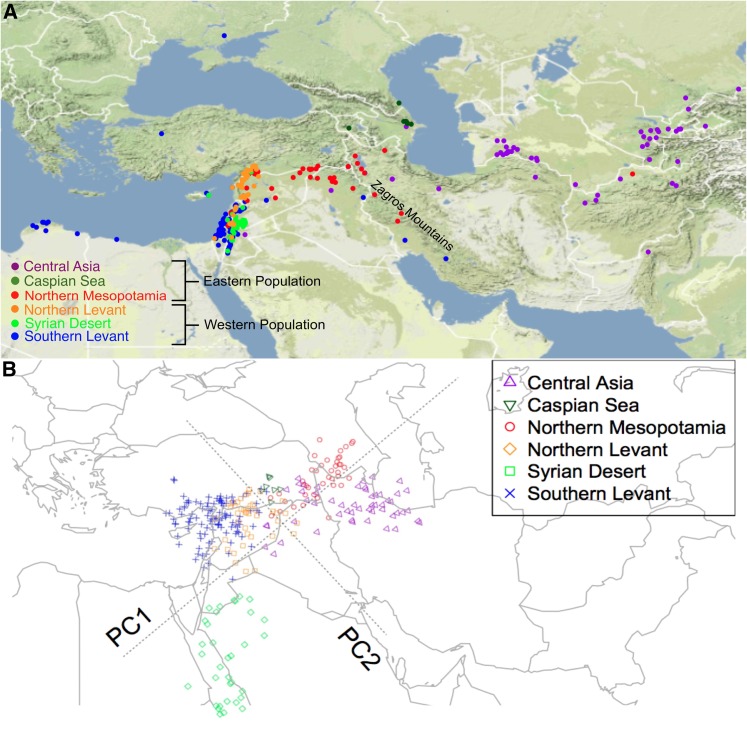
(A) Population structure in wild barley. Each of the six colors represents one of six subpopulations. There are three subpopulations nested within both the Eastern and Western populations. (B) Procrustes-transformed PCA plot of genetic variation in wild barley.

STRUCTURE results based on single-SNP and 5-SNP haplotypes for *K* = 2 identified populations east and west of the Zagros Mountains. Samples from a broad portion of the range, including Central Asia (particularly east of the Caspian Sea), the Iranian Plateau, and most samples in Northern Mesopotamia (modern Northern Iraq and Syria) form an Eastern population, and samples west of the Zagros Mountains, including samples from the Levant, and around the Mediterranean to North Africa form a Western population (Figure 1A). There is also isolation by distance within the Eastern population (Mantel statistic: 0.31; significance at 0.001) and the Western population (Mantel statistic: 0.20; significance at 0.001). For genetic assignment based on individual SNPs, there are six accessions from Central Asia assigned to the Western population (data not shown). Thus, genetic assignment from haplotype data shows greater consistency with geographic location of origin. The broad-scale geographic patterns identified from SNP and 5-SNP haplotypes are not readily reflected in genetic assignment based on the 29 microsatellite loci (data not shown). The individual alleles are relatively rare and have low informativeness for assignment, an issue attributable to the high levels of polymorphism in these microsatellites (Table S2).

For STRUCTURE analysis based on haplotype data, when *K* = 3, a group of accessions from the Syrian desert region becomes an independent cluster from the Western population. As *K* increases to 4, the samples along the east coast of the Mediterranean split into two groups, one in the north (Northern Levant) and the other in the south (Southern Levant). The Eastern population begins to differentiate as *K* is increased to 5. The samples in Central Asia are separated from samples from Northern Mesopotamia and those from west of the Caspian Sea. With *K* = 6, the seven samples from the Caspian Sea become an independent cluster (Figure 1A). For the present sample, *K* = 6 provides a clear distinction among populations and the genetic assignment is not constrained by very small sample size within individual clusters. The average informativeness for assignment for individual SNPs is 0.03 for *K* = 2 and 0.10 for *K* = 6, increasing to 0.14 for *K* = 2 and 0.47 for *K* = 6 when based on haplotypes (Figure S1).

Among the six clusters, three are within the Eastern population (Central Asia, Caspian Sea, and Northern Mesopotamia) and three are in the Western population (Northern Levant, Syrian Desert, and Southern Levant), constituting hierarchical population structure nested within the major Eastern and Western populations. The comparative *F*_ST_ analyses identify both a significant effect of individuals within subpopulations (p-value = 0.01; 100 permutations; observed likelihood ratio statistics = 94,259.55) and a significant effect of subpopulations within populations (p-value = 0.04; 100 permutations; observed likelihood ratio statistics = 33,632.57). Population structure is best explained by a six-subpopulation model (variance components = 17.7%); in contrast, the two-population model provides a poorer fit to the data (variance components = 10.6%). *F*_ST_ values are also higher at the subpopulation level than the population level (Table 1). Within the two higher-level populations, 20.5% of the genetic differentiation can be explained by the three subpopulations within the Eastern population, whereas 13.2% can be explained by the three subpopulations within the Western population.

**Table 1:** Hierarchical F-statistics comparing different levels of the hierarchical population structure

	Population	Subpopulation
Total	0.042	0.191
Population	0.000	0.155

The values reported include the F statistics for two primary populations *vs.* total, six subpopulations *vs.* two primary populations, and six subpopulations *vs.* total.

In the PCA examination of population structure, the first PC separates all samples into the Eastern and Western populations. When adding the second PC, all six subpopulations are clearly differentiated. A PCA plot of genetic variation closely reflects geography in the population after a 42.44° counterclockwise rotation of the PCA plot using Procrustes analysis. The boundary of the Eastern and Western population approximately parallels the Zagros Mountains (Figure 1B). The Syrian Desert subpopulation is noteworthy in showing greater PC distance within and among samples, possibly because of greater genetic drift within this subpopulation. A mean pairwise *F*_ST_ analysis indicates a higher mean *F*_ST_ value for this subpopulation (0.09) than for the other two Western subpopulations (0.05 and 0.04).

### Population differentiation

The average genome-wide *F*_ST_ between the Eastern and Western populations is 0.07. The boundary of these two populations is close to the Zagros Mountains (Figure 1A), which forms a potential barrier to migration (Lin *et al.* 2001; Morrell *et al.* 2003). The most northerly portion of the Zagros Range is at ∼48° E longitude, trending from the northwest to the southeast, so we also compared the allele frequency differentiation between the two populations east and west of 48° E. For this geographic contrast, the average *F*_ST_ genome-wide is 0.06 and the correlation coefficient (*r*) between this partition based on the Zagros Mountains and the previous partition based on genetic assignment analysis is 0.473, which supports the hypothesis that the Zagros Mountains act as a natural barrier that bisects wild barley into the Eastern and Western populations. For the 29 microsatellite loci, the average *R*_ST_ between the Eastern and Western populations is 0.15.

**Figure 2:**
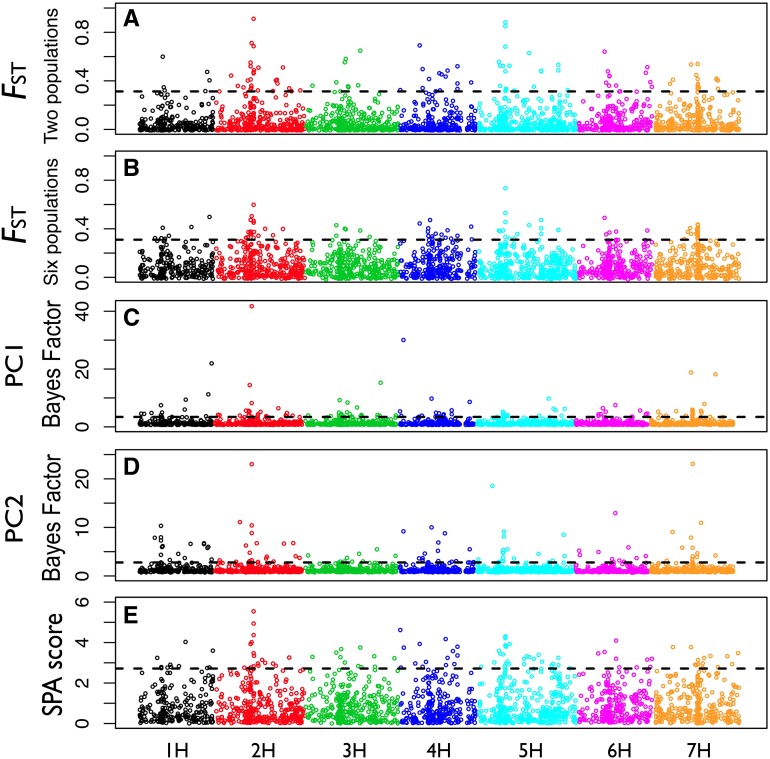
(A) *F*_ST_ between the Eastern and Western wild barley populations. (B) Pairwise *F*_ST_ based on all six subpopulations. The dotted line is the 95^th^ percentile of *F*_ST_ genome-wide. (C) Bayes factors for correlation between allele frequencies and PC1. (D) Bayes factors for correlation between allele frequencies and PC2. (E) SPA score genome-wide from spatial analysis. The 95^th^ percentile of the distribution of Bayes factors or SPA scores is indicated by a horizontal dashed line.

SNPs with high *F*_ST_ values between the Eastern and Western populations (Figure 2A) or among all the six subpopulations (Figure 2B) are most abundant in two genomic regions, one on linkage group 2H, from ∼67 to 74 cM, and the other on 5H, from ∼47 to 52 cM. For the 2H and 5H regions, the mean *F*_ST_s are 0.20 (56 SNPs) and 0.17 (32 SNPs), respectively, *vs.* a genome-wide *F*_ST_ = 0.07.

Many SNPs or haplotypes genome-wide with high informativeness for assignment are in these two high *F*_ST_ regions (Figure S1). Among all SNPs above the 95^th^ and 99^th^ percentiles (*In* = 0.31 and 0.42), 33% and 52% are in these two regions (Figure S1). However, when we perform genetic assignment analysis after masking these two high *F*_ST_ regions, the probability of assignment for each wild barley accession into the Eastern and Western population is nearly identical for all but one accession. This result reflects the relatively high informativeness for assignment observed for SNPs within pericentromeric regions on all linkage groups (Figure S1). Therefore, population structure in these two high *F*_ST_ regions is similar to the genome-wide pattern and the genome-wide pattern is driven by a high degree of differentiation in pericentromeric regions.

The joint unfolded site frequency spectrum demonstrates that there are more rare variants in the Western population than in the Eastern population (Figure S2). Percent pairwise differences are lower in the Eastern population than in the Western population (Table 2). There are more private SNPs in the Western population than in the Eastern population (430 *vs.* 86) (Table 2). Because the sample size is different between the Eastern and Western populations, we used rarefaction to correct for sample size. Despite the correction, both the mean number of distinct alleles per locus (Figure S3A) and the mean number of private alleles per locus (Figure S3B) are higher in the Western than the Eastern population. The Southern Levant subpopulation has the highest values for the number of segregating sites and number of private SNPs, whereas the Caspian Sea subpopulation has the lowest values for these summary statistics (Table 2).

**Table 2:** Diversity summary statistics for the two populations and six subpopulations

Population	Size	# Segregating Sites	# Private SNPs	Percent Pairwise Difference (SD)	Microsatellite Expected Heterozygosity
**Eastern**	101	2196	86	0.20 (0.04)	0.740
Caspian Sea	7	1146	2	0.10 (0.02)	0.672
Central Asia	53	2027	22	0.19 (0.04)	0.734
Northern Mesopotamia	41	1975	13	0.17 (0.03)	0.717
**Western**	183	2285	430	0.23 (0.03)	0.742
Northern Levant	42	2033	19	0.21 (0.02)	0.740
Southern Levant	107	2197	49	0.22 (0.02)	0.736
Syrian Desert	34	1916	6	0.16 (0.03)	0.722

Data include the sample size, number of segregating sites, number of private SNPs, percent pairwise difference with SD, and microsatellite expected heterozygosity .

### Structural rearrangements

**Figure 3:**
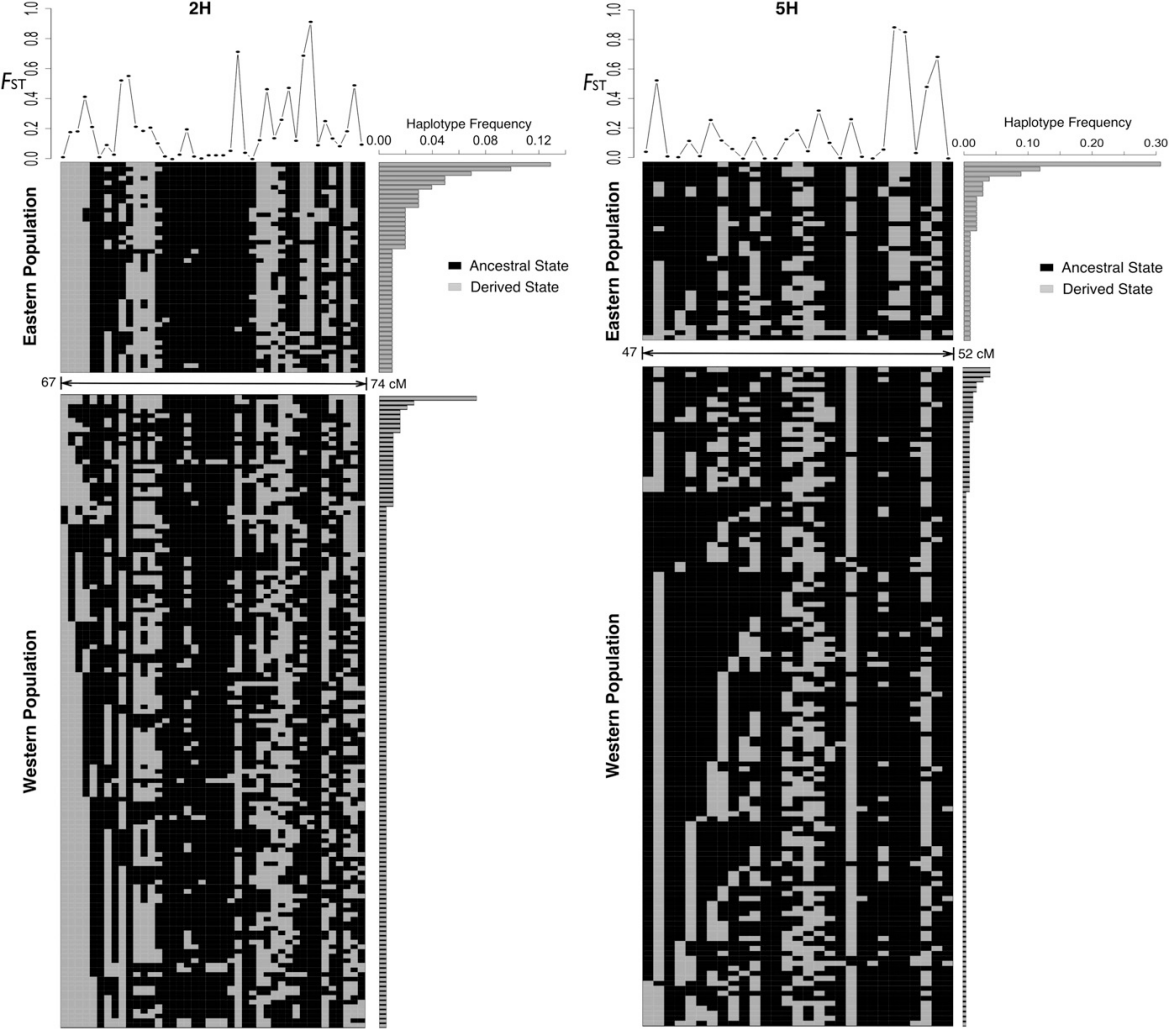
Diagram of haplotype diversity in the two putative chromosome structural rearrangements on 2H and 5H. Haplotypes are divided into the two primary populations identified by genetic assignment. Each SNP is represented by either the ancestral state (black) or the derived state (gray). The frequencies of each of the haplotypes from the Eastern population (top) and from the Western population (bottom) are shown on the right. *F*_ST_s for each SNP between these two populations are shown at the top of the haplotype diagram.

The high *F*_ST_ regions on 2H and 5H are potentially attributable to chromosomal structural variants. Population genetic variation, particularly patterns of LD, can suggest structural variation (Huynh *et al.* 2011; Long *et al.* 2013). The average pairwise LD in the high *F*_ST_ region on 2H (*r*^2^ = 0.085) is higher than other regions of 2H (*r*^2^ = 0.018) and the adjacent regions with similar size left and right of the high *F*_ST_ region, where *r*^2^ = 0.021 for both regions. The average pairwise LD in the high *F*_ST_ region on 5H (*r*^2^ = 0.097) is also higher than other regions on 5H (*r*^2^ = 0.019) and the adjacent regions with similar size to the left and right of the high *F*_ST_ region (*r*^2^ = 0.017 and 0.020). The 5-SNP segment with the lowest haplotype number (4) on 2H is within the high *F*_ST_ region (Figure S4). As on other linkage groups, the segment with the lowest haplotype number on 2H and 5H is within the centromeric region (Figure S4). The centromeric region on 2H overlaps with the high *F*_ST_ region, whereas the centromere is distal to the high *F*_ST_ region on 5H (Figure S4). The observed LD, *F*_ST_, and haplotype number patterns are consistent with recent positive selection and/or chromosome structural rearrangements. There are more unique haplotypes in the Western population than in the Eastern population (Figure 3). In the Eastern population, both of the two regions on 2H and 5H are dominated by a few haplotypes (Figure 3), which is potentially consistent with selection favoring these haplotypes.

### Evidence for local adaptation

SNPs that occur as outliers in the *F*_ST_ analysis may indicate genomic regions involved in local adaptation. Annotation information for SNPs that are above the 95^th^ percentile for *F*_ST_ between the Eastern and Western populations is listed in Table S6.

Environmental association analysis was used to identify genetic polymorphisms potentially involved in local adaptation. PCA reveals two major clusters of environmental variables (Figure S5). The first two PCs explain 80% of the total variance (Figure S5). The first PC includes most temperature variables, whereas most precipitation variables and altitude are in the second PC (Table S4B).

SNPs associated with both environmental PC1 and PC2 (above the 95^th^ percentile) are distributed on all linkage groups (Figure 2, C and D). The high *F*_ST_ region on 2H is highly associated with both PC1 and PC2 (Figure 2 C and D). The high *F*_ST_ region on 5H is also associated with PC2 (Figure 2D). The annotation information for SNPs that are above the 95^th^ percentile of association with PC1 and PC2 is listed in Table S7.

SPA analysis reveals that ∼20% of the SNPs in the high *F*_ST_ regions on both 2H (11 out of 56) and 5H (7 out of 32) show strong geographic gradients in allele frequencies as their SPA scores are above the 95^th^ percentile (2.72) (Figure 2E). The SNP with the highest SPA score (5.54) is in the high *F*_ST_ region on 2H. There are 24 SNPs with SPA score above the 99^th^ percentile (3.75), and nearly half of these SNPs (11) are in these two high *F*_ST_ regions on 2H and 5H. The annotation information for the SNPs with SPA scores above the 95^th^ percentile is in Table S8.

Enrichment analysis reveals that the two putative chromosomal structural rearrangements are enriched for genic SNPs in the outliers of PC2 from environmental analysis and SPA analysis (Figure S6A). There is no evidence of enrichment for nonsynonymous SNPs (Figure S6B).

## Discussion

### Hierarchical population structure

Wild barley shows strong hierarchical population structure, with primary structure east and west of the Zagros Mountains and three subpopulations identified in both the Eastern and Western populations (Figure 1A). Previous studies of sequence diversity in wild barley have identified population structure that strongly differentiates the Eastern and Western wild barley populations (Lin *et al.* 2001; Morrell and Clegg 2007; Saisho and Purugganan 2007). The present study samples a much larger number of accessions and includes only 22 SNPs in common with those sampled in previous studies. Despite the limited overlap of sampled SNPs, genetic assignment with *K* = 2 uncovers a similar geographic pattern. Moreover, the results of this study go beyond previous work by revealing a division into six subpopulations that explains 7.1% more variance compared with the primary, two-population division.

A nearly continuous geographic range represents a particular challenge for efforts to identify population structure and geographic discontinuities. However, populations of wild barley are much more common in the western portion of the range and below 1500 m (Zohary and Hopf 2000), thus the 3000-m to 4500-m peaks of the Zagros Mountains and the high elevation regions of the Iranian plateau are disruptions of an otherwise continuous range.

The Western population is more diverse than the Eastern population (Table 2), at least in part because the SNP discovery panel was composed primarily of Western cultivars. It should be noted that the discovery panel also included OUH602 (WBDC355), which assigns to the Central Asia (Eastern) wild barley population. Estimates of diversity based on resequencing a more limited set of wild barley samples indicate moderately higher levels of diversity in the Western than in the Eastern wild barley populations (Morrell and Clegg 2007; Morrell *et al.* 2014). The effect of ascertainment bias on the frequency spectrum depends on the population in which SNPs were discovered (Albrechtsen *et al.* 2010). Therefore, we observe more rare alleles in the Western population (Figure S2). There is a small but clear effect of ascertainment bias, which leads to an increased estimate of diversity in the Western population, because the Western population is more similar to the discovery panel. The size of a discovery panel is less important than the composition, as long as it is not extremely small (<4 chromosomes) (Albrechtsen *et al.* 2010). Based on coalescent simulations, a discovery panel of eight samples with a minimum allele count of three best-reflects the design parameters of the BOPA SNPs (Fang *et al.* 2013).

### Two putative chromosome rearrangements

Resequencing studies identified a large degree of heterogeneity in wild barley, in terms of both degree of population structure and levels of nucleotide sequence diversity (Lin *et al.* 2001; Morrell *et al.* 2003). Using simple coalescent simulations, Morrell *et al.* (2003) argued that stochastic variation alone was insufficient to explain intralocus heterogeneity and that selection, either through selective sweeps at some loci or through local adaptation at others, was necessary to explain observed patterns of diversity (Wright *et al.* 2005). Strong genetic differentiation between the Eastern and Western populations for the two regions on 2H and 5H suggest that some of the heterogeneity may result not from selection acting on individual loci, but rather on structural variants (Figure 2). Structural variants are quickly lost due to drift and purifying selection unless they confer a locally adaptive advantage. A structural variant that captures two or more alleles adapted to the local environment has a selective advantage that can cause it to spread (Kirkpatrick and Barton 2006).

The high *F*_ST_ region on 2H occurs in the same approximate chromosomal location as a chromosomal rearrangement identified in an eastern wild barley accession based on meiotic pairing studies (Konishi and Linde-Laursen 1988). Konishi and Linde-Laursen (1988) report a reciprocal translocation with 4H in a sample from Turkmenistan with the breakpoints of the translocation near the centromere on 2H. Using a three-point linkage test, Ramage and Suneson (1961) identify evidence of both an inversion and translocations on the long arm of 5H.

The two genomic regions with high *F*_ST_ also have above average levels of LD and low haplotype number (Figure S4). Environmental association analysis identifies multiple SNPs in these regions associated with both temperature and precipitation (Figure 2). SPA analysis identifies half of the SNPs in these two regions as outliers with dramatic change in allele frequency gradients, as identified by SPA scores above the 99^th^ percentile. The SPA method is particularly sensitive to SNPs that have steep geographic gradients in allele frequency and is scored based on individual accessions rather than populations (Yang *et al.* 2012).

The large number of candidate SNPs in these regions identified in multiple analyses indicate these two regions may harbor variants that are locally adaptive, with selection altering the frequency of nearby SNPs through genetic hitchhiking (Nielsen *et al.* 2005). Given the density of SNPs assayed in the present study and the relatively rapid decay of LD in wild barley (Morrell *et al.* 2005, 2006), the detection of multiple SNPs associated with environmental factors is likely to occur only in regions with suppressed recombination. These two high *F*_ST_ regions are 5 to 7 cM, potentially including hundreds of genes; thus, the patterns observed are likely due to chromosome structural variants that, through the inhibition of genetic exchange between chromosomal rearrangements, have the effect of slowing migration.

The Eastern accessions occur in a region that, on average, is more arid than the region occupied by the Western accessions, where most samples were collected from populations along the Mediterranean coast. The precipitation pattern is reflected in the environmental association analysis. Both of the two putative chromosome rearrangements on 2H and 5H are associated with precipitation variables (Figure 2).

Comparisons among genetic maps can provide confirmation of the presence of chromosomal rearrangements through the inference of alternative mapping order in populations where both mapping parents carry the alternative arrangement (Thomas *et al.* 2008; Lowry and Willis 2010) or through evidence of suppression of crossover when parents differ for the chromosomal arrangement (Fang *et al.* 2012). It is possible to identify mapping parents that differ for chromosomal rearrangements based on the presence of variants (SNPs) that are private (or nearly private) to the putative rearrangement (Fang *et al.* 2012) or in cases where the geographic distribution of the putative rearrangement is well-defined, using parents from geographic regions where the rearrangement is more common. Both putative structural rearrangements in wild barley occur in the eastern portion of the wild range. Barley is a cultigen with multiple origins (Morrell and Clegg 2007), with a greater contribution of eastern wild barley ancestry among Asian landraces (Morrell and Clegg 2007; Morrell *et al.* 2014). Barley doubled haploid genetic mapping populations compared by Muñoz-Amatriaín *et al.* (2011) include three populations that could potentially differ for the rearrangement: the Japanese malting barley cultivar Haruna Nijo by wild barley OUH602 (Sato *et al.* 2009); Haruna Nijo by food barley cultivar Akashinriki (Sato *et al.* 2011); and the Oregon Wolfe Barley (OWB) population (Costa *et al.* 2001; Stein *et al.* 2007; Szűcs *et al.* 2009).

Examining the genomic regions on 2H and 5H with highest allele frequency differentiation and levels of LD among wild barley accessions, we identify an average of 24 SNPs on 2H and 17 SNPs on 5H segregating among mapping parents in these genetic maps. The two Haruna Nijo maps include a single inferred crossover on both 2H and 5H, whereas the OWB maps include a larger number of crossovers on 2H and two crossovers on 5H. Crossover number in these genetic maps does not differ dramatically from that observed in the same genomic regions in mapping populations for Morex × Barke or Morex × Steptoe. Morex, Barke, and Steptoe are western cultivated barleys and are likely to bear the standard arrangement. Given the uncertainty of the presence of the putative rearrangements in mapping parents and limited marker density within the putative rearranged genomic regions, the present genetic maps neither convincingly refute nor support the presence of structural rearrangements.

We also noted that the two putative chromosome rearrangements are not differentiated by large numbers of private SNPs (Figure 3) as observed at the largest inversion (*Inv1n*) in teosinte, the wild progenitor of maize (Fang *et al.* 2012). This may be attributable in the discovery of SNPs primarily among western cultivars, which are unlikely to carry the rearrangement. Moreover, the putative translocation on 2H incorporates the centromere and the putative rearrangement on 5H is close to the centromeric region. Therefore, an alternative explanation for the patterns observed in these two genomic regions could be exceptional effects arising from suppressed recombination in the centromeric regions (Mayer *et al.* 2012).

### Useful wild barley alleles

Identification of functional variation that indicates local adaptation can contribute to sustained crop improvement. Crop wild progenitors have a history of adaptation to their local environments that is orders of magnitude older than the time since domestication, so wild populations have been exposed to natural selection for many more generations. Moreover, a domestication bottleneck decreases nucleotide diversity and causes the loss of valuable variants (Eyre-Walker *et al.* 1998). For these reasons, wild populations are expected to carry many novel nucleotide sequence variants and functional adaptations that are not present in domesticates.

We used several approaches to identify nucleotide polymorphisms potentially involved in local adaptation. SNPs that are outliers in the *F*_ST_, environmental association, and SPA analyses are potentially linked to loci contributing to local adaptation. The *F*_ST_ comparison assumes that genetic markers with extreme allele frequency differences among populations may contribute to local adaptation (Lewontin and Krakauer 1973), but this method has several limitations. First, the Lewontin and Krakauer approach suffers from a number of assumptions, including that all populations diverged at the same time (Nei and Maruyama 1975). Second, comparison of allele frequency differences with *F*_ST_ requires the prior identification of populations. Environmental association and SPA analyses complement and are consistent with the *F*_ST_ results. The results of both analyses support the conclusion that selection has contributed to the observed allele frequency differentiation likely driven by environmental factors or correlated selection pressures (Coop *et al.* 2010).

A number of SNPs within previously characterized barley loci are outliers in one or more of the analyses reported here. The SNP (12_30850) from the gene *Cbf4* is above the 95^th^ percentile of *F*_ST_ values for SNP frequencies compared between the Eastern and Western populations. *Cbf4* contributes to low-temperature tolerance in barley (Francia *et al.* 2004). From environmental association analysis, one SNP (11_11361) in *Cbf4* and another SNP (11_10989) in a cold-regulated gene *blt14* (Cattivelli and Bartels 1990; Grossi *et al.* 1998) are among outliers of PC1, which includes the temperature variables. SPA analysis reveals that SNP 12_30850 in *Cbf4* also shows a strong geographic gradient in allele frequency. These results establish the value of geographic analyses as a screen to identify potentially adaptive alleles.

### Summary

Two large pericentromeric regions on 2H and 5H make strong contributions to the population structure observed in wild barley. These two genomic regions are putative chromosome structural rearrangements that harbor variants that appear to contribute to environmental adaptation. In particular, the SNPs in these chromosomal regions are shown to be associated with temperature and precipitation variables. It will be important to determine how specific genetic variants within these rearrangements are associated with local adaptation. Nevertheless, the identification of genomic regions associated with environmental adaptation suggests an opportunity for crop improvement.

## Supplementary Material

Supporting Information
