# Supplementary material for: Two Genomic Regions Contribute Disproportionately to Geographic Differentiation in Wild Barley
Source: G3 (Bethesda). 2014 Apr 22;4(7):1193–203. doi: 10.1534/g3.114.010561 (PMC4455769; doi:10.1534/g3.114.010561)
Supplement: Supporting Information [file supp_4_7_1193__index.html]

Two Genomic Regions Contribute Disproportionately to Geographic Differentiation in Wild Barley — Supporting Information 

# Two Genomic Regions Contribute Disproportionately to Geographic Differentiation in Wild Barley

## Supporting Information for Fang *et al.*, 2014

**Files in this Data Supplement:**

- Supporting Information - Figures S1-S6 and Tables S1-S8 (PDF, 894 KB)
- Figure S1 - The informativeness for assignment for all SNPs (A, B) and five-SNP haplotypes (C, D) genome-wide based on (A, C) *K* = 2 and (B, D) *K* = 6. (PDF, 648 KB)
- Figure S2 - The joint unfolded site frequency spectrum based on all accessions from the Eastern population (upper triangle) and Western population (lower triangle). (PDF, 540 KB)
- Figure S3 - Rarefaction analysis comparing nucleotide diversity between the Eastern and Western populations, (A) mean number of distinct alleles per locus and (B) mean number of private alleles per locus. (PDF, 230 KB)
- Figure S4 - Population genetic analysis of the two high *F*ST regions. (PDF, 212 KB)
- Figure S5 - The proportion of variance explained by each PC of environmental variables. (PDF, 165 KB)
- Figure S6 - Enrichment analysis for (A) genic versus non-genic and (B) nonsynonymous versus synonymous SNPs. (PDF, 478 KB)
- Table S1 - 284 wild barley accessions used in this study, including their latitude and longitude information. (PDF, 139 KB)
- Table S2 - The name, repeat length, repeat unit length, total size and observed heterozygosity of the 29 microsatellites used in this study. (PDF, 134 KB)
- Table S4 - (A) Environmental variables and abbreviations used in this study; (B) Environmental variable and the corresponding loadings for the first two principal components. (PDF, 136 KB)
- Table S5 - BOPA SNPs used in this study, including their genetic positions. (PDF, 190 KB)
- Table S6 - SNPs with *F*ST based on the Eastern and Western populations above 95th percentile genome-wide, including genetic position, GenBank ID, gene short name, in non-coding or coding region (1st, 2nd or 3rd positions), and silent or replacement information. (PDF, 153 KB)
- Table S7 - SNPs with Bayes Factor from environmental association analysis (Bayenv) above 95th percentile genome-wide, including genetic position, GenBank ID, gene short name, in non-coding or coding region (1st, 2nd or 3rd positions), and silent or replacement information. (PDF, 146 KB)
- Table S8 - SNPs with SPA score above 95th percentile genome-wide, including genetic position, GenBank ID, gene short name, in non-coding or coding region (1st, 2nd or 3rd positions), and silent or replacement information. (PDF, 139 KB)
- Table S3 - Morex state and ancestral state (inferred using *H. Bulbosum* accession Cb2920/4) for all BOPA SNPs. (.txt, 30 KB)
