# Supplementary material for: Two Genomic Regions Contribute Disproportionately to Geographic Differentiation in Wild Barley
Source: G3 (Bethesda). 2014 Apr 22;4(7):1193–203. doi: 10.1534/g3.114.010561 (PMC4455769; doi:10.1534/g3.114.010561)
Supplement: Supporting Information [file supp_g3.114.010561_010561SI.pdf]

G3·Genes | Genomes | Genetics

Two genomic regions contribute disproportionately to geographic differentiation in wild barley

Zhou Fang,[*, §] Ana M. Gonzales,[*] Michael T. Clegg,[†] Kevin P. Smith,[*] Gary J. Muehlbauer,[*, §] Brian J. Steffenson,[‡] Peter L. Morrell[*,1]

[*] Department of Agronomy and Plant Genetics, University of Minnesota, St. Paul, MN 55108
[§] Department of Plant Biology, University of Minnesota, St. Paul, MN 55108
[†] Department of Ecology and Evolutionary Biology, University of California, Irvine, CA 92697
[‡] Department of Plant Pathology, University of Minnesota, St. Paul, MN 55108

[1]*Corresponding author*:
Peter L. Morrell
Department of Agronomy and Plant Genetics, 411 Borlaug Hall, University of Minnesota, Saint Paul, MN 55108
Phone: 612-625-7773
Email: pmorrell@umn.edu

DOI: 10.1534/g3.114.010561

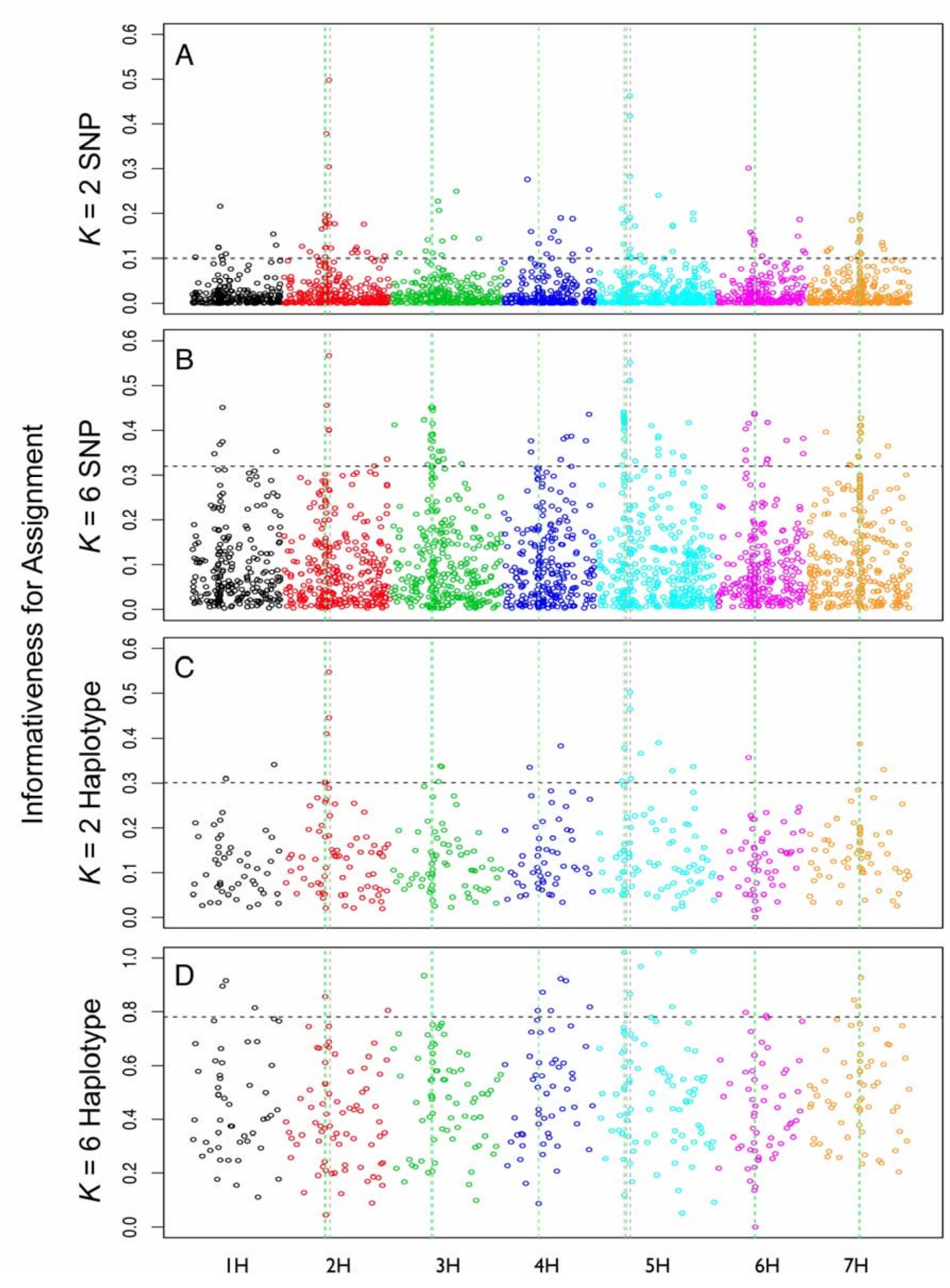

Figure S1 The informativeness for assignment for all SNPs (A, B) and five-SNP haplotypes (C, D) genome-wide based on (A, C) *K* = 2 and (B, D) *K* = 6. The horizontal dashed line is the 95th percentile. The grey vertical dashed lines delineate the two high F_{ST} regions based on comparison between the Eastern and Western populations. The green vertical dashed lines indicate the centromeric regions. Note the scale of y-axis in panel D.

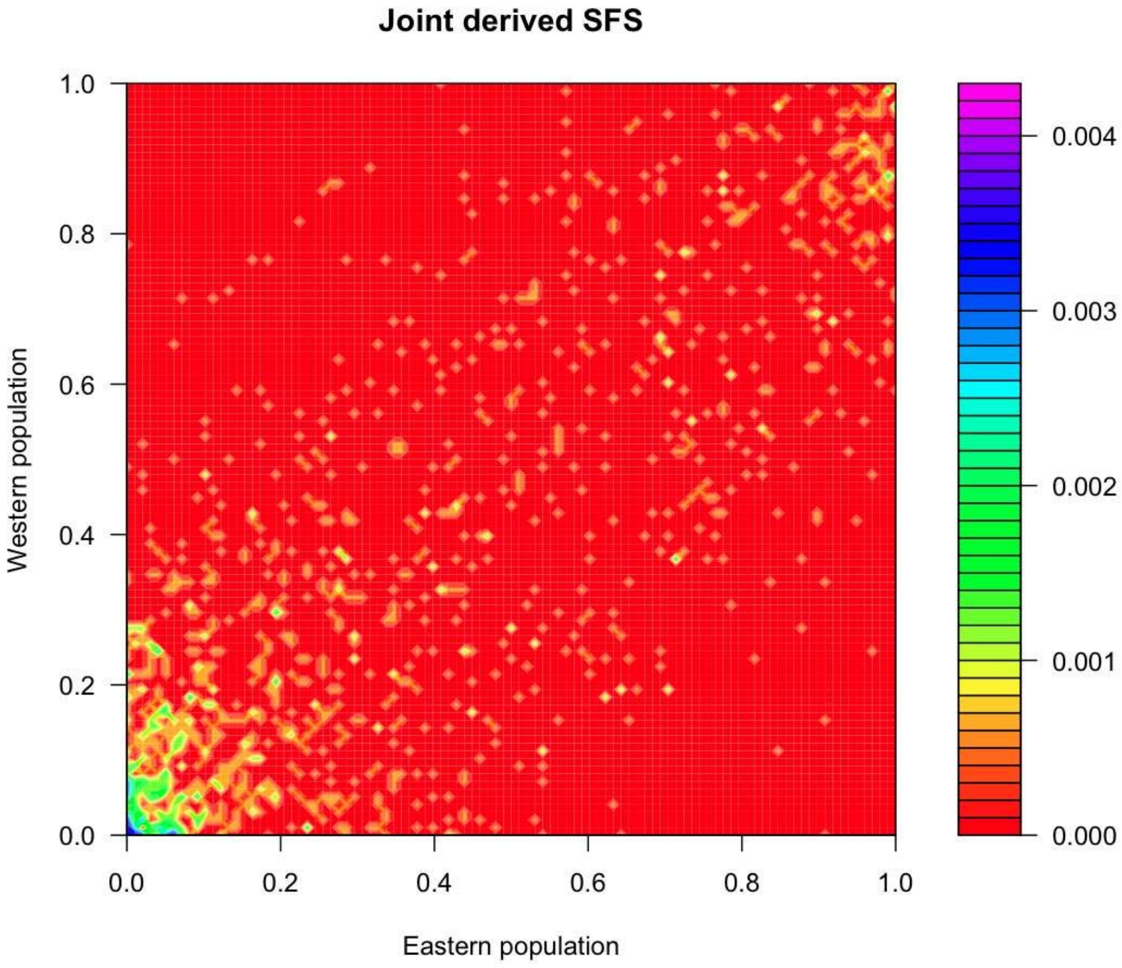

Figure S2 The joint unfolded site frequency spectrum based on all accessions from the Eastern population (upper triangle) and Western population (lower triangle). The comparison includes 1633 SNPs for which the ancestral state could be inferred by comparison to *Hordeum bulbosum* Illumina RNA-Seq data.

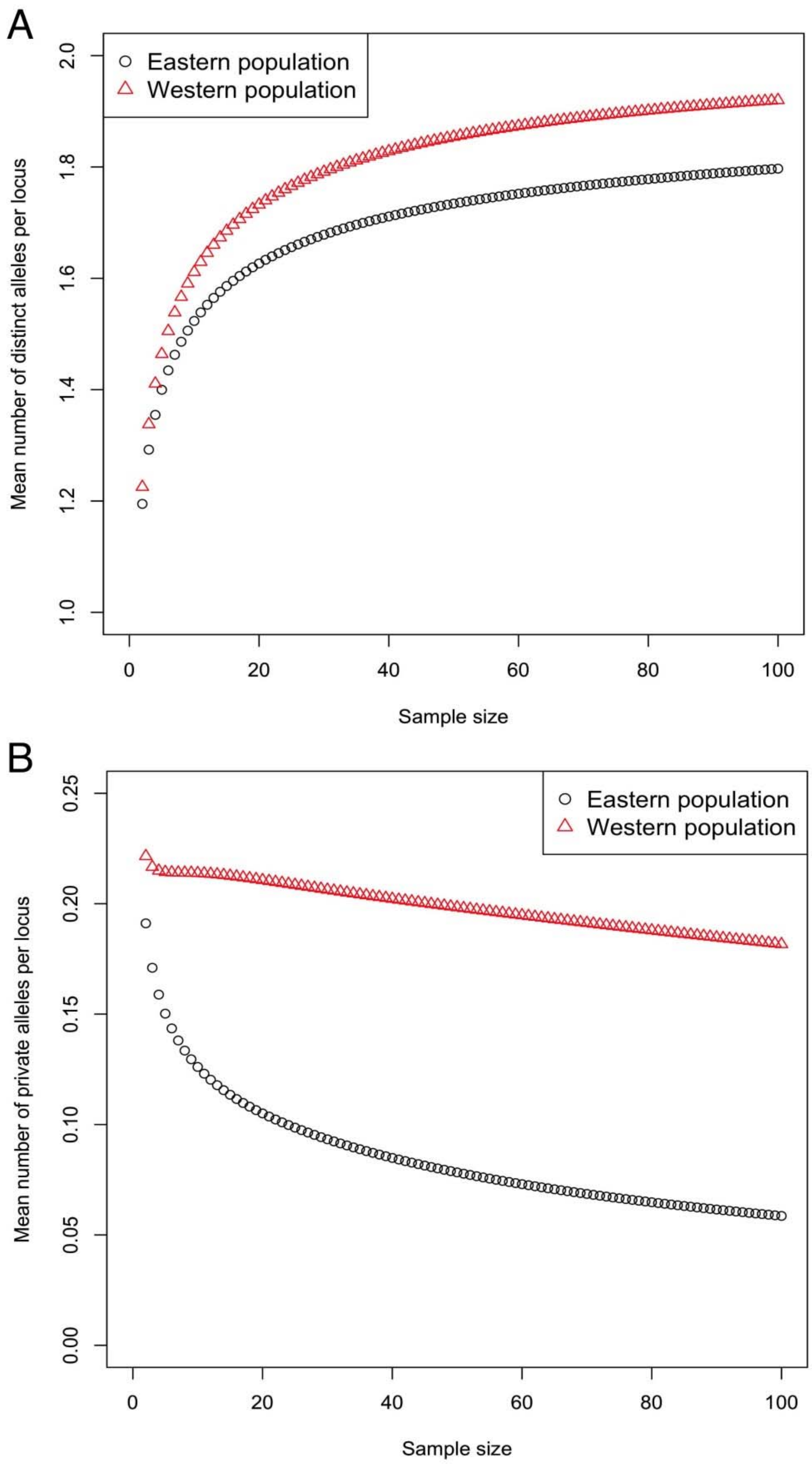

Figure S3 Rarefaction analysis comparing nucleotide diversity between the Eastern and Western populations, (A) mean number of distinct alleles per locus and (B) mean number of private alleles per locus.

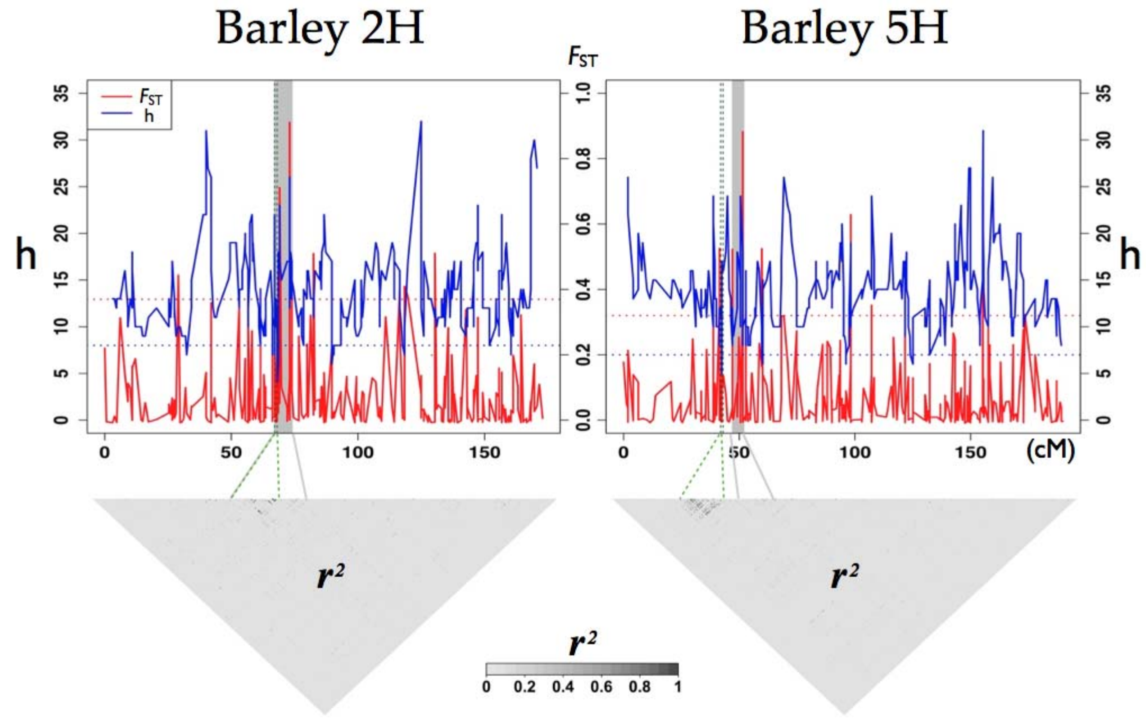

Figure S4 Population genetic analysis of the two high F_{ST} regions. Top panel, haplotype number (blue curve) and F_{ST} between the Eastern and Western populations (red curve). The number of haplotypes present across linkage group 2H and 5H was calculated in overlapping 5-SNP windows with 1-SNP increments. The high F_{ST} regions are marked in grey and the centromeres by green dashed lines. Below, LD (r^2) is plotted across 2H and 5H.

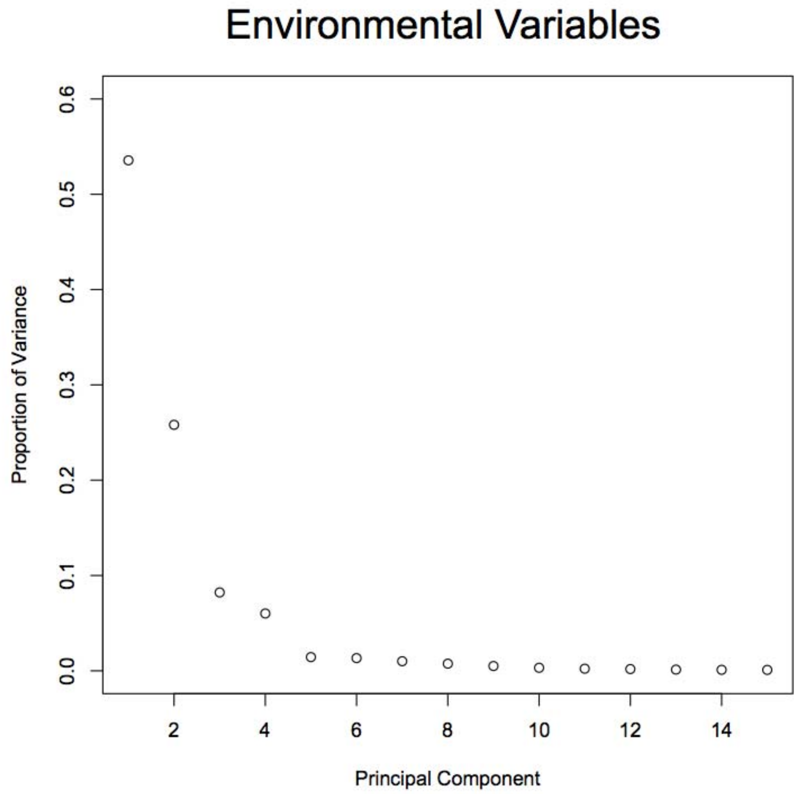

Figure S5 The proportion of variance explained by each PC of environmental variables.

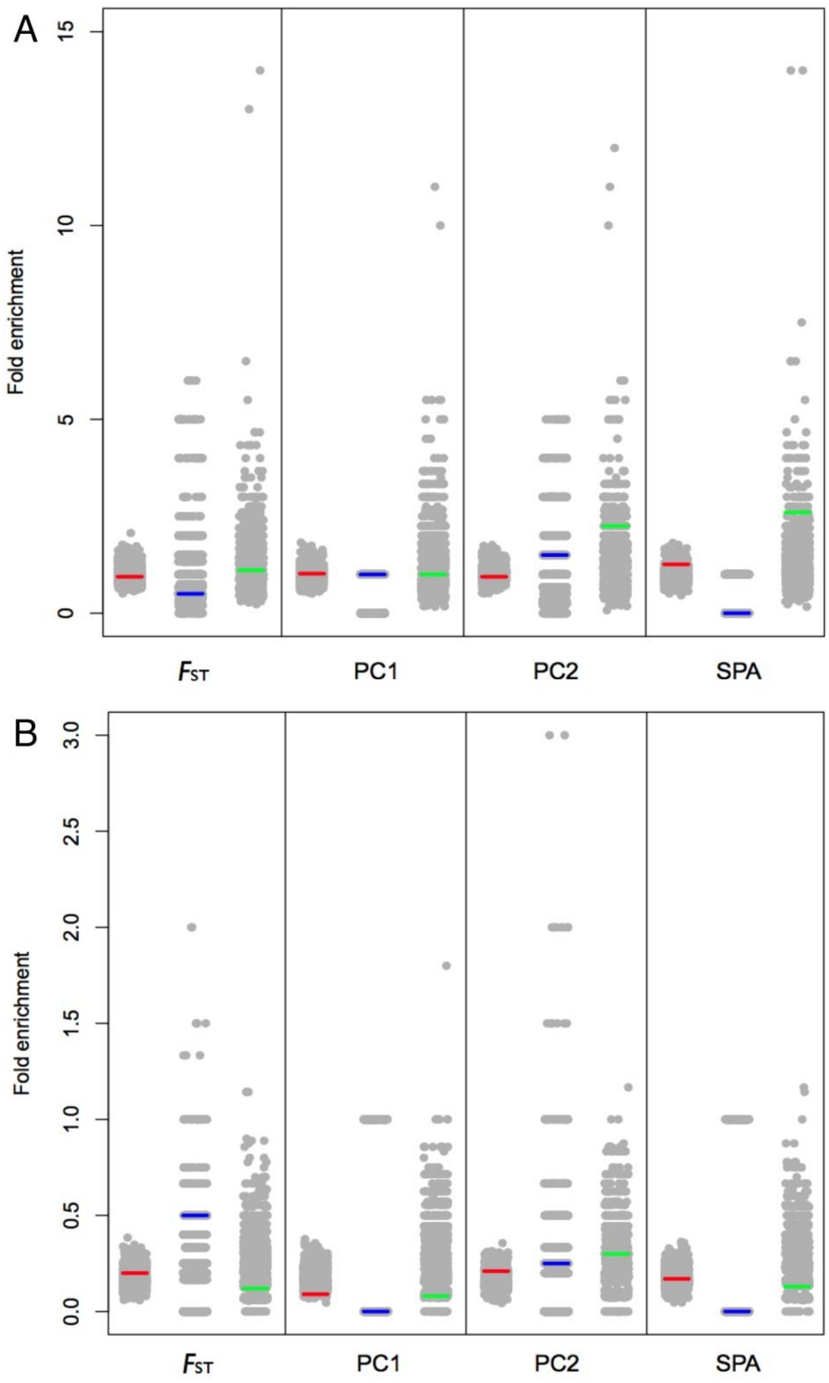

Figure S6 Enrichment analysis for (A) genic versus non-genic and (B) nonsynonymous versus synonymous SNPs. Enrichment analysis for candidates involved resampling the number of SNPs in each candidate list (outliers in F_{ST}, environmental association or SPA analyses) randomly from the genome 1000 times. Enrichment analysis was performed for all SNPs genome-wide, centromeric regions, and the two putative translocations or inversions. The observed values from the data is indicated by the colored horizontal line, red: genome-wide; blue: centromeric regions; green: the two putative structural rearrangements.

Table S1 284 wild barley accessions used in this study, including their latitude and longitude information

Accession	Latitude	Longitude
WBDC001	36.22	36.7
WBDC002	35.71	37.44
WBDC004	35.97	36.54
WBDC005	32.45	35.92
WBDC006	32.3	36.17
WBDC007	32.33	35.92
WBDC008	32.67	35.62
WBDC009	31.53	35.75
WBDC010	36.67	65.73
WBDC011	36	43.52
WBDC012	36.28	64.82
WBDC013	35.53	44.83
WBDC014	35.75	69
WBDC015	34.35	62.18
WBDC017	32.74	35.82
WBDC018	34.98	63.12
WBDC019	36.75	45.72
WBDC021	34.8	45.6
WBDC022	39.78	30.52
WBDC023	33.5	48.45
WBDC024	31.28	48.72
WBDC025	30.3	66.9
WBDC026	37.65	69.1
WBDC028	31.95	34.93
WBDC029	31.58	34.62
WBDC030	33.08	35.18
WBDC031	32.75	35.08
WBDC032	33	35.13
WBDC033	31.27	35.22
WBDC034	31.85	34.92
WBDC035	31.43	34.59
WBDC036	34.57	63
WBDC037	32.98	35.5
WBDC038	46.77	35.21
WBDC039	32.03	35.83
WBDC040	32.7	34.95
WBDC041	31.67	34.57
WBDC042	32.97	35.53
WBDC043	32.98	35.58
WBDC044	32.17	34.83
WBDC046	32.48	35.9
WBDC047	32.53	36.07
WBDC048	37.25	44.48
WBDC049	37.25	44.48
WBDC051	34.76	39.03
WBDC052	32.32	36.02

WBDC054	34.57	38.36
WBDC055	35.61	35.82
WBDC056	36.72	37.12
WBDC058	34.99	34.02
WBDC059	34.98	34.05
WBDC060	31.35	27.17
WBDC061	36.16	36.58
WBDC062	36.38	36.84
WBDC063	36.72	36.64
WBDC064	35.8	36.24
WBDC066	33.78	36.4
WBDC067	33.7	36.38
WBDC068	32.82	36.72
WBDC070	35.61	35.82
WBDC072	32.78	21.63
WBDC073	32.08	21.87
WBDC074	32.8	22.05
WBDC075	32.7	21.92
WBDC078	36.77	40.86
WBDC079	32.25	35.88
WBDC080	32.53	35.78
WBDC081	32.48	35.65
WBDC083	32.17	35.92
WBDC085	31.98	35.65
WBDC089	31.83	35.8
WBDC095	31.18	35.62
WBDC097	31.28	35.83
WBDC100	30.77	35.62
WBDC101	30.7	35.57
WBDC102	30.58	35.57
WBDC103	30.2	35.47
WBDC104	31.18	35.62
WBDC105	32.65	35.83
WBDC106	34.94	36.74
WBDC107	34.75	36.73
WBDC108	33.94	36.71
WBDC109	32.77	36.72
WBDC110	32.78	36.79
WBDC111	33.84	36.54
WBDC112	33.98	36.59
WBDC113	37.92	58.17
WBDC115	38.33	55.87
WBDC116	38.32	56.38
WBDC117	38.17	56.05
WBDC119	40.08	67.58
WBDC120	39.47	67.5
WBDC121	29.72	51.78
WBDC122	32.87	48.17
WBDC123	36.42	58.47

WBDC124	35.02	46.45
WBDC125	38.8	66.47
WBDC126	33.45	35.32
WBDC127	32.94	36.73
WBDC128	33.84	36.17
WBDC129	32.81	36.42
WBDC130	32.83	36.18
WBDC131	33.01	36.04
WBDC132	33.47	35.87
WBDC133	33.62	35.82
WBDC134	33.62	35.77
WBDC135	33.52	35.72
WBDC136	33.52	35.77
WBDC137	33.45	35.82
WBDC138	33.42	35.76
WBDC139	33.93	36.1
WBDC140	34.2	36.08
WBDC141	34.13	36.03
WBDC142	34.02	36.08
WBDC143	33.57	35.9
WBDC145	33.8	36.02
WBDC146	36.75	45.7
WBDC147	37.5	45.17
WBDC148	37.07	45.47
WBDC149	38.08	45
WBDC150	38.5	47.2
WBDC151	36.52	36.95
WBDC152	35.5	51.25
WBDC155	36.33	42.17
WBDC156	36.42	41.65
WBDC157	36.38	43.42
WBDC158	35.37	43.2
WBDC159	32.68	36.79
WBDC160	32.96	36.6
WBDC161	36.65	37.58
WBDC164	37.01	41.64
WBDC165	37.29	42.21
WBDC167	36.4	40.36
WBDC168	33.57	35.72
WBDC169	33.5	35.78
WBDC170	33.63	35.95
WBDC171	33.58	35.85
WBDC174	36.27	47.77
WBDC175	36.27	47.77
WBDC177	36.35	43.13
WBDC178	36.08	43.28
WBDC179	32.5	20.9
WBDC180	31.83	24.23
WBDC181	32.02	36.02

WBDC183	32.63	35.72
WBDC184	32.37	21.17
WBDC185	32.77	21.72
WBDC186	36.88	37.35
WBDC187	36.69	37.46
WBDC188	36.88	37.52
WBDC189	36.76	37.63
WBDC191	36.84	37.72
WBDC192	37.04	37.61
WBDC193	37.32	37.47
WBDC194	36.97	37.19
WBDC195	36.81	37.22
WBDC196	36.99	36.93
WBDC197	36.48	37.77
WBDC198	34.82	36.47
WBDC199	34.91	36.64
WBDC200	34.94	36.66
WBDC201	35.56	36.89
WBDC202	35.6	36.55
WBDC203	35.63	36.45
WBDC204	38.58	57.12
WBDC205	42.05	48.27
WBDC206	32.55	36.6
WBDC207	40.34	71.18
WBDC208	41.61	69.91
WBDC209	40.13	68.4
WBDC210	39.93	67.68
WBDC212	40.01	67.09
WBDC213	39.55	66.56
WBDC214	39.4	67.02
WBDC215	38.28	56.29
WBDC216	38.73	56.85
WBDC217	40.2	44.53
WBDC218	43.06	73.66
WBDC220	42.42	69.7
WBDC221	40.13	69.33
WBDC222	40.13	69.33
WBDC223	39.97	69
WBDC225	40.12	69.2
WBDC228	40.49	49.19
WBDC229	40.53	48.89
WBDC230	40.61	48.88
WBDC231	40.71	48.8
WBDC232	40.7	48.63
WBDC233	35.93	68.7
WBDC234	35.15	33.88
WBDC236	31.6	35.62
WBDC237	31.53	35.62
WBDC238	31.55	35.85

WBDC240	31.88	35.94
WBDC241	31.78	36.22
WBDC242	32.12	35.87
WBDC243	32.03	35.67
WBDC244	32.08	35.71
WBDC245	32.17	35.87
WBDC246	32.68	35.75
WBDC247	32.37	35.7
WBDC248	32.53	35.85
WBDC250	32.58	35.93
WBDC252	32.28	35.68
WBDC253	32.35	35.82
WBDC254	32.62	35.7
WBDC255	32.35	35.67
WBDC256	32.33	35.63
WBDC257	31.18	35.7
WBDC258	31.28	35.75
WBDC260	30.5	35.5
WBDC261	30.52	35.53
WBDC262	30.3	35.48
WBDC263	30.38	35.47
WBDC265	30.93	35.67
WBDC266	30.88	35.67
WBDC268	31.75	36.76
WBDC269	33.42	35.87
WBDC270	31.68	34.87
WBDC271	31.43	34.48
WBDC274	30.87	34.77
WBDC275	31.58	34.55
WBDC277	32.15	34.93
WBDC278	31.83	34.77
WBDC279	31.82	34.78
WBDC280	32.02	34.83
WBDC281	33.03	35.58
WBDC282	32.68	35.22
WBDC284	32.67	35.6
WBDC285	32.98	35.45
WBDC286	32.97	35.47
WBDC287	32.5	35.5
WBDC288	32.98	35.42
WBDC289	32.52	35.48
WBDC290	31.67	34.93
WBDC291	31.72	34.92
WBDC292	31.8	35.02
WBDC293	31.8	35.17
WBDC294	31.75	34.98
WBDC295	37.08	42.07
WBDC296	37.06	41.56
WBDC297	37.06	41.75

WBDC298	37.08	41.09
WBDC299	36.66	36.71
WBDC300	36.37	36.86
WBDC302	33.37	35.88
WBDC303	33.74	36.11
WBDC304	33.64	35.96
WBDC305	33.66	36
WBDC306	33.73	36.13
WBDC307	33.2	36.07
WBDC308	32.6	36.38
WBDC309	35.03	37.01
WBDC310	34.54	36.92
WBDC312	34.47	36.76
WBDC315	34.94	36.85
WBDC316	36.23	36.55
WBDC317	35.52	38.76
WBDC318	32.6	36.74
WBDC319	32.49	36.68
WBDC320	32.42	36.62
WBDC323	37.9	58.55
WBDC324	35.83	61.46
WBDC326	37.72	58.6
WBDC329	38.18	55.6
WBDC330	38.77	56.29
WBDC331	39.24	56.11
WBDC332	38.43	56.42
WBDC333	38.41	56.49
WBDC334	38.27	56.31
WBDC335	39.25	55.62
WBDC336	38.42	56.68
WBDC337	37.23	37.52
WBDC338	37.27	37.54
WBDC340	37.25	37.33
WBDC341	36.95	36.93
WBDC342	36.97	36.9
WBDC343	36.87	36.95
WBDC345	38.95	66.83
WBDC346	39.92	66.37
WBDC347	37.8	67
WBDC348	32.44	35
WBDC349	32.14	35.14
WBDC355	40.31	48.81

Table S2 The name, repeat length, repeat unit length, total size and observed heterozygosity of the 29 microsatellites used in this study

Microsatellite	# Repeat	Repeat unit length	Total size (bp)	Observed Heterozygosity
Bmag905	14	2	178-228	0.01
Bmag006	17	2	105-223	0.10
Bmac129	28	2	132-204	0.00
Bmac67	18	2	130-262	0.04
Bmag749	11	2	93-199	0.01
Bmac134	28	2	117-189	0.01
Bmac156	(AC)22(AT)5	2	103-199	0.01
Bmac213	23	2	131-213	0.09
Bmac316	19	2	138-234	0.01
Bmag369	16	2	196-216	0.00
Bmag718	(GA)18(AG)6	2	165-217	0.02
Bmag877	15	2	153-289	0.04
EBmac603	10	2	155-257	0.09
HVM06	9	2	118-202	0.23
HVMLOH1A	6	2	147-203	0.00
GMS1	(CT)7TTT(CT)2	2	123-161	0.18
Bmag0496	20	2	139-287	0.03
HVHVA1	5	3	134-140	0.01
Bmac18	11	2	131-145	0.00
Bmag382	(AG)7AA(AG)7	2	103-109	0.00
Scssr02748	12	2	144-158	0.01
Scssr10148	10	2	178-230	0.04
Scssr08447	6	3	172-182	0.00
Scssr05939	5	2	150-160	0.03
Bmag211	16	2	150-198	0.02
Hvltppb	10	2	205-229	0.75
Scssr02306	13	2	150-164	0.00
Scssr15864	4	3	146-184	0.08
Scssr25691	17	2	208-250	0.07

Table S3 Morex state and ancestral state (inferred using *H. Bulbosum* accession Cb2920/4) for all BOPA SNPs. Available for download as a .txt file at http://www.g3journal.org/lookup/suppl/doi:10.1534/g3.114.010561/-/DC1

Table S4 (A) Environmental variables and abbreviations used in this study; (B) Environmental variable and the corresponding loadings for the first two principal components

(A)

**Environmental variable**	**Abbreviation**
Annual mean temperature	bio1
Mean diurnal range	bio2
Isothermality (bio2/bio7)*100 [a]	bio3
Temperature seasonality (standard deviation*100)	bio4
Min temperature of the coldest month	bio6
Mean temperature of the wettest quarter	bio8
Mean temperature of the driest quarter	bio9
Mean temperature of the coldest quarter	bio11
Annual precipitation	bio12
Precipitation of the wettest month	bio13
Precipitation of the driest month	bio14
Precipitation seasonality (coefficient of variation)	bio15
Precipitation of the wettest quarter	bio16
Precipitation of the driest quarter	bio17
Precipitation of the coldest quarter	bio19
Monthly minimum and maximum temperature	tmin#, tmax#
Monthly total precipitation	prec#

[a] bio7: temperature annual range

(B)

PC1		PC2	
Variable	Loadings	Variable	Loadings
bio11	-0.223	bio12	-0.316
tmin2	-0.222	bio16	-0.314
tmax1	-0.222	bio13	-0.310
tmax2	-0.221	prec2	-0.306
tmax12	-0.221	bio19	-0.303
tmax11	-0.220	prec1	-0.297
bio6	-0.220	prec12	-0.295
tmin1	-0.220	prec11	-0.292
tmin3	-0.220	prec3	-0.286
tmin12	-0.218	prec10	-0.180
tmin11	-0.215	prec4	-0.128
tmin10	-0.215	alt	-0.089
tmax3	-0.211	bio15	-0.058

bio1	-0.209	tmin11	-0.039
tmax10	-0.201	tmin12	-0.038
tmin4	-0.196	tmin1	-0.031
tmax4	-0.179	bio6	-0.030
bio3	-0.178	tmin10	-0.019
bio15	-0.176	tmin2	-0.013
bio9	-0.116	bio2	0.199
bio8	-0.111	tmax4	0.147
prec12	-0.080	bio8	0.091
prec1	-0.079	bio4	0.088
bio19	-0.068	tmax3	0.086
prec11	-0.058	bio9	0.086
bio13	-0.052	tmin4	0.071
bio16	-0.048	tmax10	0.068
prec2	-0.047	bio1	0.065
bio12	0.003	tmax2	0.047
prec3	0.020	bio3	0.041
bio2	0.026	tmax11	0.027
prec10	0.091	tmax1	0.023
bio14	0.112	tmax12	0.022
bio17	0.120	tmin3	0.018
prec4	0.141	bio14	0.012
alt	0.154	bio17	0.004
bio4	0.162	bio11	0.003

Table S5 BOPA SNPs used in this study, including their genetic positions

SNP	Chr	Position
11_20001	4H	31.69
11_20002	3H	65.25
11_20003	5H	121.67
11_20005	6H	132.6
11_10002	1H	64.44
11_10003	6H	56.06
11_20008	5H	141.48
11_20009	3H	118.22
11_10005	3H	71.26
11_10006	1H	76.92
11_20010	5H	12.8
11_10008	3H	67.86
11_20012	4H	43.72
11_20013	4H	146.48
11_20015	6H	74.65
11_20014	7H	11.87
11_20017	3H	82.62
11_20018	5H	93.66
11_10010	4H	77.66
11_20020	4H	63.79
11_10011	3H	67.86
11_20021	1H	111.81
11_20023	3H	122.7
11_20022	5H	177.9
11_10012	2H	67.08
11_10015	6H	111.08
11_10017	2H	81.31
11_20029	6H	133.25
11_20032	2H	68.07
11_10023	6H	24.91
11_10024	5H	96.12
11_20036	6H	118.15
11_20039	2H	67.63
11_10025	7H	14.39
11_10026	3H	34.15
11_20042	7H	88.06
11_10028	4H	56.22
11_20044	4H	115.37
11_10030	1H	17.4
11_20052	6H	44.96
11_10031	4H	30.37
11_20053	6H	81.79
11_20058	6H	65.83
11_20060	7H	65.98
11_20064	2H	124.98
11_20063	3H	97.14
11_20072	4H	78.08

11_10041	1H	138.86
11_20078	5H	155.66
11_20080	2H	107.04
11_10043	1H	63.74
11_10044	3H	134.71
11_20085	3H	146.25
11_20086	2H	110.93
11_10046	4H	62.81
11_20089	4H	145.7
11_10047	3H	90.1
11_20092	7H	111.15
11_10048	4H	46.97
11_20093	3H	93.81
11_20095	1H	58.59
11_10050	7H	57.57
11_20096	5H	70.9
11_10052	4H	76.31
11_20097	5H	88.05
11_20102	3H	65.25
11_20103	7H	104.32
11_20104	5H	149.94
11_20105	5H	49.83
11_20107	2H	14.42
11_10055	7H	81.78
11_20109	4H	29.34
11_10056	7H	33.49
11_10057	2H	67.08
11_10058	5H	42.41
11_10061	6H	44.96
11_20113	7H	49.61
11_20114	4H	44.99
11_20115	3H	92.73
11_20117	7H	156.13
11_20118	6H	106.15
11_20119	4H	109.65
11_20121	1H	80.47
11_20126	7H	31.35
11_10069	7H	83.42
11_10070	2H	66.2
11_20127	5H	116.66
11_10072	2H	165.28
11_20129	5H	42.41
11_20130	3H	108.7
11_20131	2H	54.27
11_10075	1H	45.2
11_20133	1H	132.16
11_20134	5H	95.11
11_20135	4H	63.79
11_10080	5H	143.29

11_20136	3H	100.29
11_20138	1H	140.69
11_20139	7H	141.15
11_10081	3H	39.92
11_20145	4H	1.2
11_20149	1H	98.68
11_20153	1H	105.77
11_10090	4H	86.01
11_20155	3H	162.83
11_10092	2H	137.03
11_10093	4H	56.22
11_10094	5H	114.14
11_20159	3H	6.31
11_20160	2H	68.07
11_10095	5H	132.32
11_20162	7H	26.14
11_20168	3H	124.04
11_20170	7H	162.45
11_20172	3H	18.21
11_10101	2H	170.73
11_10104	5H	141.88
11_20178	4H	95.22
11_20179	5H	42.41
11_20180	4H	44.99
11_20182	2H	127.74
11_20184	6H	61.19
11_10112	3H	14
11_20189	5H	172.83
11_20192	7H	29.7
11_10113	4H	19.45
11_20193	3H	42.31
11_20195	7H	67.02
11_10116	5H	41.45
11_20197	4H	90.77
11_20200	7H	81.78
11_10120	6H	6.54
11_10121	7H	2.13
11_10124	6H	74.65
11_20205	7H	83.42
11_10128	2H	124.98
11_10129	6H	44.96
11_10130	7H	149.31
11_20210	4H	26.71
11_10132	4H	26.2
11_20211	6H	133.9
11_20212	6H	1.03
11_20215	2H	147.37
11_20219	0	0
11_20220	1H	111.15

11_10136	6H	27.33
11_10137	3H	67.86
11_10138	2H	108.58
11_20222	3H	91.79
11_20226	5H	1.91
11_20230	7H	88.06
11_10143	7H	91.12
11_20232	6H	0
11_10147	2H	60.89
11_20236	5H	68.21
11_10153	7H	79.08
11_10157	5H	42.41
11_10158	3H	73.3
11_10161	5H	155.45
11_10165	6H	23.62
11_20245	7H	7.87
11_10167	5H	71.93
11_20247	7H	116.28
11_20249	7H	44.12
11_10169	7H	106.8
11_20251	2H	68.56
11_10172	3H	82.62
11_10174	7H	162.03
11_10175	6H	130.38
11_10177	5H	42.41
11_10178	2H	40.03
11_20259	5H	125.09
11_10180	2H	20.45
11_20260	1H	42.42
11_20262	6H	10.72
11_10182	7H	129.32
11_10184	3H	110.75
11_10185	6H	93.44
11_10186	1H	22.04
11_20265	5H	53.84
11_20266	6H	65.29
11_10189	6H	65.83
11_10191	2H	72.99
11_20267	1H	105.77
11_10194	2H	67.08
11_20269	4H	54.73
11_10196	2H	89.68
11_20273	3H	83.58
11_10202	6H	102.62
11_20276	3H	71.26
11_20283	5H	51.51
11_10208	4H	2.6
11_10213	2H	99.39
11_10214	2H	105.62

11_20287	6H	73.84
11_20288	3H	65.25
11_20289	4H	58.2
11_10216	2H	26.63
11_10217	5H	149.94
11_20291	6H	52.85
11_20293	2H	161.3
11_20294	6H	6.54
11_10223	4H	19.45
11_10224	3H	67.27
11_10225	3H	69.4
11_20298	5H	125.09
11_20300	5H	122.33
11_10232	7H	29.05
11_10234	2H	54.27
11_20303	7H	0
11_10236	5H	172.83
11_20306	5H	50.53
11_20307	7H	6.79
11_20309	0	0
11_10240	5H	42.41
11_10243	2H	67.08
11_20311	0	0
11_10244	6H	48.42
11_20315	6H	27.33
11_10247	4H	87.01
11_10251	0	0
11_10252	5H	42.41
11_10253	3H	102.66
11_10254	5H	169.72
11_20327	5H	93.22
11_10256	7H	79.08
11_10259	1H	40.4
11_10260	5H	38.78
11_10261	4H	53.94
11_10262	4H	63.44
11_20332	5H	49.16
11_10265	2H	80.14
11_20333	3H	67.86
11_20334	5H	159.97
11_20336	0	0
11_10269	4H	138.7
11_10275	1H	35.77
11_10276	3H	86.57
11_20340	2H	98.74
11_10279	1H	77.57
11_20343	3H	136.92
11_10280	3H	136.92
11_10281	3H	73.3

11_10283	3H	180.12
11_20349	7H	86.84
11_20347	5H	121.67
11_10287	2H	98.74
11_20354	7H	125.16
11_20355	6H	120.69
11_20356	3H	50.85
11_10292	5H	139.63
11_20358	4H	96.42
11_10293	1H	48.99
11_10294	1H	40.4
11_10299	7H	72.18
11_20362	3H	90.52
11_20365	7H	162.24
11_10303	7H	89.78
11_20366	2H	145.69
11_10309	4H	85.07
11_10310	5H	183.14
11_10312	3H	125.11
11_20372	5H	50.53
11_20373	0	0
11_10315	2H	158.18
11_20375	5H	139
11_10317	2H	68.56
11_10318	5H	42.41
11_10319	4H	11.04
11_10323	6H	59.25
11_20379	6H	115.31
11_20383	1H	134.96
11_20384	4H	99.36
11_20385	7H	111.15
11_10325	2H	56.64
11_10326	2H	4.72
11_10329	2H	168.26
11_20386	5H	24.76
11_20387	2H	59.58
11_10331	6H	92.69
11_10332	1H	11.66
11_20388	5H	146.55
11_10334	4H	113.95
11_10335	3H	77.37
11_10336	5H	157.61
11_10338	1H	118.98
11_20390	2H	72.99
11_20392	5H	60.21
11_20394	2H	26.63
11_10343	3H	179.81
11_10342	2H	44.82
11_10352	0	0

11_10357	1H	103.99
11_10360	5H	121.67
11_10363	5H	147.49
11_10365	3H	65.25
11_20409	3H	143.33
11_20410	3H	39.92
11_20411	4H	30.37
11_20412	4H	64.64
11_10370	7H	81.78
11_20415	6H	15.81
11_10373	3H	73.97
11_20419	2H	85.52
11_10376	2H	149.27
11_10379	4H	58.2
11_20422	4H	28
11_10380	3H	63.25
11_10381	3H	143.33
11_10383	2H	147.37
11_20427	1H	42.42
11_20428	3H	67.86
11_10385	5H	155.23
11_20432	1H	62.05
11_10387	4H	130.81
11_20434	1H	89.77
11_20438	2H	69.05
11_20439	3H	67.27
11_20441	5H	54.77
11_10390	6H	137.41
11_10394	7H	81.78
11_20444	3H	68.51
11_10396	1H	98.68
11_20450	4H	64
11_20449	5H	90.07
11_20451	4H	79.47
11_10399	2H	40.03
11_20452	7H	146.27
11_10400	6H	100.68
11_20453	4H	71.71
11_10401	5H	187.2
11_20454	4H	111.13
11_20458	2H	68.07
11_10404	2H	133.71
11_10405	5H	172.83
11_20460	7H	82.2
11_20461	5H	42.41
11_10409	4H	3.2
11_10411	4H	56.22
11_20467	6H	118.15
11_10414	5H	93.22

11_20472	4H	64
11_20475	1H	93.48
11_20476	2H	67.08
11_10419	1H	4.71
11_20479	0	0
11_10422	2H	56.64
11_20482	4H	69.24
11_10424	4H	56.22
11_20487	5H	130.32
11_20485	7H	91.67
11_20486	3H	66.62
11_20488	6H	84.47
11_10427	6H	37.99
11_20493	6H	1.03
11_20494	2H	155.68
11_10429	2H	130.38
11_10431	7H	67.02
11_20495	7H	18.73
11_10432	4H	54.73
11_10433	1H	95.14
11_10434	1H	88.88
11_20498	2H	116.5
11_20500	2H	63.55
11_10436	2H	72.99
11_10438	1H	42.42
11_20501	5H	51.51
11_20502	1H	2.55
11_20504	7H	158.84
11_10442	7H	85.28
11_20507	7H	27.09
11_20509	0	0
11_20511	2H	137.03
11_10443	1H	143.2
11_10444	3H	98.05
11_10446	2H	140.69
11_20513	0	0
11_20514	1H	35.98
11_20515	4H	112.22
11_20521	3H	86.03
11_10451	7H	27.09
11_20523	3H	125.11
11_20524	5H	42.41
11_20526	5H	86.08
11_20527	3H	142.17
11_20529	3H	11.01
11_10454	7H	139.9
11_10455	6H	73.84
11_20531	6H	111.08
11_10456	3H	67.86

11_20532	2H	72.99
11_20533	5H	10.08
11_20534	7H	4.5
11_20536	5H	168.44
11_20537	6H	142.2
11_10460	1H	3.21
11_10461	6H	52.19
11_10462	6H	49.17
11_20545	5H	154.51
11_20546	5H	163.72
11_20549	5H	95.11
11_10471	1H	89.77
11_20550	1H	90.31
11_10466	1H	84.71
11_20551	5H	138.25
11_20552	3H	26.23
11_10467	4H	82.81
11_20553	5H	1.91
11_20557	4H	20.87
11_20558	6H	122.14
11_10469	6H	81.79
11_20560	5H	156.27
11_10470	1H	42.42
11_20561	2H	169.66
11_20562	2H	10.86
11_20563	2H	10.07
11_10475	2H	101.3
11_20568	5H	143.29
11_10477	5H	107.19
11_10480	4H	58.2
11_20570	7H	112.35
11_20571	5H	38.78
11_10481	5H	42.41
11_20572	6H	59.25
11_20573	5H	152.93
11_20577	6H	80.06
11_20580	4H	79.47
11_10490	4H	12.34
11_20582	5H	6.36
11_20583	3H	66.62
11_20584	0	0
11_20585	2H	72.03
11_10494	6H	44.96
11_10496	0	0
11_10498	2H	53.09
11_20590	2H	154.65
11_20594	1H	139.6
11_10509	4H	56.22
11_20595	3H	14.91

11_10510	4H	112.88
11_10513	6H	60.65
11_20600	6H	59.25
11_10515	3H	110.09
11_10516	1H	63.14
11_10518	5H	88.05
11_20606	4H	28
11_20607	3H	32.92
11_10520	1H	45.2
11_20609	0	0
11_20610	4H	61.56
11_10522	1H	101.34
11_10523	4H	88.3
11_10525	2H	38.86
11_10527	4H	65.28
11_20612	3H	139.66
11_10528	5H	155.45
11_10531	7H	85.28
11_10534	7H	82.41
11_20617	1H	27.12
11_10536	5H	155.23
11_10538	2H	133.04
11_10539	6H	50.33
11_20620	6H	78.52
11_20623	0	0
11_20625	1H	110.08
11_20626	3H	109.43
11_20628	3H	108.7
11_20629	5H	114.82
11_20631	2H	67.08
11_10547	7H	160.97
11_10551	2H	156.77
11_10552	1H	54.54
11_20636	6H	77.53
11_20637	5H	115.76
11_10557	5H	144.6
11_10559	3H	26.71
11_20639	3H	71.26
11_10578	5H	83.8
11_10563	7H	112.99
11_10566	2H	157.26
11_20642	1H	62.6
11_10568	4H	62.15
11_20644	5H	164.15
11_20645	5H	74.83
11_20646	5H	157.61
11_20647	3H	43.96
11_20650	3H	135.43
11_10574	4H	21.53

11_20651	6H	58.48
11_10576	7H	35.36
11_20652	7H	111.15
11_10577	4H	54.73
11_20653	5H	121.67
11_20654	6H	95.7
11_20656	6H	58.48
11_10580	5H	29.9
11_10582	5H	155.66
11_20659	3H	102.12
11_20660	1H	42.42
11_10584	3H	127.26
11_20662	3H	136.92
11_10586	1H	123.1
11_20666	3H	48.02
11_10588	4H	97.52
11_20668	4H	145.7
11_20669	2H	68.56
11_10589	5H	149.94
11_20670	4H	90.11
11_20671	7H	64.98
11_10590	1H	141.46
11_20673	6H	78.52
11_20674	2H	55.46
11_10593	5H	164.36
11_20676	5H	141.88
11_20675	6H	53.54
11_10595	6H	107.49
11_10597	1H	36.64
11_20680	4H	28.75
11_10614	4H	111.81
11_20681	2H	169.66
11_20682	6H	84.47
11_10602	2H	63.55
11_20686	5H	163.72
11_20687	6H	139.09
11_20690	2H	78.54
11_20691	7H	143.13
11_10606	4H	78.08
11_10608	6H	88.25
11_20694	3H	84.98
11_20695	3H	87.8
11_10610	4H	139.97
11_20697	5H	41.87
11_10611	4H	135.58
11_20698	1H	42.42
11_20700	5H	42.41
11_20701	4H	133.52
11_10617	1H	59.29

11_20704	3H	76.43
11_20707	6H	49.67
11_20708	5H	42.41
11_20709	6H	74.65
11_10621	5H	31.2
11_10619	2H	95.58
11_20712	1H	20.33
11_10624	2H	67.08
11_20713	5H	56.77
11_20714	6H	75.28
11_10627	4H	79.47
11_10625	2H	156.77
11_20715	2H	151.92
11_20719	3H	52.17
11_20720	6H	57.18
11_10630	2H	117.59
11_10631	3H	153.61
11_20722	7H	13.7
11_20723	4H	73.62
11_20724	2H	14.98
11_20725	6H	118.15
11_10632	2H	73.35
11_10638	2H	57.29
11_10639	4H	76.09
11_20730	5H	33.71
11_10641	5H	59.72
11_20732	4H	100.23
11_20733	6H	122.74
11_20734	2H	86.58
11_20736	5H	72.68
11_20737	5H	47.04
11_10644	1H	128.63
11_10645	6H	129.73
11_10646	3H	167.33
11_20740	4H	82.81
11_10648	2H	42.78
11_10651	2H	79.82
11_20742	3H	20.52
11_20743	6H	48.42
11_20744	6H	80.86
11_10653	3H	69.4
11_20746	6H	85.94
11_10654	0	0
11_20748	2H	61.49
11_10658	5H	139
11_10656	2H	145.69
11_20750	7H	61.67
11_10659	6H	59.25
11_20754	1H	101.99

11_10661	5H	42.41
11_20758	7H	28.16
11_20762	4H	109
11_10667	4H	54.73
11_10668	4H	50.22
11_10669	6H	2.84
11_20765	4H	95.77
11_10671	5H	50.53
11_20766	5H	38.78
11_20769	1H	92.43
11_20772	1H	142.55
11_10673	7H	84.3
11_10676	6H	32.05
11_20777	4H	29.76
11_20778	3H	87.8
11_20780	1H	108.52
11_10679	2H	66.2
11_20782	4H	54.94
11_20783	6H	100.68
11_20784	6H	80.06
11_10681	3H	172.82
11_10682	7H	0.63
11_20786	5H	183.14
11_10685	2H	72.99
11_10686	1H	77.92
11_10687	7H	139.9
11_20790	7H	49.82
11_10688	5H	28.98
11_20791	5H	145.9
11_20792	1H	92.43
11_20794	3H	28.02
11_20795	5H	95.52
11_20796	3H	67.86
11_20797	3H	5.36
11_20798	1H	48.99
11_10692	2H	73.35
11_20799	6H	59.25
11_20801	3H	66.62
11_10694	3H	173.43
11_10697	4H	135.58
11_10700	7H	80.3
11_10702	3H	169.95
11_20808	7H	102.97
11_20810	1H	46.5
11_10705	5H	125.09
11_20815	4H	85.07
11_20820	4H	73.03
11_10707	2H	131.69
11_20824	7H	109.44

11_10710	3H	39.57
11_20829	5H	160.9
11_20828	7H	81.78
11_10712	4H	132.78
11_20835	6H	60.65
11_20838	4H	107.14
11_20840	1H	140.69
11_20841	5H	41.45
11_20844	1H	111.81
11_20845	5H	33.55
11_10722	1H	126.61
11_10723	4H	93.08
11_10724	4H	91.42
11_20885	7H	75.52
11_20850	5H	90.07
11_20851	3H	148.79
11_20853	4H	55.58
11_20854	6H	59.25
11_20855	1H	41.74
11_20856	3H	67.86
11_10726	7H	51.23
11_10728	3H	72.21
11_20862	2H	67.08
11_10729	1H	116.24
11_20864	2H	32.35
11_20866	3H	63.94
11_20873	5H	21.14
11_10731	2H	124.98
11_10733	2H	56.64
11_10734	6H	109.05
11_20877	3H	83.8
11_20879	7H	81.78
11_10736	5H	171.58
11_20882	6H	3.46
11_20884	5H	132.32
11_20886	6H	1.64
11_20887	2H	72.99
11_10741	5H	155.45
11_20889	6H	86.54
11_20890	3H	65.25
11_20891	2H	61.49
11_20892	6H	79.17
11_10744	1H	23.62
11_20893	7H	81.78
11_20894	5H	1.26
11_20895	2H	149.27
11_20896	7H	88.06
11_20897	5H	177.9
11_10747	3H	104.15

11_10748	6H	133.9
11_10749	6H	59.25
11_10750	0	0
11_10751	4H	97.05
11_20904	6H	72.17
11_10753	3H	123.37
11_10754	3H	134.71
11_20906	4H	77.87
11_20908	1H	122.34
11_20909	1H	105.77
11_10755	5H	136.94
11_20911	7H	81.78
11_20912	1H	46.5
11_10756	4H	54.73
11_20915	1H	141.46
11_10757	1H	22.04
11_20920	3H	148.79
11_20921	1H	109.38
11_10760	1H	28.2
11_20924	4H	75.44
11_10764	1H	34.45
11_20931	3H	81.28
11_10767	3H	179.16
11_10768	1H	52.63
11_10770	2H	167.66
11_20934	5H	159.55
11_10771	5H	88.05
11_20936	6H	46.44
11_10772	7H	47.1
11_20939	4H	51.53
11_10773	7H	81.78
11_20943	2H	163.19
11_20944	3H	131.24
11_20946	6H	59.25
11_20947	2H	84.96
11_10778	5H	166.65
11_20952	3H	156.42
11_10780	2H	137.03
11_20956	1H	64.44
11_10781	6H	72.17
11_20958	5H	42.41
11_20959	1H	119.78
11_10783	5H	130.32
11_20960	2H	85.73
11_20961	5H	51.51
11_20962	7H	152.41
11_10785	4H	102.93
11_20968	3H	29.75
11_10787	2H	31.91

11_20969	6H	84.47
11_20970	3H	67.86
11_20972	6H	106.82
11_10789	1H	114.06
11_20974	4H	116.68
11_20975	7H	62.43
11_10791	2H	164.35
11_10793	4H	50.22
11_20982	0	0
11_20980	5H	33.55
11_20987	5H	38.78
11_20988	5H	158.28
11_10796	2H	63.55
11_10797	7H	135.86
11_10798	1H	59.29
11_10799	6H	35.32
11_20990	1H	80.47
11_20993	7H	29.7
11_20996	6H	104.5
11_20994	2H	160.46
11_20995	3H	69.95
11_20998	0	0
11_20999	3H	108.7
11_21000	1H	45.85
11_21001	5H	63.93
11_21005	2H	54.92
11_21008	3H	169.29
11_10805	5H	122.33
11_21010	4H	68.19
11_21012	5H	167.4
11_21011	5H	42.41
11_21014	6H	58.48
11_21015	2H	26
11_10809	4H	63.79
11_21018	5H	157.61
11_21025	6H	102.04
11_21027	3H	12.38
11_10814	1H	28.99
11_10813	3H	83.58
11_10815	6H	97
11_21030	6H	44.96
11_21032	6H	11.68
11_10817	6H	49.67
11_21035	4H	134.55
11_10818	2H	90.48
11_21038	1H	122.34
11_21041	5H	154.08
11_21040	5H	42.41
11_10819	5H	139

11_10820	5H	154.08
11_10821	3H	134.71
11_21050	7H	15.89
11_10823	2H	95.58
11_21053	1H	51.91
11_10825	3H	39.92
11_10826	2H	155.68
11_21061	5H	99.39
11_21062	3H	67.27
11_10829	4H	83.75
11_21067	1H	1.88
11_21069	6H	71.32
11_21070	4H	28
11_21071	4H	54.94
11_21072	1H	30.81
11_21073	4H	54.73
11_10830	1H	89.77
11_21077	5H	143.29
11_21079	7H	84.3
11_21083	3H	111.49
11_10834	5H	98.2
11_21087	4H	71.71
11_21088	2H	145.69
11_10837	2H	42.01
11_10838	7H	32.01
11_21093	3H	59.83
11_10839	3H	77.37
11_10840	5H	50.53
11_10841	7H	9.73
11_10842	3H	139
11_21099	2H	168.26
11_10843	7H	145.6
11_10846	4H	76.31
11_21104	7H	134.1
11_10848	6H	59.25
11_21109	3H	63.25
11_21121	5H	58.65
11_21110	2H	76.61
11_10851	7H	9.73
11_21111	4H	113.95
11_21112	6H	139.75
11_10853	7H	111.15
11_10854	1H	119.78
11_10855	5H	132.32
11_10856	5H	42.41
11_21120	3H	75.27
11_21122	4H	35.24
11_10861	7H	134.1
11_10863	3H	42.31

11_21125	2H	146.72
11_21126	1H	78.58
11_21129	3H	63.94
11_21133	5H	67.66
11_10867	3H	135.43
11_21134	1H	37.3
11_10869	5H	165.28
11_10870	5H	168.67
11_21136	2H	101.3
11_21138	5H	170.25
11_21140	1H	127.26
11_21142	0	0
11_21141	5H	167.9
11_21144	2H	78.54
11_21145	3H	42.96
11_21147	3H	67.27
11_21148	5H	50.53
11_21150	5H	83.17
11_21153	2H	43.38
11_21155	5H	182.53
11_21158	6H	57.83
11_21161	3H	122.7
11_21168	5H	98.2
11_21174	1H	8.96
11_21175	2H	108.58
11_10882	6H	44.96
11_21177	5H	125.09
11_21181	2H	155.68
11_21184	2H	124.98
11_10885	7H	138.83
11_21187	2H	28.15
11_21188	1H	42.42
11_10886	3H	13.78
11_21189	3H	52.17
11_21191	4H	70.77
11_21192	1H	89.77
11_21193	1H	42.42
11_21197	3H	63.25
11_10890	1H	76.92
11_21200	5H	51.51
11_10891	2H	29.75
11_21201	7H	98.95
11_21202	5H	7.84
11_21203	5H	121.67
11_10894	7H	2.13
11_10896	7H	150.44
11_21204	6H	8.74
11_21205	2H	82.98
11_21207	0	0

11_21209	7H	130.64
11_21210	4H	138.7
11_10900	2H	114.34
11_21212	3H	120.73
11_21215	5H	42.41
11_21216	6H	59.25
11_21217	1H	48.29
11_21220	2H	136.33
11_10901	5H	152.93
11_10902	5H	155.45
11_21223	7H	144.96
11_21224	6H	86.96
11_21225	6H	72.17
11_21226	1H	9.37
11_10903	1H	133.47
11_21228	4H	3.8
11_21229	7H	128.72
11_21238	2H	125.97
11_21239	5H	60.21
11_21241	5H	131.64
11_10911	1H	121.8
11_21242	2H	94.25
11_10913	5H	42.41
11_10914	4H	65.28
11_21244	5H	51.51
11_10916	2H	134.35
11_10918	3H	132.62
11_10919	2H	42.01
11_10920	7H	26.14
11_21247	5H	125.09
11_21250	2H	159.62
11_21251	2H	82.44
11_21253	5H	36.72
11_21254	4H	56.22
11_21256	6H	80.86
11_10924	7H	80.64
11_10925	3H	66.62
11_10926	3H	67.86
11_21258	2H	80.36
11_21260	5H	49.16
11_21261	2H	27.5
11_21265	2H	27.5
11_21267	3H	174.45
11_21271	6H	118.15
11_10933	1H	45.2
11_21270	7H	62.64
11_21272	3H	157.77
11_21274	2H	154.39
11_21275	5H	57.89

11_10939	6H	37.29
11_21277	3H	124.04
11_10942	4H	55.15
11_21280	7H	141.15
11_21281	6H	47.1
11_10943	2H	17.24
11_21286	2H	66.2
11_10946	4H	59.06
11_10947	2H	67.08
11_21289	5H	136.94
11_21293	6H	72.17
11_21294	3H	100.29
11_21296	4H	71.71
11_21297	5H	145.9
11_21298	6H	70.5
11_21299	2H	163.19
11_21302	7H	81.78
11_10954	6H	59.25
11_10956	7H	27.69
11_21304	2H	34.24
11_10957	1H	41.74
11_21305	3H	80.49
11_21307	7H	1.71
11_21308	5H	41.45
11_21309	5H	64.77
11_21310	6H	65.83
11_21312	1H	45.2
11_21314	5H	96.12
11_21315	2H	138.04
11_21318	5H	44.23
11_21321	5H	97.49
11_10965	7H	24.15
11_21324	5H	25.42
11_10966	3H	65.25
11_21325	5H	123.12
11_21326	7H	42.26
11_10971	7H	0
11_10974	5H	21.68
11_21333	1H	52.63
11_10978	6H	106.15
11_21339	6H	65.29
11_21340	2H	116.5
11_21344	5H	53.84
11_10983	7H	73.54
11_21346	2H	161.3
11_21348	3H	98.66
11_21350	5H	42.41
11_21353	4H	86.01
11_10988	2H	124.98

11_21354	1H	1.68
11_10989	2H	125.97
11_21355	5H	149.94
11_21357	1H	44.59
11_21358	3H	93.81
11_21359	4H	20.44
11_21361	1H	48.99
11_21363	7H	145.6
11_21366	2H	27.5
11_21370	2H	143.18
11_10994	6H	34.66
11_10995	5H	42.41
11_21373	1H	96.95
11_10996	2H	58.5
11_10997	2H	62.85
11_21374	4H	30.37
11_10999	7H	157.44
11_21377	2H	9.12
11_21381	3H	112.15
11_11002	3H	48.02
11_21384	1H	138.86
11_21385	4H	26.2
11_21390	5H	106.36
11_21391	5H	33.55
11_21392	1H	114.06
11_11004	4H	86.66
11_21397	4H	35.24
11_21398	3H	11.01
11_21399	2H	72.99
11_21401	5H	40.14
11_21402	3H	56.54
11_21405	3H	132.62
11_21406	2H	142.67
11_11012	7H	149.31
11_21409	7H	89.12
11_21414	0	0
11_11014	7H	54.32
11_11015	2H	60.89
11_11016	3H	71.26
11_11019	4H	146.48
11_21421	5H	91.98
11_21419	7H	0
11_21425	6H	133.9
11_21427	3H	150.11
11_21428	3H	143.33
11_21431	1H	62.6
11_21435	3H	66.62
11_21436	2H	164.35
11_21437	7H	11.65

11_11023	2H	158.6
11_21438	3H	108.7
11_21440	2H	140.69
11_11024	5H	121.67
11_21443	7H	0.84
11_21445	5H	74.83
11_21447	5H	41.45
11_21448	7H	98.95
11_11028	7H	65.98
11_11031	7H	5.96
11_21452	5H	155.23
11_21453	2H	168.26
11_11038	1H	132.16
11_11042	4H	56.22
11_11043	2H	125.97
11_11046	2H	66.2
11_11048	5H	24.76
11_21459	2H	143.18
11_11049	1H	59.29
11_11054	2H	53.18
11_11058	2H	102.38
11_11066	4H	134.55
11_11067	6H	62.71
11_11071	5H	136.94
11_11072	2H	77.76
11_11075	1H	75.48
11_21467	6H	128.41
11_11080	5H	130.98
11_21469	6H	76.03
11_11086	3H	64.6
11_11090	5H	125.09
11_21472	3H	66.62
11_11094	2H	119.72
11_21473	6H	57.18
11_11098	7H	64.98
11_11099	3H	67.27
11_11105	1H	138.86
11_11111	6H	139.09
11_11114	4H	59.8
11_11118	2H	128.87
11_11122	7H	79.79
11_11124	3H	67.86
11_11125	3H	66.62
11_11128	5H	47.47
11_11132	7H	0.63
11_11136	4H	24.71
11_21481	4H	62.81
11_11141	3H	137.74
11_11147	6H	95.7

11_11153	6H	59.91
11_11159	5H	52.86
11_11162	1H	45.2
11_11178	2H	68.07
11_11179	7H	4.5
11_11180	4H	48.77
11_11185	5H	154.08
11_11186	4H	144.4
11_11187	6H	131.64
11_11191	3H	75.27
11_11196	3H	138.39
11_11198	5H	40.14
11_11199	4H	23.96
11_11200	5H	109.48
11_11205	6H	53.54
11_11206	2H	69.05
11_11207	4H	72.36
11_11211	2H	67.08
11_11213	4H	95.22
11_11216	5H	162.98
11_11219	7H	82.2
11_11221	5H	51.51
11_11223	1H	1.24
11_11224	4H	78.5
11_11227	2H	149.27
11_11229	4H	78.08
11_11236	2H	127.74
11_11239	7H	85.28
11_11240	5H	51.51
11_11241	3H	84.22
11_11243	7H	121.36
11_11244	4H	61.56
11_11246	6H	93.44
11_21491	7H	41.51
11_11249	5H	60.21
11_21493	3H	115.21
11_11256	1H	49.65
11_11258	3H	63.94
11_21494	7H	81.78
11_11260	5H	43.15
11_11262	2H	156.77
11_21495	3H	114.36
11_11273	5H	105.69
11_11275	7H	151.76
11_11276	0	0
11_11277	1H	100.4
11_11281	5H	54.77
11_11283	3H	66.62
11_11287	1H	43.08

11_11290	5H	83.17
11_11292	4H	109.65
11_11294	6H	105.05
11_11302	2H	56.64
11_11307	2H	109.29
11_11312	6H	59.25
11_11314	3H	83.16
11_11323	2H	118.39
11_11329	6H	78.52
11_11330	3H	129.83
11_11332	4H	56.22
11_11336	1H	42.42
11_11341	5H	108.28
11_21502	3H	76.43
11_11345	4H	5.23
11_11346	2H	114.34
11_11348	7H	64.98
11_11349	6H	80.06
11_11350	5H	93.66
11_21504	4H	80.73
11_11354	2H	68.07
11_11355	5H	77.75
11_11361	0	0
11_11365	2H	138.04
11_11367	1H	64.44
11_11375	5H	123.12
11_21507	0	0
11_11380	2H	160.46
11_11381	5H	8.25
11_11384	2H	69.79
11_21509	6H	64.65
11_21511	3H	71.26
11_11391	3H	77.37
11_11394	3H	81.28
11_11398	4H	97.05
11_11400	2H	58.5
11_21514	5H	147.49
11_11405	4H	53.28
11_11406	6H	6.54
11_11431	4H	76.31
11_21516	7H	0.21
11_11432	5H	38.78
11_11435	2H	89.68
11_11436	3H	163.49
11_11440	7H	146.27
11_11441	5H	143.29
11_11445	7H	85.28
11_21521	6H	2.24
11_11448	5H	131.64

11_11453	3H	8.56
11_11456	5H	120.35
11_11458	6H	88.88
11_11459	6H	80.86
11_11461	7H	83.42
11_11464	5H	158.28
11_21523	3H	169.95
11_11469	5H	42.41
11_11470	4H	111.13
11_11473	5H	89.37
11_11478	1H	42.42
11_11479	6H	15.16
11_11480	2H	118.39
11_21528	7H	38.99
11_11481	1H	128.04
11_11483	6H	70.5
11_11488	6H	133.9
11_11489	0	0
11_11490	5H	150.29
11_11495	7H	6.79
11_11497	5H	151.35
11_11500	4H	89.36
11_11501	3H	67.86
11_11502	3H	67.86
11_11503	3H	125.11
11_11505	2H	51.87
11_11507	5H	118.16
11_11509	1H	138.86
11_11513	4H	80.73
11_21533	3H	47.37
11_11516	3H	174.99
11_11521	7H	127.74
11_11522	2H	58.15
11_21536	5H	46.38
11_11528	1H	122.34
11_11530	3H	66.62
11_11533	2H	100.05
11_11534	6H	122.74
12_10014	3H	173.43
12_10016	5H	149.94
12_10022	4H	95.27
12_10032	0	0
12_10034	5H	51.51
12_10053	4H	76.31
12_10063	4H	44.99
12_10071	6H	130.38
12_10077	5H	95.11
12_10088	4H	63.79
12_10089	7H	91.12

12_10099	2H	68.07
12_10100	3H	125.11
12_10103	3H	3.97
12_10105	0	0
12_10122	3H	143.33
12_10125	7H	83.42
12_10149	0	0
12_10151	0	0
12_10154	2H	69.05
12_10155	3H	67.86
12_10159	1H	42.42
12_10166	1H	71.81
12_10170	4H	88.7
12_10171	4H	43.72
12_10179	0	0
12_10181	2H	160.46
12_10188	3H	137.74
12_10195	4H	56.22
12_10198	1H	51.2
12_10199	6H	49.67
12_10201	1H	58.59
12_10203	5H	59.72
12_10205	3H	141.22
12_10207	1H	128.04
12_10210	0	0
12_10218	7H	32.13
12_10219	0	0
12_10228	5H	121.67
12_10230	2H	7.92
12_10235	1H	40.4
12_10241	7H	112.99
12_10257	0	0
12_10264	5H	47.04
12_10267	7H	61.67
12_10268	7H	81.78
12_10271	4H	102.93
12_10273	5H	157.61
12_10278	6H	60.65
12_10284	0	0
12_10300	1H	42.42
12_10306	0	0
12_10308	0	0
12_10313	0	0
12_10314	1H	38.36
12_10322	5H	189.22
12_10333	5H	147.49
12_10337	0	0
12_10344	3H	114.36
12_10345	6H	60.65

12_10347	4H	43.72
12_10348	0	0
12_10362	7H	110.11
12_10367	0	0
12_10368	7H	38.57
12_10371	4H	44.33
12_10374	0	0
12_10375	7H	4.5
12_10378	7H	162.03
12_10392	6H	72.17
12_10393	0	0
12_10395	4H	30.37
12_10403	7H	55.64
12_10406	7H	7.33
12_10408	5H	90.07
12_10410	1H	4.71
12_10420	1H	0.54
12_10426	4H	62.81
12_10430	0	0
12_10452	3H	85.4
12_10459	7H	81.78
12_10472	2H	147.37
12_10474	2H	67.63
12_10485	2H	63.55
12_10486	2H	49.5
12_10487	2H	159.62
12_10489	0	0
12_10491	0	0
12_10493	0	0
12_10497	6H	56.06
12_10499	5H	28.98
12_10502	2H	10.86
12_10505	3H	137.74
12_10508	5H	47.04
12_10535	1H	91.73
12_10543	7H	121.36
12_10545	2H	77.76
12_10554	6H	23.62
12_10562	4H	26.2
12_10564	0	0
12_10571	3H	18.21
12_10579	2H	149.93
12_10581	7H	82.41
12_10583	3H	102.66
12_10591	6H	59.25
12_10596	6H	78.52
12_10605	7H	58.53
12_10609	3H	87.8
12_10613	0	0

12_10623	0	0
12_10629	3H	172.82
12_10633	5H	69.29
12_10634	5H	68.21
12_10636	1H	5.41
12_10637	4H	62.81
12_10640	2H	72.03
12_10649	2H	108.58
12_10650	2H	84.96
12_10652	7H	112.99
12_10657	7H	61.67
12_10662	3H	114.36
12_10666	4H	109.65
12_10670	4H	94.07
12_10674	5H	80.18
12_10677	7H	142.46
12_10678	3H	71.26
12_10680	3H	128.01
12_10689	0	0
12_10693	1H	130.42
12_10698	7H	81.78
12_10704	6H	117.51
12_10717	2H	82.98
12_10718	2H	3.1
12_10725	5H	52.86
12_10732	5H	187.2
12_10735	0	0
12_10739	2H	134.29
12_10746	1H	141.46
12_10752	5H	103.74
12_10758	6H	65.83
12_10766	2H	158.18
12_10769	5H	163.72
12_10803	6H	63.71
12_10808	1H	129.71
12_10810	4H	37.88
12_10811	6H	49.67
12_10824	4H	102.93
12_10836	0	0
12_10844	5H	97.49
12_10847	2H	34.24
12_10857	5H	188.7
12_10859	2H	93.26
12_10860	4H	37.88
12_10864	5H	35.39
12_10878	0	0
12_10880	0	0
12_10887	0	0
12_10888	7H	157.85

12_10897	0	0
12_10899	5H	51.51
12_10904	5H	147.49
12_10905	1H	116.16
12_10915	2H	154.59
12_10923	5H	40.14
12_10927	2H	61.49
12_10930	5H	83.17
12_10937	2H	166.6
12_10938	1H	44.59
12_10948	2H	63.55
12_10950	2H	159.62
12_10953	5H	63.93
12_10959	7H	52.52
12_10968	3H	38.91
12_10969	2H	102.38
12_10970	2H	0
12_10973	7H	137.31
12_10979	7H	36.92
12_10981	0	0
12_10982	7H	83.42
12_11011	1H	6.98
12_11030	2H	6.09
12_11035	7H	6.71
12_11039	0	0
12_11044	7H	97.65
12_11047	3H	150.11
12_11051	7H	105.73
12_11055	7H	85.28
12_11062	1H	64.44
12_11063	4H	56.22
12_11069	3H	53.2
12_11077	0	0
12_11078	0	0
12_11084	3H	75.27
12_11091	7H	85.28
12_11095	0	0
12_11096	2H	79.92
12_11103	7H	70.21
12_11104	6H	56.06
12_11106	5H	83.17
12_11107	1H	47.21
12_11108	4H	65.28
12_11119	2H	10.86
12_11121	2H	76.61
12_11131	2H	57.29
12_11137	0	0
12_11138	3H	98.05
12_11139	4H	111.81

12_11140	6H	59.91
12_11144	1H	89.77
12_11146	7H	81.78
12_11150	3H	75.27
12_11151	5H	51.51
12_11154	3H	147.57
12_11155	2H	66.2
12_11164	0	0
12_11171	6H	53.54
12_11173	1H	103.99
12_11175	4H	26.71
12_11177	0	0
12_11181	6H	62.71
12_11183	4H	111.81
12_11184	7H	118.44
12_11190	4H	56.22
12_11192	5H	186.66
12_11194	4H	117.61
12_11208	0	0
12_11217	1H	48.29
12_11235	4H	133.52
12_11237	3H	29.09
12_11245	5H	113.51
12_11253	6H	62.71
12_11254	0	0
12_11255	0	0
12_11267	1H	76.22
12_11269	0	0
12_11271	1H	136.7
12_11274	0	0
12_11278	2H	73.89
12_11279	7H	129.32
12_11285	2H	108.58
12_11288	2H	67.08
12_11295	3H	67.27
12_11297	3H	156.46
12_11300	4H	12.34
12_11301	1H	41.74
12_11309	7H	151.1
12_11310	3H	13.13
12_11311	1H	3.21
12_11315	0	0
12_11316	2H	73.89
12_11318	5H	34.69
12_11321	6H	65.83
12_11322	0	0
12_11324	2H	72.99
12_11325	5H	157.61
12_11353	6H	56.06

12_11357	1H	30.15
12_11368	2H	160.46
12_11377	7H	84.3
12_11382	4H	42.22
12_11383	0	0
12_11385	5H	52.86
12_11386	0	0
12_11399	5H	42.41
12_11408	0	0
12_11409	1H	117.24
12_11413	5H	187.2
12_11414	3H	34.15
12_11429	3H	71.26
12_11433	7H	7.33
12_11434	3H	6.31
12_11437	7H	94.34
12_11443	1H	125.29
12_11444	1H	54.54
12_11449	2H	86.58
12_11450	5H	169.14
12_11452	2H	33.82
12_11454	3H	90.1
12_11455	6H	44.96
12_11462	5H	42.41
12_11463	1H	88.88
12_11466	2H	114.34
12_11468	0	0
12_11472	5H	123.98
12_11475	6H	70.5
12_11485	4H	10.77
12_11487	6H	59.25
12_11492	0	0
12_11494	6H	111.74
12_11498	1H	34.45
12_11499	7H	85.28
12_11510	3H	162.17
12_11511	3H	61.94
12_11512	5H	51.51
12_11517	3H	93.81
12_11525	7H	61.67
12_11526	4H	90.1
12_11529	7H	85.28
12_11535	5H	121.67
12_11536	7H	81.78
12_20006	0	0
12_20016	7H	0
12_20027	2H	155.68
12_20031	7H	48.06
12_20045	5H	119.65

12_20059	5H	45.64
12_20079	7H	156.13
12_20090	3H	3.97
12_20108	3H	69.4
12_20142	6H	59.25
12_20143	4H	86.01
12_20158	0	0
12_20183	2H	145.03
12_20187	1H	105.77
12_20196	2H	67.08
12_20201	7H	2.13
12_20217	7H	120.52
12_20227	7H	8.45
12_20234	0	0
12_20235	2H	61.49
12_20237	4H	130.81
12_20241	7H	142.46
12_20257	0	0
12_20274	4H	6.86
12_20278	5H	59.72
12_20285	0	0
12_20295	0	0
12_20297	5H	63.33
12_20323	0	0
12_20326	2H	42.01
12_20345	3H	180.12
12_20350	5H	61.88
12_20368	2H	30.42
12_20369	3H	138.39
12_20381	6H	56.06
12_20403	5H	94.4
12_20413	3H	135.43
12_20416	0	0
12_20421	3H	162.17
12_20424	0	0
12_20429	1H	143.2
12_20436	0	0
12_20448	6H	121.44
12_20489	2H	90.48
12_20505	3H	162.83
12_20574	3H	67.86
12_20591	3H	67.27
12_20593	2H	29.05
12_20611	7H	104.32
12_20613	1H	105.77
12_20632	0	0
12_20640	7H	148.28
12_20641	0	0
12_20649	0	0

12_20684	7H	109.99
12_20685	7H	94.34
12_20688	2H	55.46
12_20760	4H	138.7
12_20770	5H	35.88
12_20775	0	0
12_20793	2H	103.13
12_20818	5H	64.79
12_20825	0	0
12_20830	5H	142.54
12_20831	4H	56.22
12_20849	3H	92.73
12_20863	3H	73.3
12_20867	5H	167.4
12_20917	2H	61.49
12_20949	7H	142.46
12_20954	0	0
12_20981	5H	51.51
12_20985	0	0
12_20989	2H	130.38
12_21003	0	0
12_21009	5H	168.44
12_21019	0	0
12_21036	5H	57.04
12_21049	2H	31.17
12_21114	6H	60.65
12_21115	0	0
12_21117	4H	0
12_21131	1H	59.99
12_21137	4H	57.54
12_21157	0	0
12_21167	7H	75.52
12_21172	1H	121.56
12_21186	5H	74.83
12_21208	7H	116.28
12_21234	7H	68.89
12_21290	5H	167.4
12_21319	7H	82.41
12_21328	7H	151.76
12_21337	2H	67.08
12_21372	5H	39.48
12_21376	3H	159.54
12_21386	3H	154.44
12_21393	5H	177.9
12_21415	2H	3.84
12_21442	4H	56.22
12_21462	5H	143.29
12_21463	1H	57.27
12_21471	5H	125.09

12_21475	3H	67.86
12_21476	2H	74.55
12_21477	6H	125.99
12_21479	0	0
12_21482	6H	59.25
12_21492	7H	72.18
12_21497	5H	83.17
12_21500	3H	178.25
12_21522	0	0
12_21527	2H	114.34
12_21531	3H	158.05
12_30001	5H	4.15
12_30002	0	0
12_30003	0	0
12_30004	7H	81.78
12_30005	3H	77.37
12_30007	5H	60.21
12_30010	6H	18.89
12_30011	5H	52.86
12_30021	6H	58.48
12_30025	6H	127.01
12_30026	7H	94.34
12_30032	6H	56.06
12_30039	3H	67.27
12_30040	7H	26.14
12_30046	4H	105.83
12_30048	1H	143.2
12_30049	2H	118.39
12_30053	7H	81.78
12_30055	3H	178.99
12_30056	5H	107.19
12_30057	6H	131.64
12_30060	4H	62.81
12_30062	5H	147.49
12_30063	7H	26.14
12_30064	3H	50.19
12_30065	7H	36.26
12_30067	5H	123.78
12_30068	2H	67.08
12_30072	1H	87.43
12_30080	5H	60.21
12_30081	3H	136.92
12_30083	7H	29.7
12_30084	3H	134.71
12_30090	3H	108
12_30092	3H	143.33
12_30095	2H	117.17
12_30096	3H	135.43
12_30097	2H	139.29

12_30098	5H	94.4
12_30102	2H	172.92
12_30108	2H	72.99
12_30109	5H	42.41
12_30110	1H	45.2
12_30111	5H	51.51
12_30113	3H	17.66
12_30115	0	0
12_30116	0	0
12_30119	3H	116.24
12_30120	6H	56.06
12_30125	7H	67.56
12_30126	3H	67.86
12_30129	0	0
12_30130	3H	67.27
12_30133	6H	52.19
12_30135	3H	179.81
12_30137	3H	151.84
12_30138	4H	98.7
12_30140	4H	5.54
12_30141	7H	29.05
12_30142	4H	100.97
12_30143	7H	38.57
12_30144	6H	61.19
12_30148	6H	89.58
12_30149	7H	57.57
12_30150	4H	16.96
12_30151	6H	111.08
12_30153	0	0
12_30155	2H	10.86
12_30158	4H	109.65
12_30162	5H	157.61
12_30163	5H	0
12_30164	7H	118.44
12_30165	5H	155.45
12_30166	0	0
12_30167	5H	20.48
12_30168	7H	104.32
12_30169	5H	121.67
12_30170	3H	92.73
12_30178	2H	87.25
12_30181	7H	51.23
12_30183	5H	147.49
12_30186	0	0
12_30197	0	0
12_30199	7H	88.06
12_30200	2H	113.53
12_30203	0	0
12_30204	1H	89.77

12_30205	2H	82.44
12_30206	2H	67.08
12_30213	7H	83.42
12_30214	5H	44.89
12_30216	2H	107.92
12_30219	7H	29.05
12_30221	0	0
12_30222	0	0
12_30223	3H	129.83
12_30224	0	0
12_30226	4H	89.36
12_30230	6H	59.25
12_30231	1H	141.46
12_30232	4H	100.97
12_30236	6H	83.28
12_30237	4H	70.77
12_30239	4H	143.35
12_30242	7H	30.69
12_30243	1H	48.99
12_30244	7H	153.91
12_30250	3H	106.67
12_30251	2H	61.49
12_30259	2H	56.64
12_30260	0	0
12_30268	1H	36.64
12_30271	3H	169.95
12_30274	3H	133.36
12_30275	2H	72.99
12_30276	3H	125.11
12_30278	3H	95.14
12_30283	0	0
12_30284	3H	29.75
12_30285	0	0
12_30295	1H	69.08
12_30298	1H	73.13
12_30304	1H	59.29
12_30305	6H	59.91
12_30306	0	0
12_30310	2H	140.69
12_30314	5H	74.83
12_30316	6H	52.19
12_30318	3H	67.86
12_30319	6H	0
12_30323	2H	72.99
12_30325	3H	101.37
12_30329	7H	22.84
12_30331	4H	53.94
12_30335	7H	102.97
12_30336	1H	35.11

12_30337	5H	42.41
12_30342	3H	114.36
12_30343	1H	51.91
12_30344	7H	77.02
12_30346	6H	65.83
12_30348	1H	51.2
12_30350	1H	45.2
12_30351	0	0
12_30352	2H	156.77
12_30354	5H	41.45
12_30358	6H	38.66
12_30360	5H	187.2
12_30362	7H	114.96
12_30367	3H	149.45
12_30368	7H	118.52
12_30370	3H	164.15
12_30371	7H	47.1
12_30375	3H	125.11
12_30377	5H	121.01
12_30378	2H	171.6
12_30379	2H	48.8
12_30382	5H	188.04
12_30385	4H	118.31
12_30388	0	0
12_30389	7H	81.78
12_30390	4H	93.08
12_30394	4H	26.2
12_30396	2H	151.92
12_30399	3H	86.57
12_30400	5H	143.94
12_30402	2H	10.86
12_30403	1H	128.04
12_30404	1H	42.42
12_30406	1H	45.2
12_30408	5H	42.41
12_30409	0	0
12_30410	5H	31.2
12_30411	7H	81.78
12_30414	6H	137.41
12_30420	2H	40.69
12_30423	3H	120.73
12_30424	0	0
12_30425	4H	139.97
12_30427	4H	59.8
12_30430	6H	56.06
12_30431	3H	35.95
12_30432	2H	44.04
12_30438	1H	42.42
12_30441	6H	58.48

12_30444	0	0
12_30445	7H	81.78
12_30449	7H	81.78
12_30450	4H	56.88
12_30454	5H	41.45
12_30455	4H	77
12_30456	5H	107.19
12_30459	2H	131.03
12_30467	3H	53.86
12_30473	6H	56.06
12_30474	3H	59.83
12_30475	7H	82.41
12_30476	4H	139.97
12_30477	0	0
12_30488	4H	56.22
12_30491	2H	49.5
12_30492	7H	81.78
12_30494	5H	171.58
12_30496	7H	79.08
12_30498	1H	42.42
12_30502	0	0
12_30503	0	0
12_30504	5H	173.5
12_30505	1H	73.78
12_30506	7H	83.42
12_30508	6H	59.25
12_30509	1H	107.09
12_30510	6H	52.85
12_30514	2H	68.07
12_30516	6H	46.44
12_30517	0	0
12_30521	6H	41.55
12_30522	1H	47.21
12_30524	5H	116.66
12_30525	4H	56.22
12_30528	7H	42.26
12_30530	7H	20.29
12_30532	1H	105.77
12_30533	5H	86.74
12_30538	5H	50.53
12_30543	5H	1.91
12_30544	7H	81.78
12_30545	7H	46.44
12_30546	1H	94.15
12_30550	7H	81.78
12_30554	4H	102.93
12_30556	5H	139
12_30557	2H	62.85
12_30561	2H	68.07

12_30562	1H	42.42
12_30563	7H	81.78
12_30564	4H	53.28
12_30565	7H	83.42
12_30566	5H	162.68
12_30567	6H	59.25
12_30571	3H	32.92
12_30573	6H	88.88
12_30574	7H	82.41
12_30575	5H	42.41
12_30576	7H	52.52
12_30577	5H	177.9
12_30580	5H	143.94
12_30581	7H	79.08
12_30582	2H	68.07
12_30583	3H	58.51
12_30584	4H	105.17
12_30588	1H	12.94
12_30589	7H	81.78
12_30590	5H	121.67
12_30591	5H	6.36
12_30593	7H	146.93
12_30594	0	0
12_30598	2H	135.68
12_30600	7H	81.78
12_30603	0	0
12_30604	2H	55.46
12_30605	4H	56.22
12_30609	3H	52.17
12_30611	5H	121.67
12_30616	3H	78.25
12_30618	3H	64.6
12_30619	5H	108.28
12_30620	4H	76.31
12_30621	7H	81.78
12_30622	0	0
12_30626	6H	103.32
12_30631	2H	12.07
12_30634	2H	65.53
12_30635	5H	135.62
12_30637	6H	72.17
12_30640	3H	108.7
12_30642	5H	157.61
12_30644	5H	50.53
12_30645	7H	84.3
12_30646	0	0
12_30651	6H	7.87
12_30653	0	0
12_30654	5H	38.78

12_30655	0	0
12_30656	5H	169.72
12_30657	2H	42.01
12_30658	6H	52.19
12_30663	3H	100.29
12_30666	5H	162.03
12_30668	5H	130.98
12_30672	0	0
12_30674	2H	85.52
12_30677	3H	93.81
12_30680	3H	61.94
12_30683	1H	40.4
12_30685	0	0
12_30690	2H	145.69
12_30693	4H	79.47
12_30694	1H	45.2
12_30696	2H	89.68
12_30697	6H	32.27
12_30698	6H	98.68
12_30700	5H	50.53
12_30703	2H	50.99
12_30705	5H	103.01
12_30707	5H	36.72
12_30709	5H	42.41
12_30710	1H	49.65
12_30714	5H	13.83
12_30715	1H	3.21
12_30716	0	0
12_30717	5H	42.41
12_30718	4H	102.26
12_30721	3H	55.22
12_30723	7H	14.39
12_30724	2H	72.99
12_30728	5H	42.41
12_30729	5H	41.45
12_30734	6H	119.56
12_30736	3H	173.43
12_30737	3H	59.83
12_30742	1H	74.81
12_30743	3H	87.8
12_30745	5H	55.44
12_30748	0	0
12_30750	1H	45.2
12_30752	7H	46.44
12_30754	3H	84.22
12_30755	4H	77.66
12_30760	7H	81.78
12_30761	7H	141.15
12_30762	1H	42.42

12_30765	6H	59.25
12_30768	5H	42.41
12_30772	2H	66.2
12_30777	4H	53.94
12_30779	0	0
12_30780	7H	24.81
12_30781	2H	9.12
12_30782	6H	53.54
12_30783	6H	44.96
12_30786	1H	47.21
12_30788	3H	78.25
12_30792	5H	42.41
12_30793	0	0
12_30794	7H	81.78
12_30795	5H	148.58
12_30796	1H	40.4
12_30797	7H	116.94
12_30799	3H	63.94
12_30802	6H	58.48
12_30804	6H	65.83
12_30806	7H	99.94
12_30809	3H	67.86
12_30818	0	0
12_30819	0	0
12_30820	1H	4.71
12_30821	1H	54.54
12_30822	0	0
12_30823	2H	164.35
12_30824	4H	146.48
12_30825	4H	146.48
12_30826	7H	162.03
12_30827	0	0
12_30828	2H	67.08
12_30829	3H	73.3
12_30830	5H	149.27
12_30831	7H	135.86
12_30832	7H	79.08
12_30833	5H	140.14
12_30834	5H	88.05
12_30835	7H	81.78
12_30836	7H	5.43
12_30837	6H	59.25
12_30838	5H	35.39
12_30839	4H	62.81
12_30840	0	0
12_30842	6H	20.62
12_30843	6H	24.91
12_30845	0	0
12_30847	5H	98.2

12_30850	5H	98.2
12_30851	7H	9.73
12_30852	5H	98.2
12_30853	2H	66.2
12_30854	5H	98.2
12_30855	5H	96.12
12_30857	6H	61.19
12_30858	0	0
12_30859	0	0
12_30860	3H	145.84
12_30863	4H	35.24
12_30865	4H	35.24
12_30866	4H	56.88
12_30867	0	0
12_30871	2H	25.33
12_30872	2H	25.33
12_30873	4H	142.6
12_30877	0	0
12_30878	4H	56.22
12_30879	7H	55.64
12_30880	7H	54.99
12_30893	7H	31.35
12_30894	7H	31.35
12_30895	7H	31.35
12_30896	2H	99.39
12_30904	4H	77.66
12_30905	4H	77.66
12_30907	4H	30.37
12_30908	0	0
12_30910	3H	14
12_30913	3H	48.88
12_30914	2H	156.77
12_30917	0	0
12_30919	1H	11.35
12_30921	3H	163.49
12_30922	3H	60.58
12_30923	3H	60.58
12_30926	0	0
12_30927	3H	127.26
12_30929	5H	132.32
12_30930	5H	132.32
12_30931	6H	104.5
12_30933	1H	6.98
12_30934	1H	143.2
12_30935	0	0
12_30939	0	0
12_30940	6H	88.88
12_30942	2H	147.37
12_30944	0	0

12_30945	0	0
12_30948	1H	15.91
12_30949	0	0
12_30950	1H	11.35
12_30953	3H	40.99
12_30956	6H	142.2
12_30957	0	0
12_30958	5H	189.22
12_30959	7H	0
12_30962	0	0
12_30963	3H	137.74
12_30965	0	0
12_30967	0	0
12_30969	1H	0.31
12_30972	0	0
12_30973	3H	141.26
12_30974	7H	157.44
12_30975	5H	4.15
12_30976	5H	1.91
12_30977	5H	4.15
12_30979	5H	4.15
12_30980	5H	1.91
12_30981	0	0
12_30988	4H	111.81
12_30993	4H	49.43
12_30995	4H	62.81
12_30996	7H	94.34
12_30998	7H	85.28
12_30999	0	0
12_31000	7H	81.78
12_31004	6H	58.48
12_31005	6H	58.48
12_31006	6H	60.65
12_31008	3H	65.25
12_31009	3H	50.85
12_31010	3H	67.86
12_31011	3H	71.26
12_31012	3H	65.25
12_31014	3H	67.86
12_31015	3H	60.58
12_31016	3H	71.26
12_31017	3H	67.86
12_31018	3H	100.29
12_31020	2H	82.44
12_31021	2H	82.44
12_31023	5H	4.15
12_31032	5H	52.86
12_31033	5H	52.86
12_31035	5H	52.86

12_31041	0	0
12_31042	6H	112.39
12_31043	6H	112.39
12_31044	6H	112.39
12_31048	6H	112.39
12_31049	6H	112.39
12_31050	5H	143.29
12_31053	0	0
12_31054	0	0
12_31055	0	0
12_31059	0	0
12_31062	5H	51.51
12_31064	5H	51.51
12_31065	0	0
12_31066	0	0
12_31071	0	0
12_31081	1H	143.2
12_31086	0	0
12_31088	6H	83.81
12_31092	6H	52.85
12_31094	5H	11.45
12_31095	2H	133.04
12_31096	0	0
12_31099	0	0
12_31100	2H	142.67
12_31101	6H	80.86
12_31109	0	0
12_31111	6H	83.28
12_31113	0	0
12_31115	6H	112.39
12_31117	5H	46.38
12_31120	7H	75.52
12_31122	3H	59.83
12_31123	5H	189.89
12_31126	6H	131.64
12_31127	0	0
12_31134	1H	45.2
12_31138	4H	118.95
12_31139	4H	114.61
12_31144	1H	4.71
12_31148	4H	88.3
12_31151	0	0
12_31153	3H	80.49
12_31159	3H	40.99
12_31160	1H	74.81
12_31161	0	0
12_31163	1H	95.14
12_31164	4H	33.35
12_31165	5H	147.49

12_31166	7H	145.6
12_31167	0	0
12_31173	7H	5.43
12_31174	6H	60.65
12_31175	2H	68.07
12_31179	1H	61.39
12_31180	2H	168.26
12_31181	0	0
12_31182	5H	141.88
12_31183	5H	50.53
12_31186	4H	72.36
12_31187	6H	59.25
12_31189	2H	67.08
12_31194	0	0
12_31200	0	0
12_31202	0	0
12_31203	0	0
12_31205	2H	99.39
12_31206	5H	144.6
12_31207	0	0
12_31208	1H	42.42
12_31209	2H	156.77
12_31210	5H	187.2
12_31215	7H	81.78
12_31218	2H	67.08
12_31219	0	0
12_31220	3H	129.83
12_31221	5H	148.58
12_31222	0	0
12_31224	2H	4.59
12_31225	6H	105.05
12_31229	0	0
12_31230	0	0
12_31231	4H	86.01
12_31234	5H	141.88
12_31235	6H	103.32
12_31236	5H	103.01
12_31237	5H	132.32
12_31238	3H	135.43
12_31239	0	0
12_31240	0	0
12_31242	3H	82.62
12_31246	4H	92.41
12_31249	6H	59.25
12_31250	6H	80.06
12_31251	3H	147.57
12_31254	0	0
12_31256	2H	73.89
12_31257	5H	39.48

12_31259	5H	42.41
12_31261	7H	108.78
12_31262	3H	93.81
12_31264	2H	135.68
12_31267	0	0
12_31268	2H	147.37
12_31270	0	0
12_31271	5H	86.08
12_31274	6H	52.85
12_31276	1H	25.5
12_31277	6H	129.07
12_31279	0	0
12_31280	5H	52.86
12_31284	2H	15.98
12_31286	0	0
12_31288	2H	63.55
12_31289	6H	77.53
12_31292	5H	184.99
12_31293	2H	93.26
12_31294	7H	105.73
12_31297	4H	62.81
12_31298	3H	40.99
12_31299	3H	98.05
12_31305	7H	34.15
12_31308	6H	33.72
12_31312	5H	38.78
12_31313	4H	32.92
12_31315	0	0
12_31319	1H	100.4
12_31323	3H	83.58
12_31324	4H	0.75
12_31325	7H	142.46
12_31326	0	0
12_31327	0	0
12_31329	3H	125.86
12_31333	0	0
12_31340	5H	51.51
12_31346	3H	88.5
12_31350	7H	3.49
12_31351	7H	24.15
12_31352	5H	181.21
12_31353	6H	111.08
12_31356	3H	85.4
12_31357	0	0
12_31360	4H	56.22
12_31361	5H	80.18
12_31362	4H	83.75
12_31363	7H	116.94
12_31366	5H	136.94

12_31367	3H	106
12_31368	3H	67.86
12_31374	7H	126.83
12_31375	5H	157.61
12_31377	1H	128.04
12_31380	2H	85.52
12_31381	1H	44.59
12_31382	4H	53.94
12_31383	2H	84.96
12_31385	4H	77.66
12_31387	1H	129.71
12_31390	5H	41.45
12_31392	6H	127.76
12_31393	3H	71.26
12_31394	2H	85.52
12_31395	7H	99.94
12_31396	0	0
12_31398	2H	89.68
12_31401	1H	61.39
12_31406	2H	136.33
12_31408	0	0
12_31409	3H	9.56
12_31410	0	0
12_31411	0	0
12_31414	0	0
12_31417	5H	96.12
12_31418	7H	81.78
12_31422	4H	144.09
12_31423	5H	41.45
12_31424	2H	101.72
12_31427	5H	79.52
12_31428	3H	0
12_31431	0	0
12_31432	6H	107.49
12_31433	6H	52.85
12_31440	7H	105.07
12_31441	7H	64.98
12_31443	6H	59.25
12_31445	2H	90.48
12_31446	2H	0.66
12_31448	3H	6.31
12_31450	7H	7.87
12_31452	7H	69.55
12_31458	4H	12.34
12_31461	2H	155.68
12_31462	4H	62.81
12_31463	7H	61.67
12_31464	1H	64.44
12_31467	1H	40.4

12_31469	6H	136.66
12_31473	0	0
12_31477	5H	56.77
12_31479	0	0
12_31484	0	0
12_31485	6H	32.98
12_31486	4H	6.86
12_31490	0	0
12_31493	4H	77.66
12_31495	6H	122.14
12_31498	6H	136.66
12_31499	3H	131.24
12_31500	3H	160.19
12_31502	3H	67.27
12_31506	2H	166.6
12_31509	6H	59.25
12_31511	0	0
12_31512	5H	41.45
12_31513	0	0
12_31515	4H	76.31
12_31517	0	0
12_31520	5H	42.41
12_31521	0	0
12_31523	0	0
12_31525	3H	134.05
12_31527	2H	165.28
12_31529	3H	85.4
12_31535	7H	124.5
12_31536	4H	79.47

Table S6 SNPs with F_{ST} based on the Eastern and Western populations above 95th percentile genome-wide, including genetic position, GenBank ID, gene short name, in non-coding or coding region (1st, 2nd or 3rd positions), and silent or replacement information

SNP Name	Chr	cM	F_{ST}	GenBank ID	Gene Short Name	Position	Silent
11_10017	2H	81.31	0.32	AK353943	-	3	yes
11_10056	7H	33.49	0.41	AK364330	-	non-coding	yes
11_10116	5H	41.45	0.36	AK356434	-	3	yes
11_10243	2H	67.08	0.41	XM_003579657	LOC100838855	non-coding	yes
11_10253	3H	102.66	0.36	-	-	-	-
11_10424	4H	56.22	0.42	AK355297	-	3	yes
11_10498	2H	53.09	0.35	AK354555	-	1	yes
11_10536	5H	155.23	0.49	AK354787	-	3	yes
11_10614	4H	111.81	0.33	AK373775	-	3	yes
11_10644	1H	128.63	0.32	AK356375	-	non-coding	yes
11_10685	2H	72.99	0.47	AK362284	-	3	yes
11_10773	7H	81.78	0.36	AK373162	-	non-coding	yes
11_10813	3H	83.58	0.35	-	-	-	-
11_10956	7H	27.69	0.38	AK365588	-	3	yes
11_11024	5H	121.67	0.48	XM_003561753	LOC100839369	3	yes
11_11094	2H	119.72	0.38	-	-	-	-
11_11111	6H	139.09	0.39	AK366470	-	non-coding	yes
11_11243	7H	121.36	0.33	-	-	-	-
11_11432	5H	38.78	0.56	-	-	-	-
11_20029	6H	133.25	0.51	AK354118	-	1	no
11_20086	2H	110.93	0.32	AK358640	-	non-coding	yes
11_20133	1H	132.16	0.47	AK361091	-	non-coding	yes
11_20251	2H	68.56	0.36	AK370757	-	non-coding	yes
11_20283	5H	51.51	0.32	XM_003576993	LOC100831472	3	yes
11_20347	5H	121.67	0.48	AK359986	-	3	yes
11_20390	2H	72.99	0.46	FN179383	SBE2a	3	yes
11_20476	2H	67.08	0.33	BT087333	-	non-coding	yes
11_20482	4H	69.24	0.31	AK355084	-	3	yes
11_20498	2H	116.5	0.4	AK359654	-	3	yes
11_20577	6H	80.06	0.31	AK355031	-	non-coding	yes
11_20620	6H	78.52	0.31	AK366751	-	non-coding	yes
11_20669	2H	68.56	0.34	AK356298	-	non-coding	yes
11_20798	1H	48.99	0.32	AK354088	-	non-coding	yes
11_20904	6H	72.17	0.36	-	-	-	-
11_21302	7H	81.78	0.33	XM_003560861	LOC100844834	non-coding	yes

11_21399	2H	72.99	0.69	AK357878	-	1	no
11_21406	2H	142.67	0.34	AK370573	-	2	no
11_21447	5H	41.45	0.53	AK364513	-	2	no
11_21452	5H	155.23	0.53	AK372156	-	1	no
11_21502	3H	76.43	0.55	AK356987	-	3	yes
11_21504	4H	80.73	0.45	-	-	-	-
12_10053	4H	76.31	0.46	AK370758	-	3	yes
12_10071	6H	130.38	0.47	AK355485	-	non-coding	yes
12_10154	2H	69.05	0.71	AK355324	-	non-coding	yes
12_10170	4H	88.7	0.44	AK373474	-	non-coding	yes
12_10171	4H	43.72	0.34	AK368127	-	1	no
12_10199	6H	49.67	0.64	AK376992	-	non-coding	yes
12_10203	5H	59.72	0.52	AK356265	-	3	yes
12_10219	0	0	0.33	AY039003	Xantha-f	3	yes
12_10264	5H	47.04	0.52	WHTE1A	E1	non-coding	yes
12_10284	0	0	0.47	XM_003564030	LOC100843138	3	yes
12_10347	4H	43.72	0.5	AK362515	-	non-coding	yes
12_10472	2H	147.37	0.31	AK356296	-	non-coding	yes
12_10497	6H	56.06	0.48	XM_003570288	LOC100839823	1	no
12_10543	7H	121.36	0.4	DQ529207	Xantha-h	non-coding	yes
12_10591	6H	59.25	0.44	AK361815	-	1	no
12_10633	5H	69.29	0.32	AK371801	-	non-coding	yes
12_10634	5H	68.21	0.32	AK360310	-	3	yes
12_10689	0	0	0.38	AK369452	-	non-coding	yes
12_10810	4H	37.88	0.69	AK366265	-	non-coding	yes
12_11030	2H	6.09	0.31	AK364311	-	non-coding	yes
12_11107	1H	47.21	0.35	AK364999	-	non-coding	yes
12_11139	4H	111.81	0.38	Y14573	Mlo	3	yes
12_11151	5H	51.51	0.88	AK354730	-	3	yes
12_11184	7H	118.44	0.42	AK358083	-	non-coding	yes
12_11269	0	0	0.41	AK361959	-	1	no
12_11271	1H	136.7	0.4	-	-	-	-
12_11288	2H	67.08	0.52	AK366035	-	3	no
12_11310	3H	13.13	0.36	AK372013	-	3	yes
12_11316	2H	73.89	0.49	AK354712	-	non-coding	yes
12_11324	2H	72.99	0.91	AK356277	-	non-coding	yes
12_11377	7H	84.3	0.34	AK361273	-	3	yes
12_11408	0	0	0.41	-	-	-	-
12_20196	2H	67.08	0.55	AK362518	-	non-coding	yes

12_20235	2H	61.49	0.4	AK376421	-	non-coding	yes
12_20278	5H	59.72	0.52	AK356265	-	1	no
12_20326	2H	42.01	0.36	AK365480	-	non-coding	yes
12_20593	2H	29.05	0.44	AK367163	-	non-coding	yes
12_20760	4H	138.7	0.39	-	-	-	-
12_20981	5H	51.51	0.85	AK370568	-	3	yes
12_20989	2H	130.38	0.51	XM_003580352	LOC100845048	2	no
12_21003	0	0	0.76	-	-	-	-
12_21117	4H	0	0.32	AK359776	-	non-coding	yes
12_21234	7H	68.89	0.54	AK356095	-	2	no
12_21319	7H	82.41	0.37	XM_003573274	LOC100845308	non-coding	yes
12_30004	7H	81.78	0.34	-	-	-	-
12_30049	2H	118.39	0.41	-	-	-	-
12_30056	5H	107.19	0.35	AK356430	-	non-coding	yes
12_30068	2H	67.08	0.52	AK364966	-	3	yes
12_30164	7H	118.44	0.41	AK360970	-	non-coding	yes
12_30250	3H	106.67	0.65	AK356601	-	non-coding	yes
12_30441	6H	58.48	0.36	XM_003570517	LOC100832867	3	yes
12_30492	7H	81.78	0.41	EU961304	-	1	no
12_30504	5H	173.5	0.32	-	-	-	-
12_30563	7H	81.78	0.33	AJ582181	core3ft	2	no
12_30581	7H	79.08	0.45	AK356791	-	3	yes
12_30600	7H	81.78	0.54	AK362488	-	non-coding	yes
12_30616	3H	78.25	0.58	AK365474	-	non-coding	yes
12_30694	1H	45.2	0.6	-	-	-	-
12_30737	3H	59.83	0.39	AK361759	-	3	yes
12_30765	6H	59.25	0.44	AK356029	-	3	yes
12_30823	2H	164.35	0.32	AK375658	-	3	no
12_30850	5H	98.2	0.63	DQ480160	CBF4B	non-coding	yes
12_30956	6H	142.2	0.35	-	-	-	-
12_30988	4H	111.81	0.52	Y14573	Mlo	non-coding	yes
12_31017	3H	67.86	0.31	AK356796	-	non-coding	yes
12_31021	2H	82.44	0.51	AF112963	Cht2	non-coding	yes
12_31032	5H	52.86	0.33	AF326715	adh3	non-coding	yes
12_31035	5H	52.86	0.33	DQ195967	adh3	non-coding	yes
12_31043	6H	112.39	0.31	AY349220	Dhn5	3	yes
12_31062	5H	51.51	0.48	AK365941	-	3	yes
12_31064	5H	51.51	0.68	AK365941	-	3	yes
12_31100	2H	142.67	0.34	AK370573	-	2	no

12_31202	0	0	0.33	AK358464	-	non-coding	yes
12_31203	0	0	0.34	AK368679	-	non-coding	yes
12_31242	3H	82.62	0.36	AK369382	-	non-coding	yes
12_31246	4H	92.41	0.48	AK362249	-	non-coding	yes
12_31274	6H	52.85	0.38	AK357603	-	non-coding	yes
12_31385	4H	77.66	0.32	AK357832	-	non-coding	yes

Table S7 SNPs with Bayes Factor from environmental association analysis (Bayenv) above 95th percentile genome-wide, including genetic position, GenBank ID, gene short name, in non-coding or coding region (1st, 2nd or 3rd positions), and silent or replacement information. (A) PC1; (B) PC2

(A)

SNP Name	Chr	cM	Bayes Factor	GenBank ID	Gene Short Name	Position	Silent
11_10011	3H	67.86	9.22	AK371824	-	non-coding	yes
11_10090	4H	86.01	3.91	AK376851	-	non-coding	yes
11_10172	3H	82.62	5.04	AK366339	-	non-coding	yes
11_10184	3H	110.75	3.72	AK359211	-	3	yes
11_10208	4H	2.6	3.76	AK359180	-	3	yes
11_10232	7H	29.05	3.89	-	-	-	-
11_10247	4H	87.01	4.32	AJ585385	bcb	3	no
11_10253	3H	102.66	6.72	-	-	-	-
11_10446	2H	140.69	4.00	XM_003560174	LOC100837523	1	no
11_10641	5H	59.72	4.10	AK355250	-	3	yes
11_10653	3H	69.4	4.18	AK356876	-	2	no
11_10744	1H	23.62	4.80	AK353610	-	non-coding	yes
11_10813	3H	83.58	8.40	-	-	-	-
11_10818	2H	90.48	5.18	AK360545	-	non-coding	yes
11_10925	3H	66.62	3.83	AK363708	-	non-coding	yes
11_10989	2H	125.97	6.42	BLYCLDAB	cold-regulated	non-coding	yes
11_11111	6H	139.09	4.38	AK366470	-	non-coding	yes
11_11211	2H	67.08	4.06	-	-	-	-
11_11361	0	0	9.94	JF796668	CBF4	2	no
11_11497	5H	151.35	6.31	AK358288	-	3	yes
11_11521	7H	127.74	18.19	AK376764	-	3	yes
11_20012	4H	43.72	3.87	AK374823	-	non-coding	yes
11_20078	5H	155.66	5.77	AK376547	-	3	yes
11_20113	7H	49.61	5.40	AK355306	-	non-coding	yes
11_20133	1H	132.16	3.67	AK361091	-	non-coding	yes
11_20251	2H	68.56	5.27	AK370757	-	non-coding	yes
11_20306	5H	50.53	5.22	AK365203	-	non-coding	yes
11_20495	7H	18.73	3.96	AK359539	-	non-coding	yes
11_20537	6H	142.2	4.60	FJ853600	GSL2	non-coding	yes
11_20583	3H	66.62	4.92	AK365999	-	non-coding	yes
11_20620	6H	78.52	7.50	AK366751	-	non-coding	yes
11_20626	3H	109.43	3.65	AK366712	-	3	yes
11_20722	7H	13.7	5.24	AK377052	-	3	yes
11_20797	3H	5.36	3.96	AK375179	-	non-coding	yes

11_21030	6H	44.96	3.79	AK375921	-	2	no
11_21083	3H	111.49	4.11	AK372580	-	non-coding	yes
11_21174	1H	8.96	4.49	-	-	-	-
11_21191	4H	70.77	3.46	AK355907	-	non-coding	yes
11_21192	1H	89.77	5.99	AK357712	-	non-coding	yes
11_21193	1H	42.42	7.48	AK363338	-	non-coding	yes
11_21201	7H	98.95	5.56	-	-	-	-
11_21216	6H	59.25	5.25	AK376749	-	3	yes
11_21274	2H	154.39	3.85	AK354727	-	non-coding	yes
11_21296	4H	71.71	4.88	AK353992	-	non-coding	yes
11_21325	5H	123.12	3.84	AK362100	-	non-coding	yes
11_21399	2H	72.99	8.21	AK357878	-	1	no
11_21406	2H	142.67	3.68	AK370573	-	2	no
11_21459	2H	143.18	4.78	AM039897	ahh1	2	no
11_21502	3H	76.43	3.57	AK356987	-	3	yes
12_10122	3H	143.33	4.09	AK369540	-	non-coding	yes
12_10154	2H	69.05	14.43	AK355324	-	non-coding	yes
12_10199	6H	49.67	4.42	AK376992	-	non-coding	yes
12_10268	7H	81.78	5.32	XM_003574799	LOC100825330	3	yes
12_10535	1H	91.73	9.41	AK358527	-	3	yes
12_10678	3H	71.26	4.44	AK356724	-	non-coding	yes
12_10810	4H	37.88	3.49	AK366265	-	non-coding	yes
12_10824	4H	102.93	4.39	XM_003577507	LOC100843401	non-coding	yes
12_10938	1H	44.59	4.96	AK359548	-	1	no
12_11030	2H	6.09	4.18	AK364311	-	non-coding	yes
12_11051	7H	105.73	7.91	-	-	-	-
12_11151	5H	51.51	3.94	AK354730	-	3	yes
12_11154	3H	147.57	3.85	-	-	-	-
12_11271	1H	136.7	11.25	-	-	-	-
12_11288	2H	67.08	5.01	AK366035	-	3	no
12_11324	2H	72.99	41.73	AK356277	-	non-coding	yes
12_11386	0	0	5.77	-	-	-	-
12_11408	0	0	4.50	-	-	-	-
12_11455	6H	44.96	4.34	AK362081	-	non-coding	yes
12_11494	6H	111.74	3.53	AK368552	-	non-coding	yes
12_20235	2H	61.49	5.61	AK376421	-	non-coding	yes
12_20760	4H	138.7	8.63	-	-	-	-
12_20867	5H	167.4	3.85	AK359088	-	non-coding	yes
12_20981	5H	51.51	4.68	AK370568	-	3	yes

12_21117	4H	0	5.34	AK359776	-	non-coding	yes
12_21234	7H	68.89	3.83	AK356095	-	2	no
12_21319	7H	82.41	4.40	XM_003573274	LOC100845308	non-coding	yes
12_21337	2H	67.08	3.88	AK358015	-	3	yes
12_30004	7H	81.78	4.04	-	-	-	-
12_30060	4H	62.81	9.76	AK365085	-	non-coding	yes
12_30064	3H	50.19	3.70	AK367843	-	non-coding	yes
12_30068	2H	67.08	5.01	AK364966	-	3	yes
12_30080	5H	60.21	3.66	-	-	-	-
12_30135	3H	179.81	3.94	AK356358	-	non-coding	yes
12_30170	3H	92.73	3.50	AK353673	-	3	yes
12_30206	2H	67.08	3.88	-	-	-	-
12_30367	3H	149.45	15.26	AK353825	-	non-coding	yes
12_30404	1H	42.42	4.06	AK355367	-	3	yes
12_30475	7H	82.41	5.64	AK372209	-	non-coding	yes
12_30491	2H	49.5	3.90	-	-	-	-
12_30492	7H	81.78	5.94	EU961304	-	1	no
12_30504	5H	173.5	6.24	-	-	-	-
12_30554	4H	102.93	5.72	AK354013	-	2	no
12_30574	7H	82.41	4.96	AK370386	-	3	yes
12_30581	7H	79.08	18.82	AK356791	-	3	yes
12_30674	2H	85.52	4.34	AK369978	-	non-coding	yes
12_30715	1H	3.21	4.57	AK362579	-	non-coding	yes
12_30724	2H	72.99	3.51	AK368064	-	3	yes
12_30743	3H	87.8	4.49	AK364775	-	non-coding	yes
12_30745	5H	55.44	3.61	AK366468	-	3	yes
12_30779	0	0	5.07	AK376832	-	non-coding	yes
12_30781	2H	9.12	4.05	AK361785	-	non-coding	yes
12_30782	6H	53.54	6.44	-	-	-	-
12_30806	7H	99.94	4.96	-	-	-	-
12_31032	5H	52.86	3.59	AF326715	adh3	non-coding	yes
12_31066	0	0	4.85	AK372734	-	3	yes
12_31081	1H	143.2	21.95	AK356376	-	3	yes
12_31100	2H	142.67	3.68	AK370573	-	2	no
12_31218	2H	67.08	5.54	AK368932	-	2	no
12_31234	5H	141.88	9.78	AK358248	-	non-coding	yes
12_31357	0	0	6.10	-	-	-	-
12_31363	7H	116.94	3.45	AK375416	-	non-coding	yes
12_31381	1H	44.59	3.44	-	-	-	-

12_31383	2H	84.96	3.84	AK363632	-	non-coding	yes
12_31385	4H	77.66	4.68	AK357832	-	non-coding	yes
12_31392	6H	127.76	5.64	AK354269	-	non-coding	yes
12_31424	2H	101.72	3.69	AK375713	-	non-coding	yes
12_31486	4H	6.86	30.04	-	-	-	-

(B)

SNP Name	Chr	cM	Bayes Factor	GenBank ID	Gene Short Name	Position	Silent
11_10006	1H	76.92	4.55	AM502852	tub4	3	yes
11_10052	4H	76.31	3.08	AK370758	-	non-coding	yes
11_10129	6H	44.96	4.94	AK370146	-	3	yes
11_10172	3H	82.62	3.72	AK366339	-	non-coding	yes
11_10194	2H	67.08	2.86	AK359415	-	3	yes
11_10217	5H	149.94	3.16	AK354403	-	non-coding	yes
11_10240	5H	42.41	2.81	AK359441	-	non-coding	yes
11_10329	2H	168.26	3.71	AK362809	-	non-coding	yes
11_10424	4H	56.22	2.83	AK355297	-	3	yes
11_10522	1H	101.34	6.60	AK360842	-	1	yes
11_10580	5H	29.9	18.54	AK372891	-	non-coding	yes
11_10641	5H	59.72	5.49	AK355250	-	3	yes
11_10653	3H	69.4	2.96	AK356876	-	2	no
11_10767	3H	179.16	4.15	-	-	-	-
11_10780	2H	137.03	6.67	AK359167	-	3	yes
11_10919	2H	42.01	2.92	AK368475	-	non-coding	yes
11_11019	4H	146.48	2.85	FN179393	BAM1	2	no
11_11111	6H	139.09	4.09	AK366470	-	non-coding	yes
11_11147	6H	95.7	2.92	GU258512	-	non-coding	yes
11_11219	7H	82.2	5.81	AK368527	-	2	no
11_11406	6H	6.54	5.20	AF166121	Big1	3	yes
11_11521	7H	127.74	4.21	AK376764	-	3	yes
11_20114	4H	44.99	3.66	AK360127	-	3	yes
11_20129	5H	42.41	2.90	AK362176	-	non-coding	yes
11_20179	5H	42.41	2.85	AK357883	-	non-coding	yes
11_20260	1H	42.42	7.95	AK355367	-	3	yes
11_20306	5H	50.53	4.87	AK365203	-	non-coding	yes
11_20332	5H	49.16	3.04	WHTE1A	E1	non-coding	yes
11_20347	5H	121.67	4.55	AK359986	-	3	yes
11_20355	6H	120.69	3.68	AK353922	-	non-coding	yes

11_20383	1H	134.96	5.76	XM_003567791	LOC100830416	3	yes
11_20390	2H	72.99	10.41	FN179383	SBE2a	3	yes
11_20438	2H	69.05	4.78	AK364463	-	non-coding	yes
11_20485	7H	91.67	4.67	-	-	-	-
11_20498	2H	116.5	2.96	AK359654	-	3	yes
11_20527	3H	142.17	5.48	XM_003572099	LOC100831350	3	yes
11_20620	6H	78.52	12.93	AK366751	-	non-coding	yes
11_20709	6H	74.65	2.86	AK363507	-	3	yes
11_20736	5H	72.68	3.59	AK372237	-	3	yes
11_20797	3H	5.36	4.23	AK375179	-	non-coding	yes
11_20889	6H	86.54	3.18	XM_002454244	-	non-coding	yes
11_20924	4H	75.44	5.18	AK376504	-	1	no
11_20958	5H	42.41	2.90	AK372310	-	3	yes
11_20996	6H	104.5	5.87	AK360096	-	3	yes
11_21000	1H	45.85	6.15	AK370060	-	3	yes
11_21040	5H	42.41	2.81	AK370183	-	non-coding	yes
11_21181	2H	155.68	6.75	AK354575	-	3	yes
11_21193	1H	42.42	7.31	AK363338	-	non-coding	yes
11_21201	7H	98.95	10.97	-	-	-	-
11_21399	2H	72.99	8.83	AK357878	-	1	no
11_21494	7H	81.78	3.46	-	-	-	-
12_10053	4H	76.31	6.89	AK370758	-	3	yes
12_10063	4H	44.99	3.28	AK360127	-	non-coding	yes
12_10089	7H	91.12	3.90	-	-	-	-
12_10154	2H	69.05	3.05	AK355324	-	non-coding	yes
12_10159	1H	42.42	2.78	FN555319	pdil4-1	1	no
12_10300	1H	42.42	4.35	EU131177	PR-17c	non-coding	yes
12_10313	0	0	4.12	NM_001061695	Os05g0311000	-	-
12_10392	6H	72.17	3.45	AK361836	-	3	yes
12_10657	7H	61.67	5.82	AK366264	-	3	yes
12_10717	2H	82.98	6.74	-	-	-	-
12_10810	4H	37.88	3.63	AK366265	-	non-coding	yes
12_10824	4H	102.93	2.92	XM_003577507	LOC100843401	non-coding	yes
12_10938	1H	44.59	5.96	AK359548	-	1	no
12_11078	0	0	2.81	AK372406	-	non-coding	yes
12_11151	5H	51.51	5.38	AK354730	-	3	yes
12_11271	1H	136.7	6.00	-	-	-	-
12_11324	2H	72.99	23.02	AK356277	-	non-coding	yes
12_11357	1H	30.15	7.86	AK357047	-	1	no

12_11368	2H	160.46	4.06	AK360506	-	non-coding	yes
12_11468	0	0	3.13	AK359839	-	non-coding	yes
12_20235	2H	61.49	6.27	AK376421	-	non-coding	yes
12_20424	0	0	4.52	AK373140	-	non-coding	yes
12_20649	0	0	3.53	-	-	-	-
12_20760	4H	138.7	5.50	-	-	-	-
12_20793	2H	103.13	3.97	AK371934	-	2	no
12_20981	5H	51.51	4.73	AK370568	-	3	yes
12_21003	0	0	3.04	-	-	-	-
12_21131	1H	59.99	3.29	AK362065	-	3	yes
12_21462	5H	143.29	2.83	AF109194	-	non-coding	yes
12_30005	3H	77.37	2.98	-	-	-	-
12_30060	4H	62.81	10.00	AK365085	-	non-coding	yes
12_30170	3H	92.73	2.95	AK353673	-	3	yes
12_30204	1H	89.77	3.69	AK356336	-	3	yes
12_30226	4H	89.36	8.78	-	-	-	-
12_30242	7H	30.69	3.15	XM_003580160	LOC100839055	1	yes
12_30250	3H	106.67	4.57	AK356601	-	non-coding	yes
12_30275	2H	72.99	3.23	-	-	-	-
12_30342	3H	114.36	3.00	-	-	-	-
12_30389	7H	81.78	3.29	FN179370	AGP-S1a	3	yes
12_30403	1H	128.04	6.73	-	-	-	-
12_30404	1H	42.42	10.32	AK355367	-	3	yes
12_30438	1H	42.42	2.78	AK376647	-	3	yes
12_30475	7H	82.41	23.08	AK372209	-	non-coding	yes
12_30491	2H	49.5	11.08	-	-	-	-
12_30494	5H	171.58	8.49	JX046065	ERS1a	3	yes
12_30504	5H	173.5	4.08	-	-	-	-
12_30528	7H	42.26	9.06	-	-	-	-
12_30574	7H	82.41	4.07	AK370386	-	3	yes
12_30581	7H	79.08	7.89	AK356791	-	3	yes
12_30651	6H	7.87	4.28	AK358128	-	non-coding	yes
12_30674	2H	85.52	2.89	AK369978	-	non-coding	yes
12_30768	5H	42.41	2.81	AK371364	-	non-coding	yes
12_30781	2H	9.12	3.22	AK361785	-	non-coding	yes
12_30976	5H	1.91	3.40	SEG_AY643842S	-	non-coding	yes
12_30988	4H	111.81	3.04	Y14573	Mlo	non-coding	yes
12_30993	4H	49.43	4.61	XM_003577562	LOC100833831	3	yes
12_31032	5H	52.86	8.13	AF326715	adh3	non-coding	yes

12_31035	5H	52.86	9.13	DQ195967	adh3	non-coding	yes
12_31064	5H	51.51	3.07	AK365941	-	3	yes
12_31081	1H	143.2	3.39	AK356376	-	3	yes
12_31183	5H	50.53	3.78	AK362952	-	non-coding	yes
12_31207	0	0	5.27	AK356841	-	3	yes
12_31234	5H	141.88	2.78	AK358248	-	non-coding	yes
12_31270	0	0	3.11	AK361597	-	non-coding	yes
12_31377	1H	128.04	6.56	AK374439	-	3	yes
12_31424	2H	101.72	3.59	AK375713	-	non-coding	yes
12_31486	4H	6.86	9.19	-	-	-	-

Table S8 SNPs with SPA score above 95th percentile genome-wide, including genetic position, GenBank ID, gene short name, in non-coding or coding region (1st, 2nd or 3rd positions), and silent or replacement information

SNP Name	Chr	cM	SPA score	GenBank ID	Gene Short Name	Position	Silent
11_10006	1H	76.92	2.83	AM502852	tub4	3	yes
11_10017	2H	81.31	2.96	AK353943	-	3	yes
11_10056	7H	33.49	3.78	AK364330	-	non-coding	yes
11_10104	5H	141.88	2.86	-	-	-	-
11_10169	7H	106.8	3.34	AK367663	-	non-coding	yes
11_10196	2H	89.68	3.14	AK362580	-	3	yes
11_10253	3H	102.66	2.84	-	-	-	-
11_10477	5H	107.19	3.19	AK354978	-	3	yes
11_10518	5H	88.05	2.93	AK372467	-	1	no
11_10536	5H	155.23	3.25	AK354787	-	3	yes
11_10547	7H	160.97	3.48	AK357875	-	3	yes
11_10557	5H	144.6	2.95	AK371672	-	3	yes
11_10580	5H	29.9	3.02	AK372891	-	non-coding	yes
11_10614	4H	111.81	3.80	AK373775	-	3	yes
11_10619	2H	95.58	3.00	AK373516	-	non-coding	yes
11_10653	3H	69.4	3.67	AK356876	-	2	no
11_10668	4H	50.22	3.04	-	-	-	-
11_10685	2H	72.99	4.37	AK362284	-	3	yes
11_10687	7H	139.9	3.32	AK366838	-	3	yes
11_10939	6H	37.29	3.46	AK371919	-	non-coding	yes
11_11012	7H	149.31	2.93	AK360584	-	1	no
11_11111	6H	139.09	2.80	AK366470	-	non-coding	yes
11_11243	7H	121.36	2.79	-	-	-	-
11_11354	2H	68.07	2.76	AK371682	-	non-coding	yes
11_11432	5H	38.78	3.59	-	-	-	-
11_20018	5H	93.66	3.32	AK376008	-	non-coding	yes
11_20029	6H	133.25	3.16	AK354118	-	1	no
11_20086	2H	110.93	2.96	AK358640	-	non-coding	yes
11_20109	4H	29.34	2.97	AK360657	-	3	yes
11_20133	1H	132.16	2.74	AK361091	-	non-coding	yes
11_20145	4H	1.2	2.76	-	-	-	-
11_20260	1H	42.42	2.73	AK355367	-	3	yes
11_20390	2H	72.99	3.47	FN179383	SBE2a	3	yes
11_20485	7H	91.67	2.97	-	-	-	-
11_20620	6H	78.52	2.78	AK366751	-	non-coding	yes
11_21005	2H	54.92	2.76	-	-	-	-

11_21121	5H	58.65	2.93	AK356836	-	3	yes
11_21192	1H	89.77	4.03	AK357712	-	non-coding	yes
11_21244	5H	51.51	4.22	AK359027	-	3	yes
11_21325	5H	123.12	3.06	AK362100	-	non-coding	yes
11_21340	2H	116.5	2.85	-	-	-	-
11_21399	2H	72.99	4.94	AK357878	-	1	no
11_21406	2H	142.67	3.26	AK370573	-	2	no
11_21447	5H	41.45	3.46	AK364513	-	2	no
11_21452	5H	155.23	2.88	AK372156	-	1	no
11_21502	3H	76.43	3.01	AK356987	-	3	yes
12_10014	3H	173.43	3.22	-	-	-	-
12_10089	7H	91.12	2.76	-	-	-	-
12_10154	2H	69.05	4.02	AK355324	-	non-coding	yes
12_10170	4H	88.7	4.17	AK373474	-	non-coding	yes
12_10199	6H	49.67	3.54	AK376992	-	non-coding	yes
12_10203	5H	59.72	3.93	AK356265	-	3	yes
12_10219	0	0	2.91	AY039003	Xantha-f	3	yes
12_10264	5H	47.04	3.42	WHTE1A	E1	non-coding	yes
12_10284	0	0	3.50	XM_003564030	LOC100843138	3	yes
12_10347	4H	43.72	2.81	AK362515	-	non-coding	yes
12_10392	6H	72.17	3.20	AK361836	-	3	yes
12_10581	7H	82.41	3.14	AK375754	-	3	yes
12_10725	5H	52.86	2.95	AK366248	-	non-coding	yes
12_10810	4H	37.88	3.93	AK366265	-	non-coding	yes
12_10824	4H	102.93	3.58	XM_003577507	LOC100843401	non-coding	yes
12_11151	5H	51.51	4.30	AK354730	-	3	yes
12_11271	1H	136.7	2.77	-	-	-	-
12_11288	2H	67.08	3.93	AK366035	-	3	no
12_11310	3H	13.13	3.29	AK372013	-	3	yes
12_11316	2H	73.89	3.35	AK354712	-	non-coding	yes
12_11324	2H	72.99	5.54	AK356277	-	non-coding	yes
12_11408	0	0	2.73	-	-	-	-
12_11444	1H	54.54	2.76	AJ965495	mcb1	non-coding	yes
12_11498	1H	34.45	3.24	-	-	-	-
12_20278	5H	59.72	3.93	AK356265	-	1	no
12_20413	3H	135.43	2.80	AK356460	-	3	yes
12_20649	0	0	2.80	-	-	-	-
12_20685	7H	94.34	3.24	-	-	-	-
12_20981	5H	51.51	4.22	AK370568	-	3	yes

12_21003	0	0	3.57	-	-	-	-
12_21117	4H	0	4.62	AK359776	-	non-coding	yes
12_21131	1H	59.99	2.90	AK362065	-	3	yes
12_21234	7H	68.89	3.77	AK356095	-	2	no
12_21319	7H	82.41	2.92	XM_003573274	LOC100845308	non-coding	yes
12_30046	4H	105.83	2.94	-	-	-	-
12_30068	2H	67.08	3.93	AK364966	-	3	yes
12_30226	4H	89.36	2.89	-	-	-	-
12_30250	3H	106.67	3.75	AK356601	-	non-coding	yes
12_30344	7H	77.02	2.91	AK359310	-	non-coding	yes
12_30404	1H	42.42	2.79	AK355367	-	3	yes
12_30524	5H	116.66	3.39	AK366243	-	non-coding	yes
12_30577	5H	177.9	2.73	-	-	-	-
12_30581	7H	79.08	2.89	AK356791	-	3	yes
12_30637	6H	72.17	4.10	AK368823	-	non-coding	yes
12_30640	3H	108.7	2.78	AK369817	-	non-coding	yes
12_30644	5H	50.53	2.83	AK374038	-	3	yes
12_30724	2H	72.99	2.83	AK368064	-	3	yes
12_30737	3H	59.83	3.50	AK361759	-	3	yes
12_30745	5H	55.44	3.06	AK366468	-	3	yes
12_30834	5H	88.05	3.20	AK372467	-	non-coding	yes
12_30850	5H	98.2	3.28	DQ480160	CBF4B	non-coding	yes
12_30956	6H	142.2	3.20	-	-	-	-
12_30988	4H	111.81	3.36	Y14573	Mlo	non-coding	yes
12_31017	3H	67.86	3.28	AK356796	-	non-coding	yes
12_31021	2H	82.44	2.85	AF112963	Cht2	non-coding	yes
12_31023	5H	4.15	2.80	AK355143	-	non-coding	yes
12_31032	5H	52.86	3.81	AF326715	adh3	non-coding	yes
12_31035	5H	52.86	3.80	DQ195967	adh3	non-coding	yes
12_31043	6H	112.39	2.77	AY349220	Dhn5	3	yes
12_31062	5H	51.51	3.86	AK365941	-	3	yes
12_31064	5H	51.51	4.23	AK365941	-	3	yes
12_31081	1H	143.2	3.60	AK356376	-	3	yes
12_31100	2H	142.67	3.26	AK370573	-	2	no
12_31179	1H	61.39	2.79	AY738115	cbp1	3	yes
12_31189	2H	67.08	2.76	AK364573	-	3	yes
12_31202	0	0	2.73	AK358464	-	non-coding	yes
12_31246	4H	92.41	3.24	AK362249	-	non-coding	yes
12_31411	0	0	2.81	AK372597	-	2	no

12_31486	4H	6.86	3.75	-	-	-	-
12_31511	0	0	3.01	AK360621	-	non-coding	yes
12_31525	3H	134.05	3.32	AK364139	-	2	no
