# Supplementary material for: Two Genomic Regions Contribute Disproportionately to Geographic Differentiation in Wild Barley
Source: G3 (Bethesda). 2014 Apr 22;4(7):1193–203. doi: 10.1534/g3.114.010561 (PMC4455769; doi:10.1534/g3.114.010561)
Supplement: Supporting Information [file supp_g3.114.010561_FigureS1.pdf]

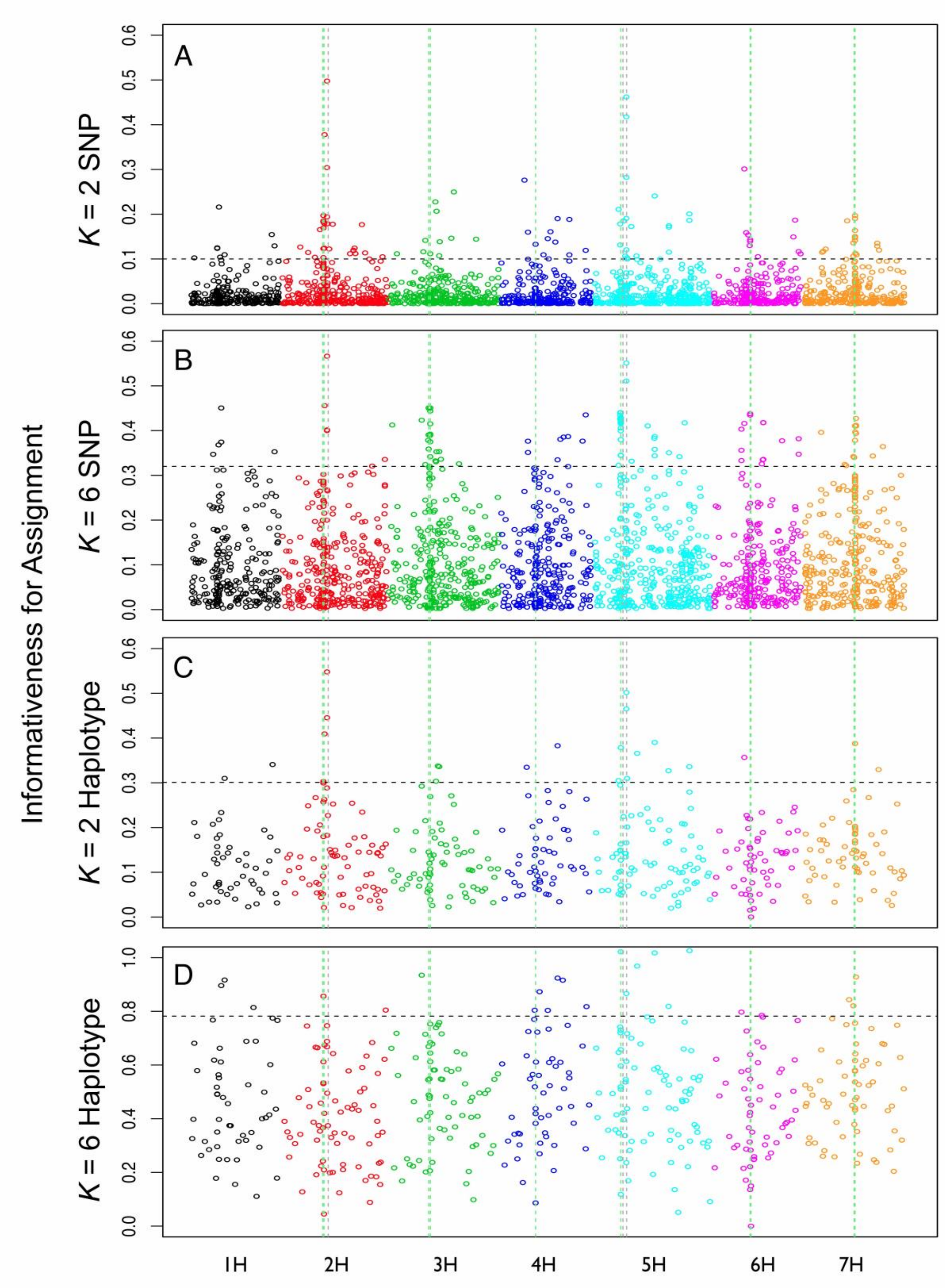

Figure S1 The informativeness for assignment for all SNPs (A, B) and five-SNP haplotypes (C, D) genome-wide based on (A, C) *K* = 2 and (B, D) *K* = 6. The horizontal dashed line is the 95th percentile. The grey vertical dashed lines delineate the two high F_{ST} regions based on comparison between the Eastern and Western populations. The green vertical dashed lines indicate the centromeric regions. Note the scale of y-axis in panel D.
