# Supplementary material for: Two Genomic Regions Contribute Disproportionately to Geographic Differentiation in Wild Barley
Source: G3 (Bethesda). 2014 Apr 22;4(7):1193–203. doi: 10.1534/g3.114.010561 (PMC4455769; doi:10.1534/g3.114.010561)
Supplement: Supporting Information [file supp_g3.114.010561_FigureS2.pdf]

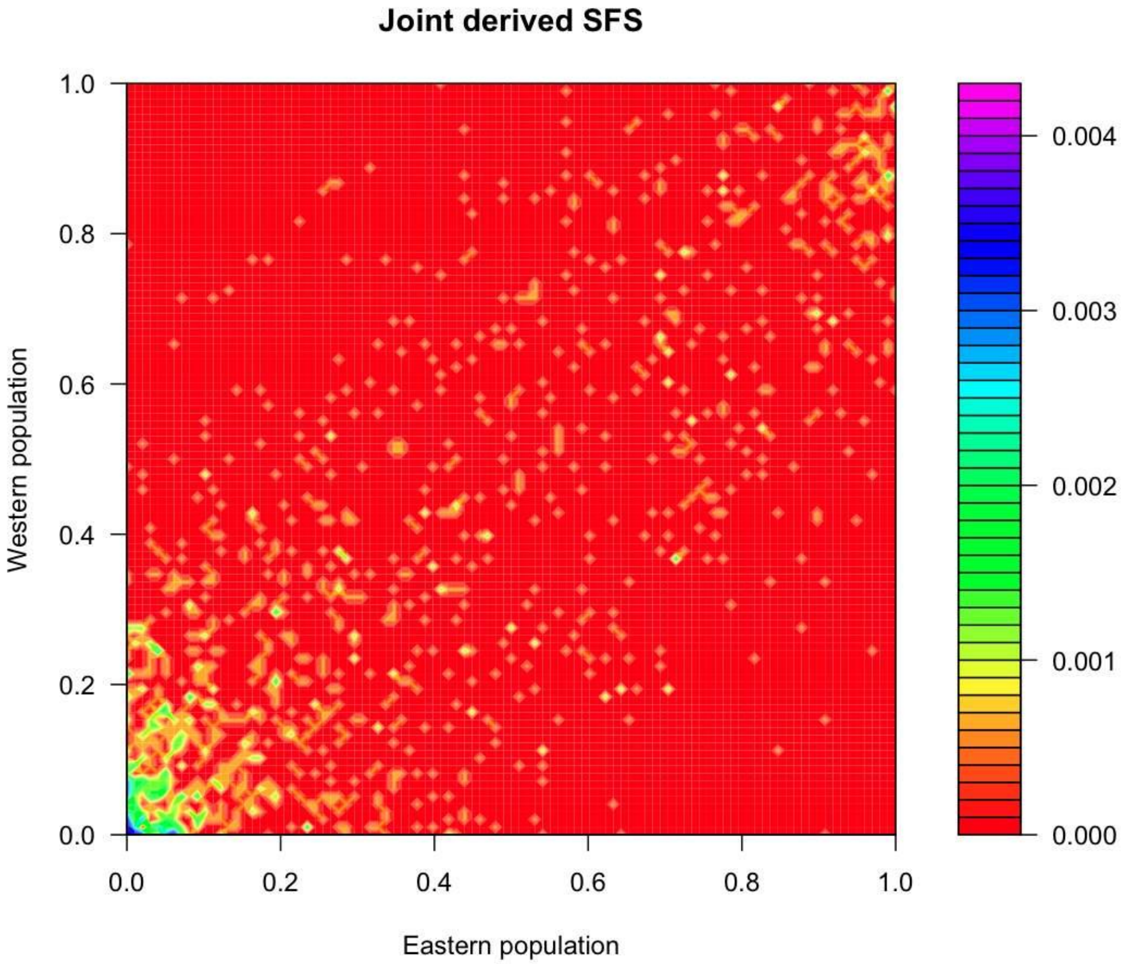

Figure S2 The joint unfolded site frequency spectrum based on all accessions from the Eastern population (upper triangle) and Western population (lower triangle). The comparison includes 1633 SNPs for which the ancestral state could be inferred by comparison to *Hordeum bulbosum* Illumina RNA-Seq data.
