# Supplementary material for: Two Genomic Regions Contribute Disproportionately to Geographic Differentiation in Wild Barley
Source: G3 (Bethesda). 2014 Apr 22;4(7):1193–203. doi: 10.1534/g3.114.010561 (PMC4455769; doi:10.1534/g3.114.010561)
Supplement: Supporting Information [file supp_g3.114.010561_FigureS3.pdf]

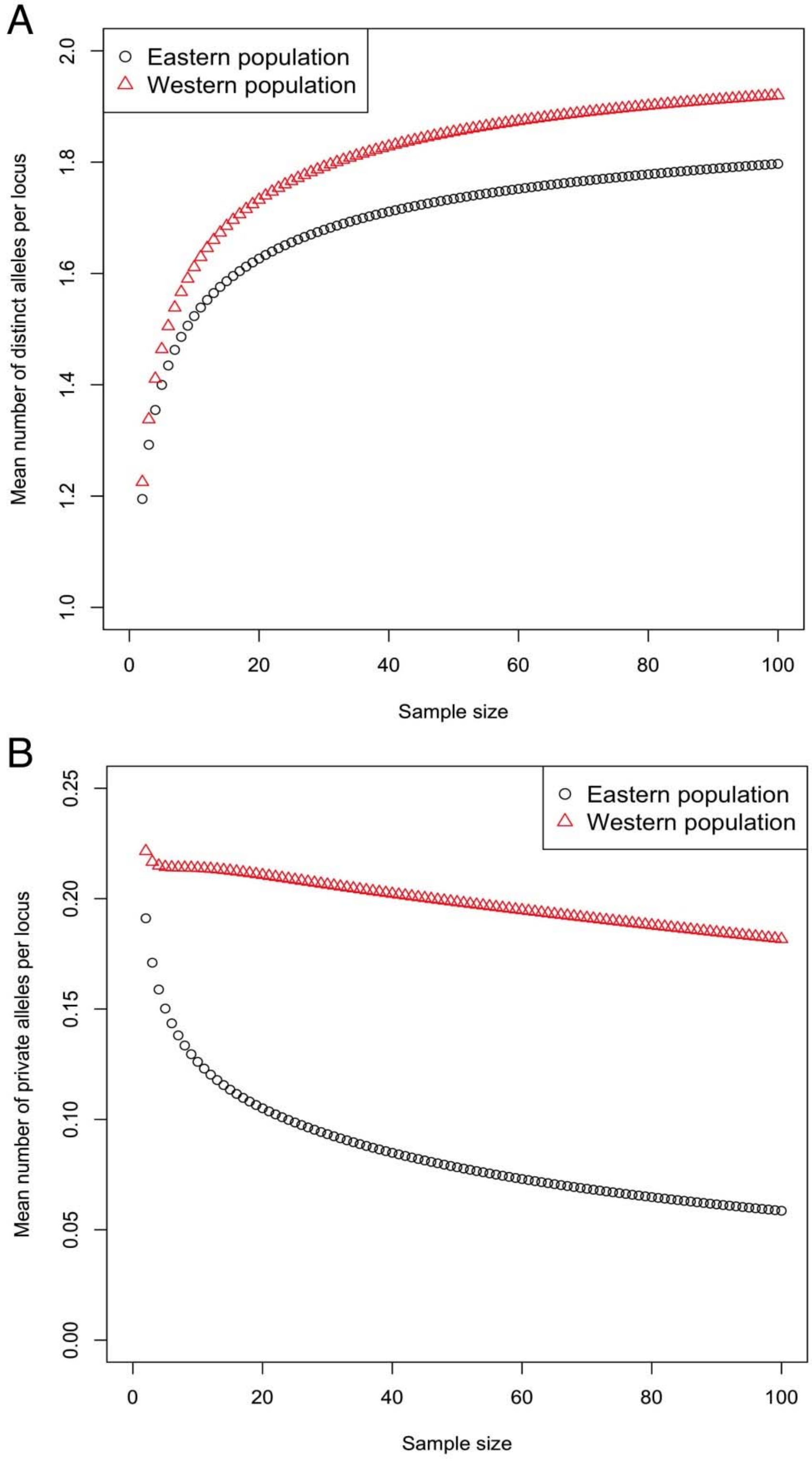

Figure S3 Rarefaction analysis comparing nucleotide diversity between the Eastern and Western populations, (A) mean number of distinct alleles per locus and (B) mean number of private alleles per locus.
