# Supplementary material for: Two Genomic Regions Contribute Disproportionately to Geographic Differentiation in Wild Barley
Source: G3 (Bethesda). 2014 Apr 22;4(7):1193–203. doi: 10.1534/g3.114.010561 (PMC4455769; doi:10.1534/g3.114.010561)
Supplement: Supporting Information [file supp_g3.114.010561_FigureS4.pdf]

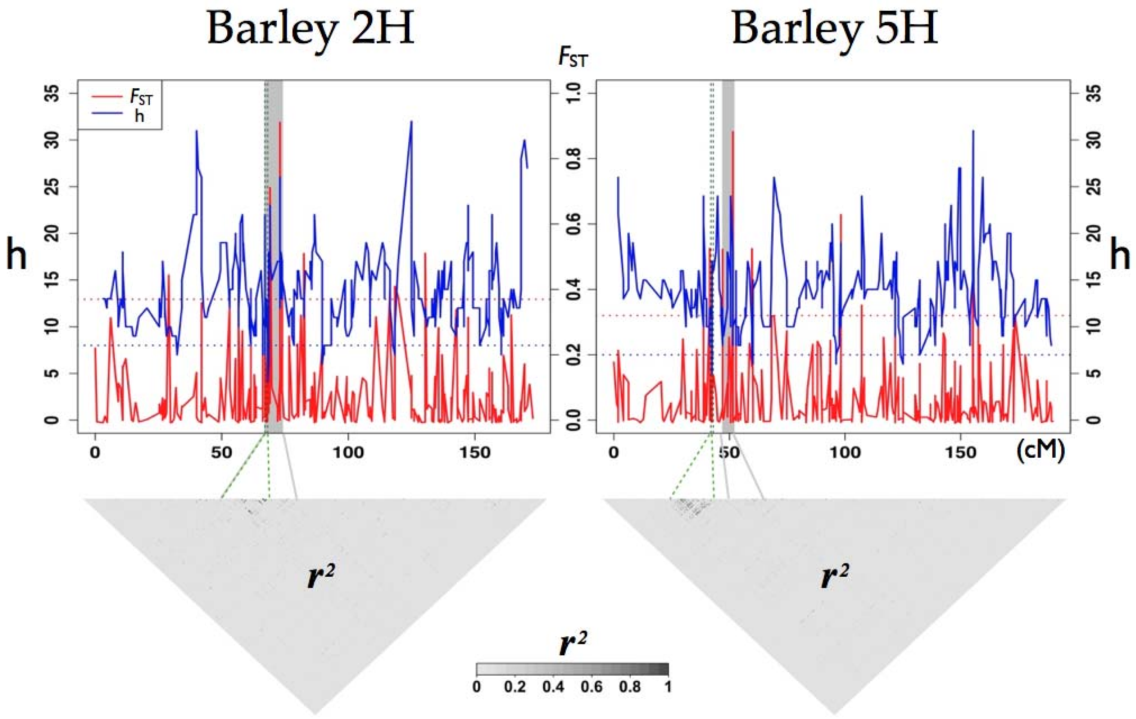

Figure S4 Population genetic analysis of the two high F_{ST} regions. Top panel, haplotype number (blue curve) and F_{ST} between the Eastern and Western populations (red curve). The number of haplotypes present across linkage group 2H and 5H was calculated in overlapping 5-SNP windows with 1-SNP increments. The high F_{ST} regions are marked in grey and the centromeres by green dashed lines. Below, LD (r^2) is plotted across 2H and 5H.
