# Supplementary figures and images for: Two Genomic Regions Contribute Disproportionately to Geographic Differentiation in Wild Barley
Source: G3 (Bethesda). 2014 Apr 22;4(7):1193–203. doi: 10.1534/g3.114.010561 (PMC4455769; doi:10.1534/g3.114.010561)

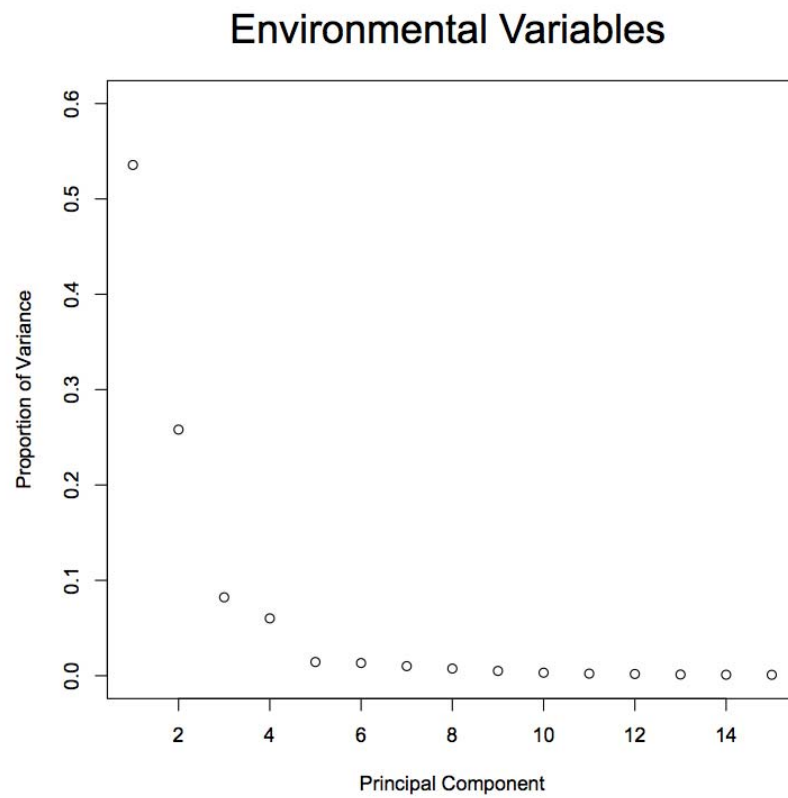

**Figure S5** The proportion of variance explained by each PC of environmental variables.

Supplement: Supporting Information [file supp_g3.114.010561_FigureS5.pdf]
