# Supplementary material for: Two Genomic Regions Contribute Disproportionately to Geographic Differentiation in Wild Barley
Source: G3 (Bethesda). 2014 Apr 22;4(7):1193–203. doi: 10.1534/g3.114.010561 (PMC4455769; doi:10.1534/g3.114.010561)
Supplement: Supporting Information [file supp_g3.114.010561_FigureS6.pdf]

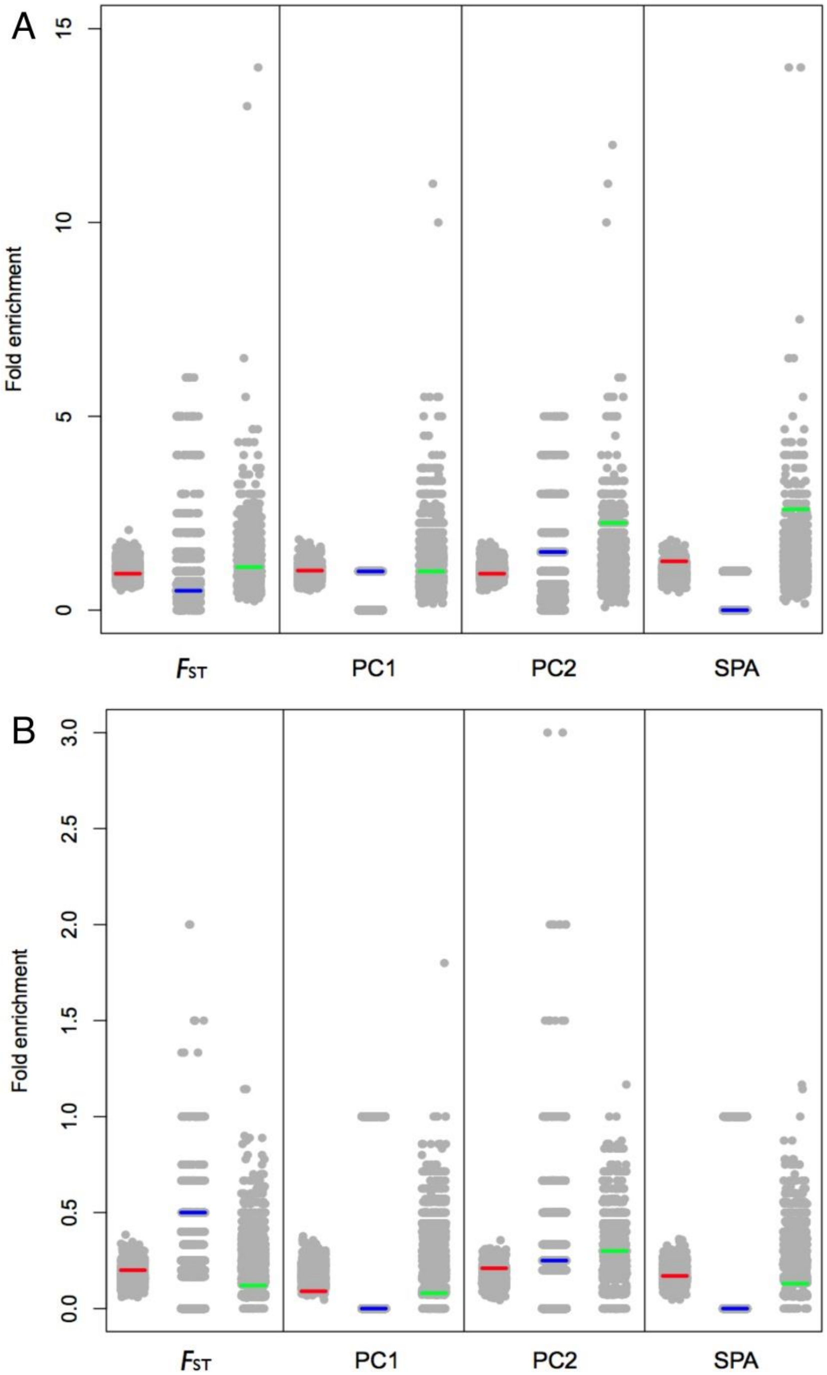

Figure S6 Enrichment analysis for (A) genic versus non-genic and (B) nonsynonymous versus synonymous SNPs. Enrichment analysis for candidates involved resampling the number of SNPs in each candidate list (outliers in F_{ST}, environmental association or SPA analyses) randomly from the genome 1000 times. Enrichment analysis was performed for all SNPs genome-wide, centromeric regions, and the two putative translocations or inversions. The observed values from the data is indicated by the colored horizontal line, red: genome-wide; blue: centromeric regions; green: the two putative structural rearrangements.
