# Supplementary material for: Two Genomic Regions Contribute Disproportionately to Geographic Differentiation in Wild Barley
Source: G3 (Bethesda). 2014 Apr 22;4(7):1193–203. doi: 10.1534/g3.114.010561 (PMC4455769; doi:10.1534/g3.114.010561)
Supplement: Supporting Information [file supp_g3.114.010561_TableS1.pdf]

Table S1 284 wild barley accessions used in this study, including their latitude and longitude information

Accession	Latitude	Longitude
WBDC001	36.22	36.7
WBDC002	35.71	37.44
WBDC004	35.97	36.54
WBDC005	32.45	35.92
WBDC006	32.3	36.17
WBDC007	32.33	35.92
WBDC008	32.67	35.62
WBDC009	31.53	35.75
WBDC010	36.67	65.73
WBDC011	36	43.52
WBDC012	36.28	64.82
WBDC013	35.53	44.83
WBDC014	35.75	69
WBDC015	34.35	62.18
WBDC017	32.74	35.82
WBDC018	34.98	63.12
WBDC019	36.75	45.72
WBDC021	34.8	45.6
WBDC022	39.78	30.52
WBDC023	33.5	48.45
WBDC024	31.28	48.72
WBDC025	30.3	66.9
WBDC026	37.65	69.1
WBDC028	31.95	34.93
WBDC029	31.58	34.62
WBDC030	33.08	35.18
WBDC031	32.75	35.08
WBDC032	33	35.13
WBDC033	31.27	35.22
WBDC034	31.85	34.92
WBDC035	31.43	34.59
WBDC036	34.57	63
WBDC037	32.98	35.5
WBDC038	46.77	35.21
WBDC039	32.03	35.83
WBDC040	32.7	34.95
WBDC041	31.67	34.57
WBDC042	32.97	35.53
WBDC043	32.98	35.58
WBDC044	32.17	34.83
WBDC046	32.48	35.9
WBDC047	32.53	36.07
WBDC048	37.25	44.48
WBDC049	37.25	44.48
WBDC051	34.76	39.03
WBDC052	32.32	36.02

WBDC054	34.57	38.36
WBDC055	35.61	35.82
WBDC056	36.72	37.12
WBDC058	34.99	34.02
WBDC059	34.98	34.05
WBDC060	31.35	27.17
WBDC061	36.16	36.58
WBDC062	36.38	36.84
WBDC063	36.72	36.64
WBDC064	35.8	36.24
WBDC066	33.78	36.4
WBDC067	33.7	36.38
WBDC068	32.82	36.72
WBDC070	35.61	35.82
WBDC072	32.78	21.63
WBDC073	32.08	21.87
WBDC074	32.8	22.05
WBDC075	32.7	21.92
WBDC078	36.77	40.86
WBDC079	32.25	35.88
WBDC080	32.53	35.78
WBDC081	32.48	35.65
WBDC083	32.17	35.92
WBDC085	31.98	35.65
WBDC089	31.83	35.8
WBDC095	31.18	35.62
WBDC097	31.28	35.83
WBDC100	30.77	35.62
WBDC101	30.7	35.57
WBDC102	30.58	35.57
WBDC103	30.2	35.47
WBDC104	31.18	35.62
WBDC105	32.65	35.83
WBDC106	34.94	36.74
WBDC107	34.75	36.73
WBDC108	33.94	36.71
WBDC109	32.77	36.72
WBDC110	32.78	36.79
WBDC111	33.84	36.54
WBDC112	33.98	36.59
WBDC113	37.92	58.17
WBDC115	38.33	55.87
WBDC116	38.32	56.38
WBDC117	38.17	56.05
WBDC119	40.08	67.58
WBDC120	39.47	67.5
WBDC121	29.72	51.78
WBDC122	32.87	48.17
WBDC123	36.42	58.47

WBDC124	35.02	46.45
WBDC125	38.8	66.47
WBDC126	33.45	35.32
WBDC127	32.94	36.73
WBDC128	33.84	36.17
WBDC129	32.81	36.42
WBDC130	32.83	36.18
WBDC131	33.01	36.04
WBDC132	33.47	35.87
WBDC133	33.62	35.82
WBDC134	33.62	35.77
WBDC135	33.52	35.72
WBDC136	33.52	35.77
WBDC137	33.45	35.82
WBDC138	33.42	35.76
WBDC139	33.93	36.1
WBDC140	34.2	36.08
WBDC141	34.13	36.03
WBDC142	34.02	36.08
WBDC143	33.57	35.9
WBDC145	33.8	36.02
WBDC146	36.75	45.7
WBDC147	37.5	45.17
WBDC148	37.07	45.47
WBDC149	38.08	45
WBDC150	38.5	47.2
WBDC151	36.52	36.95
WBDC152	35.5	51.25
WBDC155	36.33	42.17
WBDC156	36.42	41.65
WBDC157	36.38	43.42
WBDC158	35.37	43.2
WBDC159	32.68	36.79
WBDC160	32.96	36.6
WBDC161	36.65	37.58
WBDC164	37.01	41.64
WBDC165	37.29	42.21
WBDC167	36.4	40.36
WBDC168	33.57	35.72
WBDC169	33.5	35.78
WBDC170	33.63	35.95
WBDC171	33.58	35.85
WBDC174	36.27	47.77
WBDC175	36.27	47.77
WBDC177	36.35	43.13
WBDC178	36.08	43.28
WBDC179	32.5	20.9
WBDC180	31.83	24.23
WBDC181	32.02	36.02

WBDC183	32.63	35.72
WBDC184	32.37	21.17
WBDC185	32.77	21.72
WBDC186	36.88	37.35
WBDC187	36.69	37.46
WBDC188	36.88	37.52
WBDC189	36.76	37.63
WBDC191	36.84	37.72
WBDC192	37.04	37.61
WBDC193	37.32	37.47
WBDC194	36.97	37.19
WBDC195	36.81	37.22
WBDC196	36.99	36.93
WBDC197	36.48	37.77
WBDC198	34.82	36.47
WBDC199	34.91	36.64
WBDC200	34.94	36.66
WBDC201	35.56	36.89
WBDC202	35.6	36.55
WBDC203	35.63	36.45
WBDC204	38.58	57.12
WBDC205	42.05	48.27
WBDC206	32.55	36.6
WBDC207	40.34	71.18
WBDC208	41.61	69.91
WBDC209	40.13	68.4
WBDC210	39.93	67.68
WBDC212	40.01	67.09
WBDC213	39.55	66.56
WBDC214	39.4	67.02
WBDC215	38.28	56.29
WBDC216	38.73	56.85
WBDC217	40.2	44.53
WBDC218	43.06	73.66
WBDC220	42.42	69.7
WBDC221	40.13	69.33
WBDC222	40.13	69.33
WBDC223	39.97	69
WBDC225	40.12	69.2
WBDC228	40.49	49.19
WBDC229	40.53	48.89
WBDC230	40.61	48.88
WBDC231	40.71	48.8
WBDC232	40.7	48.63
WBDC233	35.93	68.7
WBDC234	35.15	33.88
WBDC236	31.6	35.62
WBDC237	31.53	35.62
WBDC238	31.55	35.85

WBDC240	31.88	35.94
WBDC241	31.78	36.22
WBDC242	32.12	35.87
WBDC243	32.03	35.67
WBDC244	32.08	35.71
WBDC245	32.17	35.87
WBDC246	32.68	35.75
WBDC247	32.37	35.7
WBDC248	32.53	35.85
WBDC250	32.58	35.93
WBDC252	32.28	35.68
WBDC253	32.35	35.82
WBDC254	32.62	35.7
WBDC255	32.35	35.67
WBDC256	32.33	35.63
WBDC257	31.18	35.7
WBDC258	31.28	35.75
WBDC260	30.5	35.5
WBDC261	30.52	35.53
WBDC262	30.3	35.48
WBDC263	30.38	35.47
WBDC265	30.93	35.67
WBDC266	30.88	35.67
WBDC268	31.75	36.76
WBDC269	33.42	35.87
WBDC270	31.68	34.87
WBDC271	31.43	34.48
WBDC274	30.87	34.77
WBDC275	31.58	34.55
WBDC277	32.15	34.93
WBDC278	31.83	34.77
WBDC279	31.82	34.78
WBDC280	32.02	34.83
WBDC281	33.03	35.58
WBDC282	32.68	35.22
WBDC284	32.67	35.6
WBDC285	32.98	35.45
WBDC286	32.97	35.47
WBDC287	32.5	35.5
WBDC288	32.98	35.42
WBDC289	32.52	35.48
WBDC290	31.67	34.93
WBDC291	31.72	34.92
WBDC292	31.8	35.02
WBDC293	31.8	35.17
WBDC294	31.75	34.98
WBDC295	37.08	42.07
WBDC296	37.06	41.56
WBDC297	37.06	41.75

WBDC298	37.08	41.09
WBDC299	36.66	36.71
WBDC300	36.37	36.86
WBDC302	33.37	35.88
WBDC303	33.74	36.11
WBDC304	33.64	35.96
WBDC305	33.66	36
WBDC306	33.73	36.13
WBDC307	33.2	36.07
WBDC308	32.6	36.38
WBDC309	35.03	37.01
WBDC310	34.54	36.92
WBDC312	34.47	36.76
WBDC315	34.94	36.85
WBDC316	36.23	36.55
WBDC317	35.52	38.76
WBDC318	32.6	36.74
WBDC319	32.49	36.68
WBDC320	32.42	36.62
WBDC323	37.9	58.55
WBDC324	35.83	61.46
WBDC326	37.72	58.6
WBDC329	38.18	55.6
WBDC330	38.77	56.29
WBDC331	39.24	56.11
WBDC332	38.43	56.42
WBDC333	38.41	56.49
WBDC334	38.27	56.31
WBDC335	39.25	55.62
WBDC336	38.42	56.68
WBDC337	37.23	37.52
WBDC338	37.27	37.54
WBDC340	37.25	37.33
WBDC341	36.95	36.93
WBDC342	36.97	36.9
WBDC343	36.87	36.95
WBDC345	38.95	66.83
WBDC346	39.92	66.37
WBDC347	37.8	67
WBDC348	32.44	35
WBDC349	32.14	35.14
WBDC355	40.31	48.81
