# Supplementary material for: Two Genomic Regions Contribute Disproportionately to Geographic Differentiation in Wild Barley
Source: G3 (Bethesda). 2014 Apr 22;4(7):1193–203. doi: 10.1534/g3.114.010561 (PMC4455769; doi:10.1534/g3.114.010561)
Supplement: Supporting Information [file supp_g3.114.010561_TableS2.pdf]

Table S2 The name, repeat length, repeat unit length, total size and observed heterozygosity of the 29 microsatellites used in this study

Microsatellite	# Repeat	Repeat unit length	Total size (bp)	Observed Heterozygosity
Bmag905	14	2	178-228	0.01
Bmag006	17	2	105-223	0.10
Bmac129	28	2	132-204	0.00
Bmac67	18	2	130-262	0.04
Bmag749	11	2	93-199	0.01
Bmac134	28	2	117-189	0.01
Bmac156	(AC)22(AT)5	2	103-199	0.01
Bmac213	23	2	131-213	0.09
Bmac316	19	2	138-234	0.01
Bmag369	16	2	196-216	0.00
Bmag718	(GA)18(AG)6	2	165-217	0.02
Bmag877	15	2	153-289	0.04
EBmac603	10	2	155-257	0.09
HVM06	9	2	118-202	0.23
HVMLOH1A	6	2	147-203	0.00
GMS1	(CT)7TTT(CT)2	2	123-161	0.18
Bmag0496	20	2	139-287	0.03
HVHVA1	5	3	134-140	0.01
Bmac18	11	2	131-145	0.00
Bmag382	(AG)7AA(AG)7	2	103-109	0.00
Scssr02748	12	2	144-158	0.01
Scssr10148	10	2	178-230	0.04
Scssr08447	6	3	172-182	0.00
Scssr05939	5	2	150-160	0.03
Bmag211	16	2	150-198	0.02
Hvltppb	10	2	205-229	0.75
Scssr02306	13	2	150-164	0.00
Scssr15864	4	3	146-184	0.08
Scssr25691	17	2	208-250	0.07
