# Supplementary material for: Two Genomic Regions Contribute Disproportionately to Geographic Differentiation in Wild Barley
Source: G3 (Bethesda). 2014 Apr 22;4(7):1193–203. doi: 10.1534/g3.114.010561 (PMC4455769; doi:10.1534/g3.114.010561)
Supplement: Supporting Information [file supp_g3.114.010561_TableS4.pdf]

Table S4 (A) Environmental variables and abbreviations used in this study; (B) Environmental variable and the corresponding loadings for the first two principal components

(A)

Environmental variable	Abbreviation
Annual mean temperature	bio1
Mean diurnal range	bio2
Isothermality (bio2/bio7)*100 [a]	bio3
Temperature seasonality (standard deviation*100)	bio4
Min temperature of the coldest month	bio6
Mean temperature of the wettest quarter	bio8
Mean temperature of the driest quarter	bio9
Mean temperature of the coldest quarter	bio11
Annual precipitation	bio12
Precipitation of the wettest month	bio13
Precipitation of the driest month	bio14
Precipitation seasonality (coefficient of variation)	bio15
Precipitation of the wettest quarter	bio16
Precipitation of the driest quarter	bio17
Precipitation of the coldest quarter	bio19
Monthly minimum and maximum temperature	tmin#, tmax#
Monthly total precipitation	prec#

[a] bio7: temperature annual range

(B)

PC1		PC2	
Variable	Loadings	Variable	Loadings
bio11	-0.223	bio12	-0.316
tmin2	-0.222	bio16	-0.314
tmax1	-0.222	bio13	-0.310
tmax2	-0.221	prec2	-0.306
tmax12	-0.221	bio19	-0.303
tmax11	-0.220	prec1	-0.297
bio6	-0.220	prec12	-0.295
tmin1	-0.220	prec11	-0.292
tmin3	-0.220	prec3	-0.286
tmin12	-0.218	prec10	-0.180
tmin11	-0.215	prec4	-0.128
tmin10	-0.215	alt	-0.089
tmax3	-0.211	bio15	-0.058

bio1	-0.209	tmin11	-0.039
tmax10	-0.201	tmin12	-0.038
tmin4	-0.196	tmin1	-0.031
tmax4	-0.179	bio6	-0.030
bio3	-0.178	tmin10	-0.019
bio15	-0.176	tmin2	-0.013
bio9	-0.116	bio2	0.199
bio8	-0.111	tmax4	0.147
prec12	-0.080	bio8	0.091
prec1	-0.079	bio4	0.088
bio19	-0.068	tmax3	0.086
prec11	-0.058	bio9	0.086
bio13	-0.052	tmin4	0.071
bio16	-0.048	tmax10	0.068
prec2	-0.047	bio1	0.065
bio12	0.003	tmax2	0.047
prec3	0.020	bio3	0.041
bio2	0.026	tmax11	0.027
prec10	0.091	tmax1	0.023
bio14	0.112	tmax12	0.022
bio17	0.120	tmin3	0.018
prec4	0.141	bio14	0.012
alt	0.154	bio17	0.004
bio4	0.162	bio11	0.003
