# Supplementary material for: Two Genomic Regions Contribute Disproportionately to Geographic Differentiation in Wild Barley
Source: G3 (Bethesda). 2014 Apr 22;4(7):1193–203. doi: 10.1534/g3.114.010561 (PMC4455769; doi:10.1534/g3.114.010561)
Supplement: Supporting Information [file supp_g3.114.010561_Table_S5.pdf]

**Table S5 BOPA SNPs used in this study, including their genetic positions**

| SNP | Chr | Position |
|---:|---:|---:|
| 11_20001 | 4H | 31.69 |
| 11_20002 | 3H | 65.25 |
| 11_20003 | 5H | 121.67 |
| 11_20005 | 6H | 132.6 |
| 11_10002 | 1H | 64.44 |
| 11_10003 | 6H | 56.06 |
| 11_20008 | 5H | 141.48 |
| 11_20009 | 3H | 118.22 |
| 11_10005 | 3H | 71.26 |
| 11_10006 | 1H | 76.92 |
| 11_20010 | 5H | 12.8 |
| 11_10008 | 3H | 67.86 |
| 11_20012 | 4H | 43.72 |
| 11_20013 | 4H | 146.48 |
| 11_20015 | 6H | 74.65 |
| 11_20014 | 7H | 11.87 |
| 11_20017 | 3H | 82.62 |
| 11_20018 | 5H | 93.66 |
| 11_10010 | 4H | 77.66 |
| 11_20020 | 4H | 63.79 |
| 11_10011 | 3H | 67.86 |
| 11_20021 | 1H | 111.81 |
| 11_20023 | 3H | 122.7 |
| 11_20022 | 5H | 177.9 |
| 11_10012 | 2H | 67.08 |
| 11_10015 | 6H | 111.08 |
| 11_10017 | 2H | 81.31 |
| 11_20029 | 6H | 133.25 |
| 11_20032 | 2H | 68.07 |
| 11_10023 | 6H | 24.91 |
| 11_10024 | 5H | 96.12 |
| 11_20036 | 6H | 118.15 |
| 11_20039 | 2H | 67.63 |
| 11_10025 | 7H | 14.39 |
| 11_10026 | 3H | 34.15 |
| 11_20042 | 7H | 88.06 |
| 11_10028 | 4H | 56.22 |
| 11_20044 | 4H | 115.37 |
| 11_10030 | 1H | 17.4 |
| 11_20052 | 6H | 44.96 |
| 11_10031 | 4H | 30.37 |
| 11_20053 | 6H | 81.79 |
| 11_20058 | 6H | 65.83 |
| 11_20060 | 7H | 65.98 |
| 11_20064 | 2H | 124.98 |
| 11_20063 | 3H | 97.14 |
| 11_20072 | 4H | 78.08 |

| | | |
|---|---|---|
| 11_10041 | 1H | 138.86 |
| 11_20078 | 5H | 155.66 |
| 11_20080 | 2H | 107.04 |
| 11_10043 | 1H | 63.74 |
| 11_10044 | 3H | 134.71 |
| 11_20085 | 3H | 146.25 |
| 11_20086 | 2H | 110.93 |
| 11_10046 | 4H | 62.81 |
| 11_20089 | 4H | 145.7 |
| 11_10047 | 3H | 90.1 |
| 11_20092 | 7H | 111.15 |
| 11_10048 | 4H | 46.97 |
| 11_20093 | 3H | 93.81 |
| 11_20095 | 1H | 58.59 |
| 11_10050 | 7H | 57.57 |
| 11_20096 | 5H | 70.9 |
| 11_10052 | 4H | 76.31 |
| 11_20097 | 5H | 88.05 |
| 11_20102 | 3H | 65.25 |
| 11_20103 | 7H | 104.32 |
| 11_20104 | 5H | 149.94 |
| 11_20105 | 5H | 49.83 |
| 11_20107 | 2H | 14.42 |
| 11_10055 | 7H | 81.78 |
| 11_20109 | 4H | 29.34 |
| 11_10056 | 7H | 33.49 |
| 11_10057 | 2H | 67.08 |
| 11_10058 | 5H | 42.41 |
| 11_10061 | 6H | 44.96 |
| 11_20113 | 7H | 49.61 |
| 11_20114 | 4H | 44.99 |
| 11_20115 | 3H | 92.73 |
| 11_20117 | 7H | 156.13 |
| 11_20118 | 6H | 106.15 |
| 11_20119 | 4H | 109.65 |
| 11_20121 | 1H | 80.47 |
| 11_20126 | 7H | 31.35 |
| 11_10069 | 7H | 83.42 |
| 11_10070 | 2H | 66.2 |
| 11_20127 | 5H | 116.66 |
| 11_10072 | 2H | 165.28 |
| 11_20129 | 5H | 42.41 |
| 11_20130 | 3H | 108.7 |
| 11_20131 | 2H | 54.27 |
| 11_10075 | 1H | 45.2 |
| 11_20133 | 1H | 132.16 |
| 11_20134 | 5H | 95.11 |
| 11_20135 | 4H | 63.79 |
| 11_10080 | 5H | 143.29 |

| | | |
|---|---|---|
| 11_20136 | 3H | 100.29 |
| 11_20138 | 1H | 140.69 |
| 11_20139 | 7H | 141.15 |
| 11_10081 | 3H | 39.92 |
| 11_20145 | 4H | 1.2 |
| 11_20149 | 1H | 98.68 |
| 11_20153 | 1H | 105.77 |
| 11_10090 | 4H | 86.01 |
| 11_20155 | 3H | 162.83 |
| 11_10092 | 2H | 137.03 |
| 11_10093 | 4H | 56.22 |
| 11_10094 | 5H | 114.14 |
| 11_20159 | 3H | 6.31 |
| 11_20160 | 2H | 68.07 |
| 11_10095 | 5H | 132.32 |
| 11_20162 | 7H | 26.14 |
| 11_20168 | 3H | 124.04 |
| 11_20170 | 7H | 162.45 |
| 11_20172 | 3H | 18.21 |
| 11_10101 | 2H | 170.73 |
| 11_10104 | 5H | 141.88 |
| 11_20178 | 4H | 95.22 |
| 11_20179 | 5H | 42.41 |
| 11_20180 | 4H | 44.99 |
| 11_20182 | 2H | 127.74 |
| 11_20184 | 6H | 61.19 |
| 11_10112 | 3H | 14 |
| 11_20189 | 5H | 172.83 |
| 11_20192 | 7H | 29.7 |
| 11_10113 | 4H | 19.45 |
| 11_20193 | 3H | 42.31 |
| 11_20195 | 7H | 67.02 |
| 11_10116 | 5H | 41.45 |
| 11_20197 | 4H | 90.77 |
| 11_20200 | 7H | 81.78 |
| 11_10120 | 6H | 6.54 |
| 11_10121 | 7H | 2.13 |
| 11_10124 | 6H | 74.65 |
| 11_20205 | 7H | 83.42 |
| 11_10128 | 2H | 124.98 |
| 11_10129 | 6H | 44.96 |
| 11_10130 | 7H | 149.31 |
| 11_20210 | 4H | 26.71 |
| 11_10132 | 4H | 26.2 |
| 11_20211 | 6H | 133.9 |
| 11_20212 | 6H | 1.03 |
| 11_20215 | 2H | 147.37 |
| 11_20219 | 0 | 0 |
| 11_20220 | 1H | 111.15 |

| | | |
|---|---|---|
| 11_10136 | 6H | 27.33 |
| 11_10137 | 3H | 67.86 |
| 11_10138 | 2H | 108.58 |
| 11_20222 | 3H | 91.79 |
| 11_20226 | 5H | 1.91 |
| 11_20230 | 7H | 88.06 |
| 11_10143 | 7H | 91.12 |
| 11_20232 | 6H | 0 |
| 11_10147 | 2H | 60.89 |
| 11_20236 | 5H | 68.21 |
| 11_10153 | 7H | 79.08 |
| 11_10157 | 5H | 42.41 |
| 11_10158 | 3H | 73.3 |
| 11_10161 | 5H | 155.45 |
| 11_10165 | 6H | 23.62 |
| 11_20245 | 7H | 7.87 |
| 11_10167 | 5H | 71.93 |
| 11_20247 | 7H | 116.28 |
| 11_20249 | 7H | 44.12 |
| 11_10169 | 7H | 106.8 |
| 11_20251 | 2H | 68.56 |
| 11_10172 | 3H | 82.62 |
| 11_10174 | 7H | 162.03 |
| 11_10175 | 6H | 130.38 |
| 11_10177 | 5H | 42.41 |
| 11_10178 | 2H | 40.03 |
| 11_20259 | 5H | 125.09 |
| 11_10180 | 2H | 20.45 |
| 11_20260 | 1H | 42.42 |
| 11_20262 | 6H | 10.72 |
| 11_10182 | 7H | 129.32 |
| 11_10184 | 3H | 110.75 |
| 11_10185 | 6H | 93.44 |
| 11_10186 | 1H | 22.04 |
| 11_20265 | 5H | 53.84 |
| 11_20266 | 6H | 65.29 |
| 11_10189 | 6H | 65.83 |
| 11_10191 | 2H | 72.99 |
| 11_20267 | 1H | 105.77 |
| 11_10194 | 2H | 67.08 |
| 11_20269 | 4H | 54.73 |
| 11_10196 | 2H | 89.68 |
| 11_20273 | 3H | 83.58 |
| 11_10202 | 6H | 102.62 |
| 11_20276 | 3H | 71.26 |
| 11_20283 | 5H | 51.51 |
| 11_10208 | 4H | 2.6 |
| 11_10213 | 2H | 99.39 |
| 11_10214 | 2H | 105.62 |

| | | |
|---|---|---|
| 11_20287 | 6H | 73.84 |
| 11_20288 | 3H | 65.25 |
| 11_20289 | 4H | 58.2 |
| 11_10216 | 2H | 26.63 |
| 11_10217 | 5H | 149.94 |
| 11_20291 | 6H | 52.85 |
| 11_20293 | 2H | 161.3 |
| 11_20294 | 6H | 6.54 |
| 11_10223 | 4H | 19.45 |
| 11_10224 | 3H | 67.27 |
| 11_10225 | 3H | 69.4 |
| 11_20298 | 5H | 125.09 |
| 11_20300 | 5H | 122.33 |
| 11_10232 | 7H | 29.05 |
| 11_10234 | 2H | 54.27 |
| 11_20303 | 7H | 0 |
| 11_10236 | 5H | 172.83 |
| 11_20306 | 5H | 50.53 |
| 11_20307 | 7H | 6.79 |
| 11_20309 | 0 | 0 |
| 11_10240 | 5H | 42.41 |
| 11_10243 | 2H | 67.08 |
| 11_20311 | 0 | 0 |
| 11_10244 | 6H | 48.42 |
| 11_20315 | 6H | 27.33 |
| 11_10247 | 4H | 87.01 |
| 11_10251 | 0 | 0 |
| 11_10252 | 5H | 42.41 |
| 11_10253 | 3H | 102.66 |
| 11_10254 | 5H | 169.72 |
| 11_20327 | 5H | 93.22 |
| 11_10256 | 7H | 79.08 |
| 11_10259 | 1H | 40.4 |
| 11_10260 | 5H | 38.78 |
| 11_10261 | 4H | 53.94 |
| 11_10262 | 4H | 63.44 |
| 11_20332 | 5H | 49.16 |
| 11_10265 | 2H | 80.14 |
| 11_20333 | 3H | 67.86 |
| 11_20334 | 5H | 159.97 |
| 11_20336 | 0 | 0 |
| 11_10269 | 4H | 138.7 |
| 11_10275 | 1H | 35.77 |
| 11_10276 | 3H | 86.57 |
| 11_20340 | 2H | 98.74 |
| 11_10279 | 1H | 77.57 |
| 11_20343 | 3H | 136.92 |
| 11_10280 | 3H | 136.92 |
| 11_10281 | 3H | 73.3 |

| | | |
|---|---|---|
| 11_10283 | 3H | 180.12 |
| 11_20349 | 7H | 86.84 |
| 11_20347 | 5H | 121.67 |
| 11_10287 | 2H | 98.74 |
| 11_20354 | 7H | 125.16 |
| 11_20355 | 6H | 120.69 |
| 11_20356 | 3H | 50.85 |
| 11_10292 | 5H | 139.63 |
| 11_20358 | 4H | 96.42 |
| 11_10293 | 1H | 48.99 |
| 11_10294 | 1H | 40.4 |
| 11_10299 | 7H | 72.18 |
| 11_20362 | 3H | 90.52 |
| 11_20365 | 7H | 162.24 |
| 11_10303 | 7H | 89.78 |
| 11_20366 | 2H | 145.69 |
| 11_10309 | 4H | 85.07 |
| 11_10310 | 5H | 183.14 |
| 11_10312 | 3H | 125.11 |
| 11_20372 | 5H | 50.53 |
| 11_20373 | 0 | 0 |
| 11_10315 | 2H | 158.18 |
| 11_20375 | 5H | 139 |
| 11_10317 | 2H | 68.56 |
| 11_10318 | 5H | 42.41 |
| 11_10319 | 4H | 11.04 |
| 11_10323 | 6H | 59.25 |
| 11_20379 | 6H | 115.31 |
| 11_20383 | 1H | 134.96 |
| 11_20384 | 4H | 99.36 |
| 11_20385 | 7H | 111.15 |
| 11_10325 | 2H | 56.64 |
| 11_10326 | 2H | 4.72 |
| 11_10329 | 2H | 168.26 |
| 11_20386 | 5H | 24.76 |
| 11_20387 | 2H | 59.58 |
| 11_10331 | 6H | 92.69 |
| 11_10332 | 1H | 11.66 |
| 11_20388 | 5H | 146.55 |
| 11_10334 | 4H | 113.95 |
| 11_10335 | 3H | 77.37 |
| 11_10336 | 5H | 157.61 |
| 11_10338 | 1H | 118.98 |
| 11_20390 | 2H | 72.99 |
| 11_20392 | 5H | 60.21 |
| 11_20394 | 2H | 26.63 |
| 11_10343 | 3H | 179.81 |
| 11_10342 | 2H | 44.82 |
| 11_10352 | 0 | 0 |

| | | |
|---|---|---|
| 11_10357 | 1H | 103.99 |
| 11_10360 | 5H | 121.67 |
| 11_10363 | 5H | 147.49 |
| 11_10365 | 3H | 65.25 |
| 11_20409 | 3H | 143.33 |
| 11_20410 | 3H | 39.92 |
| 11_20411 | 4H | 30.37 |
| 11_20412 | 4H | 64.64 |
| 11_10370 | 7H | 81.78 |
| 11_20415 | 6H | 15.81 |
| 11_10373 | 3H | 73.97 |
| 11_20419 | 2H | 85.52 |
| 11_10376 | 2H | 149.27 |
| 11_10379 | 4H | 58.2 |
| 11_20422 | 4H | 28 |
| 11_10380 | 3H | 63.25 |
| 11_10381 | 3H | 143.33 |
| 11_10383 | 2H | 147.37 |
| 11_20427 | 1H | 42.42 |
| 11_20428 | 3H | 67.86 |
| 11_10385 | 5H | 155.23 |
| 11_20432 | 1H | 62.05 |
| 11_10387 | 4H | 130.81 |
| 11_20434 | 1H | 89.77 |
| 11_20438 | 2H | 69.05 |
| 11_20439 | 3H | 67.27 |
| 11_20441 | 5H | 54.77 |
| 11_10390 | 6H | 137.41 |
| 11_10394 | 7H | 81.78 |
| 11_20444 | 3H | 68.51 |
| 11_10396 | 1H | 98.68 |
| 11_20450 | 4H | 64 |
| 11_20449 | 5H | 90.07 |
| 11_20451 | 4H | 79.47 |
| 11_10399 | 2H | 40.03 |
| 11_20452 | 7H | 146.27 |
| 11_10400 | 6H | 100.68 |
| 11_20453 | 4H | 71.71 |
| 11_10401 | 5H | 187.2 |
| 11_20454 | 4H | 111.13 |
| 11_20458 | 2H | 68.07 |
| 11_10404 | 2H | 133.71 |
| 11_10405 | 5H | 172.83 |
| 11_20460 | 7H | 82.2 |
| 11_20461 | 5H | 42.41 |
| 11_10409 | 4H | 3.2 |
| 11_10411 | 4H | 56.22 |
| 11_20467 | 6H | 118.15 |
| 11_10414 | 5H | 93.22 |

| | | |
|---|---|---|
| 11_20472 | 4H | 64 |
| 11_20475 | 1H | 93.48 |
| 11_20476 | 2H | 67.08 |
| 11_10419 | 1H | 4.71 |
| 11_20479 | 0 | 0 |
| 11_10422 | 2H | 56.64 |
| 11_20482 | 4H | 69.24 |
| 11_10424 | 4H | 56.22 |
| 11_20487 | 5H | 130.32 |
| 11_20485 | 7H | 91.67 |
| 11_20486 | 3H | 66.62 |
| 11_20488 | 6H | 84.47 |
| 11_10427 | 6H | 37.99 |
| 11_20493 | 6H | 1.03 |
| 11_20494 | 2H | 155.68 |
| 11_10429 | 2H | 130.38 |
| 11_10431 | 7H | 67.02 |
| 11_20495 | 7H | 18.73 |
| 11_10432 | 4H | 54.73 |
| 11_10433 | 1H | 95.14 |
| 11_10434 | 1H | 88.88 |
| 11_20498 | 2H | 116.5 |
| 11_20500 | 2H | 63.55 |
| 11_10436 | 2H | 72.99 |
| 11_10438 | 1H | 42.42 |
| 11_20501 | 5H | 51.51 |
| 11_20502 | 1H | 2.55 |
| 11_20504 | 7H | 158.84 |
| 11_10442 | 7H | 85.28 |
| 11_20507 | 7H | 27.09 |
| 11_20509 | 0 | 0 |
| 11_20511 | 2H | 137.03 |
| 11_10443 | 1H | 143.2 |
| 11_10444 | 3H | 98.05 |
| 11_10446 | 2H | 140.69 |
| 11_20513 | 0 | 0 |
| 11_20514 | 1H | 35.98 |
| 11_20515 | 4H | 112.22 |
| 11_20521 | 3H | 86.03 |
| 11_10451 | 7H | 27.09 |
| 11_20523 | 3H | 125.11 |
| 11_20524 | 5H | 42.41 |
| 11_20526 | 5H | 86.08 |
| 11_20527 | 3H | 142.17 |
| 11_20529 | 3H | 11.01 |
| 11_10454 | 7H | 139.9 |
| 11_10455 | 6H | 73.84 |
| 11_20531 | 6H | 111.08 |
| 11_10456 | 3H | 67.86 |

| | | |
|---|---|---|
| 11_20532 | 2H | 72.99 |
| 11_20533 | 5H | 10.08 |
| 11_20534 | 7H | 4.5 |
| 11_20536 | 5H | 168.44 |
| 11_20537 | 6H | 142.2 |
| 11_10460 | 1H | 3.21 |
| 11_10461 | 6H | 52.19 |
| 11_10462 | 6H | 49.17 |
| 11_20545 | 5H | 154.51 |
| 11_20546 | 5H | 163.72 |
| 11_20549 | 5H | 95.11 |
| 11_10471 | 1H | 89.77 |
| 11_20550 | 1H | 90.31 |
| 11_10466 | 1H | 84.71 |
| 11_20551 | 5H | 138.25 |
| 11_20552 | 3H | 26.23 |
| 11_10467 | 4H | 82.81 |
| 11_20553 | 5H | 1.91 |
| 11_20557 | 4H | 20.87 |
| 11_20558 | 6H | 122.14 |
| 11_10469 | 6H | 81.79 |
| 11_20560 | 5H | 156.27 |
| 11_10470 | 1H | 42.42 |
| 11_20561 | 2H | 169.66 |
| 11_20562 | 2H | 10.86 |
| 11_20563 | 2H | 10.07 |
| 11_10475 | 2H | 101.3 |
| 11_20568 | 5H | 143.29 |
| 11_10477 | 5H | 107.19 |
| 11_10480 | 4H | 58.2 |
| 11_20570 | 7H | 112.35 |
| 11_20571 | 5H | 38.78 |
| 11_10481 | 5H | 42.41 |
| 11_20572 | 6H | 59.25 |
| 11_20573 | 5H | 152.93 |
| 11_20577 | 6H | 80.06 |
| 11_20580 | 4H | 79.47 |
| 11_10490 | 4H | 12.34 |
| 11_20582 | 5H | 6.36 |
| 11_20583 | 3H | 66.62 |
| 11_20584 | 0 | 0 |
| 11_20585 | 2H | 72.03 |
| 11_10494 | 6H | 44.96 |
| 11_10496 | 0 | 0 |
| 11_10498 | 2H | 53.09 |
| 11_20590 | 2H | 154.65 |
| 11_20594 | 1H | 139.6 |
| 11_10509 | 4H | 56.22 |
| 11_20595 | 3H | 14.91 |

| | | |
|---|---|---|
| 11_10510 | 4H | 112.88 |
| 11_10513 | 6H | 60.65 |
| 11_20600 | 6H | 59.25 |
| 11_10515 | 3H | 110.09 |
| 11_10516 | 1H | 63.14 |
| 11_10518 | 5H | 88.05 |
| 11_20606 | 4H | 28 |
| 11_20607 | 3H | 32.92 |
| 11_10520 | 1H | 45.2 |
| 11_20609 | 0 | 0 |
| 11_20610 | 4H | 61.56 |
| 11_10522 | 1H | 101.34 |
| 11_10523 | 4H | 88.3 |
| 11_10525 | 2H | 38.86 |
| 11_10527 | 4H | 65.28 |
| 11_20612 | 3H | 139.66 |
| 11_10528 | 5H | 155.45 |
| 11_10531 | 7H | 85.28 |
| 11_10534 | 7H | 82.41 |
| 11_20617 | 1H | 27.12 |
| 11_10536 | 5H | 155.23 |
| 11_10538 | 2H | 133.04 |
| 11_10539 | 6H | 50.33 |
| 11_20620 | 6H | 78.52 |
| 11_20623 | 0 | 0 |
| 11_20625 | 1H | 110.08 |
| 11_20626 | 3H | 109.43 |
| 11_20628 | 3H | 108.7 |
| 11_20629 | 5H | 114.82 |
| 11_20631 | 2H | 67.08 |
| 11_10547 | 7H | 160.97 |
| 11_10551 | 2H | 156.77 |
| 11_10552 | 1H | 54.54 |
| 11_20636 | 6H | 77.53 |
| 11_20637 | 5H | 115.76 |
| 11_10557 | 5H | 144.6 |
| 11_10559 | 3H | 26.71 |
| 11_20639 | 3H | 71.26 |
| 11_10578 | 5H | 83.8 |
| 11_10563 | 7H | 112.99 |
| 11_10566 | 2H | 157.26 |
| 11_20642 | 1H | 62.6 |
| 11_10568 | 4H | 62.15 |
| 11_20644 | 5H | 164.15 |
| 11_20645 | 5H | 74.83 |
| 11_20646 | 5H | 157.61 |
| 11_20647 | 3H | 43.96 |
| 11_20650 | 3H | 135.43 |
| 11_10574 | 4H | 21.53 |

| | | |
|---|---|---|
| 11_20651 | 6H | 58.48 |
| 11_10576 | 7H | 35.36 |
| 11_20652 | 7H | 111.15 |
| 11_10577 | 4H | 54.73 |
| 11_20653 | 5H | 121.67 |
| 11_20654 | 6H | 95.7 |
| 11_20656 | 6H | 58.48 |
| 11_10580 | 5H | 29.9 |
| 11_10582 | 5H | 155.66 |
| 11_20659 | 3H | 102.12 |
| 11_20660 | 1H | 42.42 |
| 11_10584 | 3H | 127.26 |
| 11_20662 | 3H | 136.92 |
| 11_10586 | 1H | 123.1 |
| 11_20666 | 3H | 48.02 |
| 11_10588 | 4H | 97.52 |
| 11_20668 | 4H | 145.7 |
| 11_20669 | 2H | 68.56 |
| 11_10589 | 5H | 149.94 |
| 11_20670 | 4H | 90.11 |
| 11_20671 | 7H | 64.98 |
| 11_10590 | 1H | 141.46 |
| 11_20673 | 6H | 78.52 |
| 11_20674 | 2H | 55.46 |
| 11_10593 | 5H | 164.36 |
| 11_20676 | 5H | 141.88 |
| 11_20675 | 6H | 53.54 |
| 11_10595 | 6H | 107.49 |
| 11_10597 | 1H | 36.64 |
| 11_20680 | 4H | 28.75 |
| 11_10614 | 4H | 111.81 |
| 11_20681 | 2H | 169.66 |
| 11_20682 | 6H | 84.47 |
| 11_10602 | 2H | 63.55 |
| 11_20686 | 5H | 163.72 |
| 11_20687 | 6H | 139.09 |
| 11_20690 | 2H | 78.54 |
| 11_20691 | 7H | 143.13 |
| 11_10606 | 4H | 78.08 |
| 11_10608 | 6H | 88.25 |
| 11_20694 | 3H | 84.98 |
| 11_20695 | 3H | 87.8 |
| 11_10610 | 4H | 139.97 |
| 11_20697 | 5H | 41.87 |
| 11_10611 | 4H | 135.58 |
| 11_20698 | 1H | 42.42 |
| 11_20700 | 5H | 42.41 |
| 11_20701 | 4H | 133.52 |
| 11_10617 | 1H | 59.29 |

| | | |
|---|---|---|
| 11_20704 | 3H | 76.43 |
| 11_20707 | 6H | 49.67 |
| 11_20708 | 5H | 42.41 |
| 11_20709 | 6H | 74.65 |
| 11_10621 | 5H | 31.2 |
| 11_10619 | 2H | 95.58 |
| 11_20712 | 1H | 20.33 |
| 11_10624 | 2H | 67.08 |
| 11_20713 | 5H | 56.77 |
| 11_20714 | 6H | 75.28 |
| 11_10627 | 4H | 79.47 |
| 11_10625 | 2H | 156.77 |
| 11_20715 | 2H | 151.92 |
| 11_20719 | 3H | 52.17 |
| 11_20720 | 6H | 57.18 |
| 11_10630 | 2H | 117.59 |
| 11_10631 | 3H | 153.61 |
| 11_20722 | 7H | 13.7 |
| 11_20723 | 4H | 73.62 |
| 11_20724 | 2H | 14.98 |
| 11_20725 | 6H | 118.15 |
| 11_10632 | 2H | 73.35 |
| 11_10638 | 2H | 57.29 |
| 11_10639 | 4H | 76.09 |
| 11_20730 | 5H | 33.71 |
| 11_10641 | 5H | 59.72 |
| 11_20732 | 4H | 100.23 |
| 11_20733 | 6H | 122.74 |
| 11_20734 | 2H | 86.58 |
| 11_20736 | 5H | 72.68 |
| 11_20737 | 5H | 47.04 |
| 11_10644 | 1H | 128.63 |
| 11_10645 | 6H | 129.73 |
| 11_10646 | 3H | 167.33 |
| 11_20740 | 4H | 82.81 |
| 11_10648 | 2H | 42.78 |
| 11_10651 | 2H | 79.82 |
| 11_20742 | 3H | 20.52 |
| 11_20743 | 6H | 48.42 |
| 11_20744 | 6H | 80.86 |
| 11_10653 | 3H | 69.4 |
| 11_20746 | 6H | 85.94 |
| 11_10654 | 0 | 0 |
| 11_20748 | 2H | 61.49 |
| 11_10658 | 5H | 139 |
| 11_10656 | 2H | 145.69 |
| 11_20750 | 7H | 61.67 |
| 11_10659 | 6H | 59.25 |
| 11_20754 | 1H | 101.99 |

| | | |
|---|---|---|
| 11_10661 | 5H | 42.41 |
| 11_20758 | 7H | 28.16 |
| 11_20762 | 4H | 109 |
| 11_10667 | 4H | 54.73 |
| 11_10668 | 4H | 50.22 |
| 11_10669 | 6H | 2.84 |
| 11_20765 | 4H | 95.77 |
| 11_10671 | 5H | 50.53 |
| 11_20766 | 5H | 38.78 |
| 11_20769 | 1H | 92.43 |
| 11_20772 | 1H | 142.55 |
| 11_10673 | 7H | 84.3 |
| 11_10676 | 6H | 32.05 |
| 11_20777 | 4H | 29.76 |
| 11_20778 | 3H | 87.8 |
| 11_20780 | 1H | 108.52 |
| 11_10679 | 2H | 66.2 |
| 11_20782 | 4H | 54.94 |
| 11_20783 | 6H | 100.68 |
| 11_20784 | 6H | 80.06 |
| 11_10681 | 3H | 172.82 |
| 11_10682 | 7H | 0.63 |
| 11_20786 | 5H | 183.14 |
| 11_10685 | 2H | 72.99 |
| 11_10686 | 1H | 77.92 |
| 11_10687 | 7H | 139.9 |
| 11_20790 | 7H | 49.82 |
| 11_10688 | 5H | 28.98 |
| 11_20791 | 5H | 145.9 |
| 11_20792 | 1H | 92.43 |
| 11_20794 | 3H | 28.02 |
| 11_20795 | 5H | 95.52 |
| 11_20796 | 3H | 67.86 |
| 11_20797 | 3H | 5.36 |
| 11_20798 | 1H | 48.99 |
| 11_10692 | 2H | 73.35 |
| 11_20799 | 6H | 59.25 |
| 11_20801 | 3H | 66.62 |
| 11_10694 | 3H | 173.43 |
| 11_10697 | 4H | 135.58 |
| 11_10700 | 7H | 80.3 |
| 11_10702 | 3H | 169.95 |
| 11_20808 | 7H | 102.97 |
| 11_20810 | 1H | 46.5 |
| 11_10705 | 5H | 125.09 |
| 11_20815 | 4H | 85.07 |
| 11_20820 | 4H | 73.03 |
| 11_10707 | 2H | 131.69 |
| 11_20824 | 7H | 109.44 |

| | | |
|---|---|---|
| 11_10710 | 3H | 39.57 |
| 11_20829 | 5H | 160.9 |
| 11_20828 | 7H | 81.78 |
| 11_10712 | 4H | 132.78 |
| 11_20835 | 6H | 60.65 |
| 11_20838 | 4H | 107.14 |
| 11_20840 | 1H | 140.69 |
| 11_20841 | 5H | 41.45 |
| 11_20844 | 1H | 111.81 |
| 11_20845 | 5H | 33.55 |
| 11_10722 | 1H | 126.61 |
| 11_10723 | 4H | 93.08 |
| 11_10724 | 4H | 91.42 |
| 11_20885 | 7H | 75.52 |
| 11_20850 | 5H | 90.07 |
| 11_20851 | 3H | 148.79 |
| 11_20853 | 4H | 55.58 |
| 11_20854 | 6H | 59.25 |
| 11_20855 | 1H | 41.74 |
| 11_20856 | 3H | 67.86 |
| 11_10726 | 7H | 51.23 |
| 11_10728 | 3H | 72.21 |
| 11_20862 | 2H | 67.08 |
| 11_10729 | 1H | 116.24 |
| 11_20864 | 2H | 32.35 |
| 11_20866 | 3H | 63.94 |
| 11_20873 | 5H | 21.14 |
| 11_10731 | 2H | 124.98 |
| 11_10733 | 2H | 56.64 |
| 11_10734 | 6H | 109.05 |
| 11_20877 | 3H | 83.8 |
| 11_20879 | 7H | 81.78 |
| 11_10736 | 5H | 171.58 |
| 11_20882 | 6H | 3.46 |
| 11_20884 | 5H | 132.32 |
| 11_20886 | 6H | 1.64 |
| 11_20887 | 2H | 72.99 |
| 11_10741 | 5H | 155.45 |
| 11_20889 | 6H | 86.54 |
| 11_20890 | 3H | 65.25 |
| 11_20891 | 2H | 61.49 |
| 11_20892 | 6H | 79.17 |
| 11_10744 | 1H | 23.62 |
| 11_20893 | 7H | 81.78 |
| 11_20894 | 5H | 1.26 |
| 11_20895 | 2H | 149.27 |
| 11_20896 | 7H | 88.06 |
| 11_20897 | 5H | 177.9 |
| 11_10747 | 3H | 104.15 |

| | | |
|---|---|---|
| 11_10748 | 6H | 133.9 |
| 11_10749 | 6H | 59.25 |
| 11_10750 | 0 | 0 |
| 11_10751 | 4H | 97.05 |
| 11_20904 | 6H | 72.17 |
| 11_10753 | 3H | 123.37 |
| 11_10754 | 3H | 134.71 |
| 11_20906 | 4H | 77.87 |
| 11_20908 | 1H | 122.34 |
| 11_20909 | 1H | 105.77 |
| 11_10755 | 5H | 136.94 |
| 11_20911 | 7H | 81.78 |
| 11_20912 | 1H | 46.5 |
| 11_10756 | 4H | 54.73 |
| 11_20915 | 1H | 141.46 |
| 11_10757 | 1H | 22.04 |
| 11_20920 | 3H | 148.79 |
| 11_20921 | 1H | 109.38 |
| 11_10760 | 1H | 28.2 |
| 11_20924 | 4H | 75.44 |
| 11_10764 | 1H | 34.45 |
| 11_20931 | 3H | 81.28 |
| 11_10767 | 3H | 179.16 |
| 11_10768 | 1H | 52.63 |
| 11_10770 | 2H | 167.66 |
| 11_20934 | 5H | 159.55 |
| 11_10771 | 5H | 88.05 |
| 11_20936 | 6H | 46.44 |
| 11_10772 | 7H | 47.1 |
| 11_20939 | 4H | 51.53 |
| 11_10773 | 7H | 81.78 |
| 11_20943 | 2H | 163.19 |
| 11_20944 | 3H | 131.24 |
| 11_20946 | 6H | 59.25 |
| 11_20947 | 2H | 84.96 |
| 11_10778 | 5H | 166.65 |
| 11_20952 | 3H | 156.42 |
| 11_10780 | 2H | 137.03 |
| 11_20956 | 1H | 64.44 |
| 11_10781 | 6H | 72.17 |
| 11_20958 | 5H | 42.41 |
| 11_20959 | 1H | 119.78 |
| 11_10783 | 5H | 130.32 |
| 11_20960 | 2H | 85.73 |
| 11_20961 | 5H | 51.51 |
| 11_20962 | 7H | 152.41 |
| 11_10785 | 4H | 102.93 |
| 11_20968 | 3H | 29.75 |
| 11_10787 | 2H | 31.91 |

| | | |
|---|---|---|
| 11_20969 | 6H | 84.47 |
| 11_20970 | 3H | 67.86 |
| 11_20972 | 6H | 106.82 |
| 11_10789 | 1H | 114.06 |
| 11_20974 | 4H | 116.68 |
| 11_20975 | 7H | 62.43 |
| 11_10791 | 2H | 164.35 |
| 11_10793 | 4H | 50.22 |
| 11_20982 | 0 | 0 |
| 11_20980 | 5H | 33.55 |
| 11_20987 | 5H | 38.78 |
| 11_20988 | 5H | 158.28 |
| 11_10796 | 2H | 63.55 |
| 11_10797 | 7H | 135.86 |
| 11_10798 | 1H | 59.29 |
| 11_10799 | 6H | 35.32 |
| 11_20990 | 1H | 80.47 |
| 11_20993 | 7H | 29.7 |
| 11_20996 | 6H | 104.5 |
| 11_20994 | 2H | 160.46 |
| 11_20995 | 3H | 69.95 |
| 11_20998 | 0 | 0 |
| 11_20999 | 3H | 108.7 |
| 11_21000 | 1H | 45.85 |
| 11_21001 | 5H | 63.93 |
| 11_21005 | 2H | 54.92 |
| 11_21008 | 3H | 169.29 |
| 11_10805 | 5H | 122.33 |
| 11_21010 | 4H | 68.19 |
| 11_21012 | 5H | 167.4 |
| 11_21011 | 5H | 42.41 |
| 11_21014 | 6H | 58.48 |
| 11_21015 | 2H | 26 |
| 11_10809 | 4H | 63.79 |
| 11_21018 | 5H | 157.61 |
| 11_21025 | 6H | 102.04 |
| 11_21027 | 3H | 12.38 |
| 11_10814 | 1H | 28.99 |
| 11_10813 | 3H | 83.58 |
| 11_10815 | 6H | 97 |
| 11_21030 | 6H | 44.96 |
| 11_21032 | 6H | 11.68 |
| 11_10817 | 6H | 49.67 |
| 11_21035 | 4H | 134.55 |
| 11_10818 | 2H | 90.48 |
| 11_21038 | 1H | 122.34 |
| 11_21041 | 5H | 154.08 |
| 11_21040 | 5H | 42.41 |
| 11_10819 | 5H | 139 |

| | | |
|---|---|---|
| 11_10820 | 5H | 154.08 |
| 11_10821 | 3H | 134.71 |
| 11_21050 | 7H | 15.89 |
| 11_10823 | 2H | 95.58 |
| 11_21053 | 1H | 51.91 |
| 11_10825 | 3H | 39.92 |
| 11_10826 | 2H | 155.68 |
| 11_21061 | 5H | 99.39 |
| 11_21062 | 3H | 67.27 |
| 11_10829 | 4H | 83.75 |
| 11_21067 | 1H | 1.88 |
| 11_21069 | 6H | 71.32 |
| 11_21070 | 4H | 28 |
| 11_21071 | 4H | 54.94 |
| 11_21072 | 1H | 30.81 |
| 11_21073 | 4H | 54.73 |
| 11_10830 | 1H | 89.77 |
| 11_21077 | 5H | 143.29 |
| 11_21079 | 7H | 84.3 |
| 11_21083 | 3H | 111.49 |
| 11_10834 | 5H | 98.2 |
| 11_21087 | 4H | 71.71 |
| 11_21088 | 2H | 145.69 |
| 11_10837 | 2H | 42.01 |
| 11_10838 | 7H | 32.01 |
| 11_21093 | 3H | 59.83 |
| 11_10839 | 3H | 77.37 |
| 11_10840 | 5H | 50.53 |
| 11_10841 | 7H | 9.73 |
| 11_10842 | 3H | 139 |
| 11_21099 | 2H | 168.26 |
| 11_10843 | 7H | 145.6 |
| 11_10846 | 4H | 76.31 |
| 11_21104 | 7H | 134.1 |
| 11_10848 | 6H | 59.25 |
| 11_21109 | 3H | 63.25 |
| 11_21121 | 5H | 58.65 |
| 11_21110 | 2H | 76.61 |
| 11_10851 | 7H | 9.73 |
| 11_21111 | 4H | 113.95 |
| 11_21112 | 6H | 139.75 |
| 11_10853 | 7H | 111.15 |
| 11_10854 | 1H | 119.78 |
| 11_10855 | 5H | 132.32 |
| 11_10856 | 5H | 42.41 |
| 11_21120 | 3H | 75.27 |
| 11_21122 | 4H | 35.24 |
| 11_10861 | 7H | 134.1 |
| 11_10863 | 3H | 42.31 |

| | | |
|---|---|---|
| 11_21125 | 2H | 146.72 |
| 11_21126 | 1H | 78.58 |
| 11_21129 | 3H | 63.94 |
| 11_21133 | 5H | 67.66 |
| 11_10867 | 3H | 135.43 |
| 11_21134 | 1H | 37.3 |
| 11_10869 | 5H | 165.28 |
| 11_10870 | 5H | 168.67 |
| 11_21136 | 2H | 101.3 |
| 11_21138 | 5H | 170.25 |
| 11_21140 | 1H | 127.26 |
| 11_21142 | 0 | 0 |
| 11_21141 | 5H | 167.9 |
| 11_21144 | 2H | 78.54 |
| 11_21145 | 3H | 42.96 |
| 11_21147 | 3H | 67.27 |
| 11_21148 | 5H | 50.53 |
| 11_21150 | 5H | 83.17 |
| 11_21153 | 2H | 43.38 |
| 11_21155 | 5H | 182.53 |
| 11_21158 | 6H | 57.83 |
| 11_21161 | 3H | 122.7 |
| 11_21168 | 5H | 98.2 |
| 11_21174 | 1H | 8.96 |
| 11_21175 | 2H | 108.58 |
| 11_10882 | 6H | 44.96 |
| 11_21177 | 5H | 125.09 |
| 11_21181 | 2H | 155.68 |
| 11_21184 | 2H | 124.98 |
| 11_10885 | 7H | 138.83 |
| 11_21187 | 2H | 28.15 |
| 11_21188 | 1H | 42.42 |
| 11_10886 | 3H | 13.78 |
| 11_21189 | 3H | 52.17 |
| 11_21191 | 4H | 70.77 |
| 11_21192 | 1H | 89.77 |
| 11_21193 | 1H | 42.42 |
| 11_21197 | 3H | 63.25 |
| 11_10890 | 1H | 76.92 |
| 11_21200 | 5H | 51.51 |
| 11_10891 | 2H | 29.75 |
| 11_21201 | 7H | 98.95 |
| 11_21202 | 5H | 7.84 |
| 11_21203 | 5H | 121.67 |
| 11_10894 | 7H | 2.13 |
| 11_10896 | 7H | 150.44 |
| 11_21204 | 6H | 8.74 |
| 11_21205 | 2H | 82.98 |
| 11_21207 | 0 | 0 |

| | | |
|---|---|---|
| 11_21209 | 7H | 130.64 |
| 11_21210 | 4H | 138.7 |
| 11_10900 | 2H | 114.34 |
| 11_21212 | 3H | 120.73 |
| 11_21215 | 5H | 42.41 |
| 11_21216 | 6H | 59.25 |
| 11_21217 | 1H | 48.29 |
| 11_21220 | 2H | 136.33 |
| 11_10901 | 5H | 152.93 |
| 11_10902 | 5H | 155.45 |
| 11_21223 | 7H | 144.96 |
| 11_21224 | 6H | 86.96 |
| 11_21225 | 6H | 72.17 |
| 11_21226 | 1H | 9.37 |
| 11_10903 | 1H | 133.47 |
| 11_21228 | 4H | 3.8 |
| 11_21229 | 7H | 128.72 |
| 11_21238 | 2H | 125.97 |
| 11_21239 | 5H | 60.21 |
| 11_21241 | 5H | 131.64 |
| 11_10911 | 1H | 121.8 |
| 11_21242 | 2H | 94.25 |
| 11_10913 | 5H | 42.41 |
| 11_10914 | 4H | 65.28 |
| 11_21244 | 5H | 51.51 |
| 11_10916 | 2H | 134.35 |
| 11_10918 | 3H | 132.62 |
| 11_10919 | 2H | 42.01 |
| 11_10920 | 7H | 26.14 |
| 11_21247 | 5H | 125.09 |
| 11_21250 | 2H | 159.62 |
| 11_21251 | 2H | 82.44 |
| 11_21253 | 5H | 36.72 |
| 11_21254 | 4H | 56.22 |
| 11_21256 | 6H | 80.86 |
| 11_10924 | 7H | 80.64 |
| 11_10925 | 3H | 66.62 |
| 11_10926 | 3H | 67.86 |
| 11_21258 | 2H | 80.36 |
| 11_21260 | 5H | 49.16 |
| 11_21261 | 2H | 27.5 |
| 11_21265 | 2H | 27.5 |
| 11_21267 | 3H | 174.45 |
| 11_21271 | 6H | 118.15 |
| 11_10933 | 1H | 45.2 |
| 11_21270 | 7H | 62.64 |
| 11_21272 | 3H | 157.77 |
| 11_21274 | 2H | 154.39 |
| 11_21275 | 5H | 57.89 |

| | | |
|---|---|---|
| 11_10939 | 6H | 37.29 |
| 11_21277 | 3H | 124.04 |
| 11_10942 | 4H | 55.15 |
| 11_21280 | 7H | 141.15 |
| 11_21281 | 6H | 47.1 |
| 11_10943 | 2H | 17.24 |
| 11_21286 | 2H | 66.2 |
| 11_10946 | 4H | 59.06 |
| 11_10947 | 2H | 67.08 |
| 11_21289 | 5H | 136.94 |
| 11_21293 | 6H | 72.17 |
| 11_21294 | 3H | 100.29 |
| 11_21296 | 4H | 71.71 |
| 11_21297 | 5H | 145.9 |
| 11_21298 | 6H | 70.5 |
| 11_21299 | 2H | 163.19 |
| 11_21302 | 7H | 81.78 |
| 11_10954 | 6H | 59.25 |
| 11_10956 | 7H | 27.69 |
| 11_21304 | 2H | 34.24 |
| 11_10957 | 1H | 41.74 |
| 11_21305 | 3H | 80.49 |
| 11_21307 | 7H | 1.71 |
| 11_21308 | 5H | 41.45 |
| 11_21309 | 5H | 64.77 |
| 11_21310 | 6H | 65.83 |
| 11_21312 | 1H | 45.2 |
| 11_21314 | 5H | 96.12 |
| 11_21315 | 2H | 138.04 |
| 11_21318 | 5H | 44.23 |
| 11_21321 | 5H | 97.49 |
| 11_10965 | 7H | 24.15 |
| 11_21324 | 5H | 25.42 |
| 11_10966 | 3H | 65.25 |
| 11_21325 | 5H | 123.12 |
| 11_21326 | 7H | 42.26 |
| 11_10971 | 7H | 0 |
| 11_10974 | 5H | 21.68 |
| 11_21333 | 1H | 52.63 |
| 11_10978 | 6H | 106.15 |
| 11_21339 | 6H | 65.29 |
| 11_21340 | 2H | 116.5 |
| 11_21344 | 5H | 53.84 |
| 11_10983 | 7H | 73.54 |
| 11_21346 | 2H | 161.3 |
| 11_21348 | 3H | 98.66 |
| 11_21350 | 5H | 42.41 |
| 11_21353 | 4H | 86.01 |
| 11_10988 | 2H | 124.98 |

| | | |
|---|---|---|
| 11_21354 | 1H | 1.68 |
| 11_10989 | 2H | 125.97 |
| 11_21355 | 5H | 149.94 |
| 11_21357 | 1H | 44.59 |
| 11_21358 | 3H | 93.81 |
| 11_21359 | 4H | 20.44 |
| 11_21361 | 1H | 48.99 |
| 11_21363 | 7H | 145.6 |
| 11_21366 | 2H | 27.5 |
| 11_21370 | 2H | 143.18 |
| 11_10994 | 6H | 34.66 |
| 11_10995 | 5H | 42.41 |
| 11_21373 | 1H | 96.95 |
| 11_10996 | 2H | 58.5 |
| 11_10997 | 2H | 62.85 |
| 11_21374 | 4H | 30.37 |
| 11_10999 | 7H | 157.44 |
| 11_21377 | 2H | 9.12 |
| 11_21381 | 3H | 112.15 |
| 11_11002 | 3H | 48.02 |
| 11_21384 | 1H | 138.86 |
| 11_21385 | 4H | 26.2 |
| 11_21390 | 5H | 106.36 |
| 11_21391 | 5H | 33.55 |
| 11_21392 | 1H | 114.06 |
| 11_11004 | 4H | 86.66 |
| 11_21397 | 4H | 35.24 |
| 11_21398 | 3H | 11.01 |
| 11_21399 | 2H | 72.99 |
| 11_21401 | 5H | 40.14 |
| 11_21402 | 3H | 56.54 |
| 11_21405 | 3H | 132.62 |
| 11_21406 | 2H | 142.67 |
| 11_11012 | 7H | 149.31 |
| 11_21409 | 7H | 89.12 |
| 11_21414 | 0 | 0 |
| 11_11014 | 7H | 54.32 |
| 11_11015 | 2H | 60.89 |
| 11_11016 | 3H | 71.26 |
| 11_11019 | 4H | 146.48 |
| 11_21421 | 5H | 91.98 |
| 11_21419 | 7H | 0 |
| 11_21425 | 6H | 133.9 |
| 11_21427 | 3H | 150.11 |
| 11_21428 | 3H | 143.33 |
| 11_21431 | 1H | 62.6 |
| 11_21435 | 3H | 66.62 |
| 11_21436 | 2H | 164.35 |
| 11_21437 | 7H | 11.65 |

| | | |
|---|---|---|
| 11_11023 | 2H | 158.6 |
| 11_21438 | 3H | 108.7 |
| 11_21440 | 2H | 140.69 |
| 11_11024 | 5H | 121.67 |
| 11_21443 | 7H | 0.84 |
| 11_21445 | 5H | 74.83 |
| 11_21447 | 5H | 41.45 |
| 11_21448 | 7H | 98.95 |
| 11_11028 | 7H | 65.98 |
| 11_11031 | 7H | 5.96 |
| 11_21452 | 5H | 155.23 |
| 11_21453 | 2H | 168.26 |
| 11_11038 | 1H | 132.16 |
| 11_11042 | 4H | 56.22 |
| 11_11043 | 2H | 125.97 |
| 11_11046 | 2H | 66.2 |
| 11_11048 | 5H | 24.76 |
| 11_21459 | 2H | 143.18 |
| 11_11049 | 1H | 59.29 |
| 11_11054 | 2H | 53.18 |
| 11_11058 | 2H | 102.38 |
| 11_11066 | 4H | 134.55 |
| 11_11067 | 6H | 62.71 |
| 11_11071 | 5H | 136.94 |
| 11_11072 | 2H | 77.76 |
| 11_11075 | 1H | 75.48 |
| 11_21467 | 6H | 128.41 |
| 11_11080 | 5H | 130.98 |
| 11_21469 | 6H | 76.03 |
| 11_11086 | 3H | 64.6 |
| 11_11090 | 5H | 125.09 |
| 11_21472 | 3H | 66.62 |
| 11_11094 | 2H | 119.72 |
| 11_21473 | 6H | 57.18 |
| 11_11098 | 7H | 64.98 |
| 11_11099 | 3H | 67.27 |
| 11_11105 | 1H | 138.86 |
| 11_11111 | 6H | 139.09 |
| 11_11114 | 4H | 59.8 |
| 11_11118 | 2H | 128.87 |
| 11_11122 | 7H | 79.79 |
| 11_11124 | 3H | 67.86 |
| 11_11125 | 3H | 66.62 |
| 11_11128 | 5H | 47.47 |
| 11_11132 | 7H | 0.63 |
| 11_11136 | 4H | 24.71 |
| 11_21481 | 4H | 62.81 |
| 11_11141 | 3H | 137.74 |
| 11_11147 | 6H | 95.7 |

| | | |
|---|---|---|
| 11_11153 | 6H | 59.91 |
| 11_11159 | 5H | 52.86 |
| 11_11162 | 1H | 45.2 |
| 11_11178 | 2H | 68.07 |
| 11_11179 | 7H | 4.5 |
| 11_11180 | 4H | 48.77 |
| 11_11185 | 5H | 154.08 |
| 11_11186 | 4H | 144.4 |
| 11_11187 | 6H | 131.64 |
| 11_11191 | 3H | 75.27 |
| 11_11196 | 3H | 138.39 |
| 11_11198 | 5H | 40.14 |
| 11_11199 | 4H | 23.96 |
| 11_11200 | 5H | 109.48 |
| 11_11205 | 6H | 53.54 |
| 11_11206 | 2H | 69.05 |
| 11_11207 | 4H | 72.36 |
| 11_11211 | 2H | 67.08 |
| 11_11213 | 4H | 95.22 |
| 11_11216 | 5H | 162.98 |
| 11_11219 | 7H | 82.2 |
| 11_11221 | 5H | 51.51 |
| 11_11223 | 1H | 1.24 |
| 11_11224 | 4H | 78.5 |
| 11_11227 | 2H | 149.27 |
| 11_11229 | 4H | 78.08 |
| 11_11236 | 2H | 127.74 |
| 11_11239 | 7H | 85.28 |
| 11_11240 | 5H | 51.51 |
| 11_11241 | 3H | 84.22 |
| 11_11243 | 7H | 121.36 |
| 11_11244 | 4H | 61.56 |
| 11_11246 | 6H | 93.44 |
| 11_21491 | 7H | 41.51 |
| 11_11249 | 5H | 60.21 |
| 11_21493 | 3H | 115.21 |
| 11_11256 | 1H | 49.65 |
| 11_11258 | 3H | 63.94 |
| 11_21494 | 7H | 81.78 |
| 11_11260 | 5H | 43.15 |
| 11_11262 | 2H | 156.77 |
| 11_21495 | 3H | 114.36 |
| 11_11273 | 5H | 105.69 |
| 11_11275 | 7H | 151.76 |
| 11_11276 | 0 | 0 |
| 11_11277 | 1H | 100.4 |
| 11_11281 | 5H | 54.77 |
| 11_11283 | 3H | 66.62 |
| 11_11287 | 1H | 43.08 |

| | | |
|---|---|---|
| 11_11290 | 5H | 83.17 |
| 11_11292 | 4H | 109.65 |
| 11_11294 | 6H | 105.05 |
| 11_11302 | 2H | 56.64 |
| 11_11307 | 2H | 109.29 |
| 11_11312 | 6H | 59.25 |
| 11_11314 | 3H | 83.16 |
| 11_11323 | 2H | 118.39 |
| 11_11329 | 6H | 78.52 |
| 11_11330 | 3H | 129.83 |
| 11_11332 | 4H | 56.22 |
| 11_11336 | 1H | 42.42 |
| 11_11341 | 5H | 108.28 |
| 11_21502 | 3H | 76.43 |
| 11_11345 | 4H | 5.23 |
| 11_11346 | 2H | 114.34 |
| 11_11348 | 7H | 64.98 |
| 11_11349 | 6H | 80.06 |
| 11_11350 | 5H | 93.66 |
| 11_21504 | 4H | 80.73 |
| 11_11354 | 2H | 68.07 |
| 11_11355 | 5H | 77.75 |
| 11_11361 | 0 | 0 |
| 11_11365 | 2H | 138.04 |
| 11_11367 | 1H | 64.44 |
| 11_11375 | 5H | 123.12 |
| 11_21507 | 0 | 0 |
| 11_11380 | 2H | 160.46 |
| 11_11381 | 5H | 8.25 |
| 11_11384 | 2H | 69.79 |
| 11_21509 | 6H | 64.65 |
| 11_21511 | 3H | 71.26 |
| 11_11391 | 3H | 77.37 |
| 11_11394 | 3H | 81.28 |
| 11_11398 | 4H | 97.05 |
| 11_11400 | 2H | 58.5 |
| 11_21514 | 5H | 147.49 |
| 11_11405 | 4H | 53.28 |
| 11_11406 | 6H | 6.54 |
| 11_11431 | 4H | 76.31 |
| 11_21516 | 7H | 0.21 |
| 11_11432 | 5H | 38.78 |
| 11_11435 | 2H | 89.68 |
| 11_11436 | 3H | 163.49 |
| 11_11440 | 7H | 146.27 |
| 11_11441 | 5H | 143.29 |
| 11_11445 | 7H | 85.28 |
| 11_21521 | 6H | 2.24 |
| 11_11448 | 5H | 131.64 |

| | | |
|---|---|---|
| 11_11453 | 3H | 8.56 |
| 11_11456 | 5H | 120.35 |
| 11_11458 | 6H | 88.88 |
| 11_11459 | 6H | 80.86 |
| 11_11461 | 7H | 83.42 |
| 11_11464 | 5H | 158.28 |
| 11_21523 | 3H | 169.95 |
| 11_11469 | 5H | 42.41 |
| 11_11470 | 4H | 111.13 |
| 11_11473 | 5H | 89.37 |
| 11_11478 | 1H | 42.42 |
| 11_11479 | 6H | 15.16 |
| 11_11480 | 2H | 118.39 |
| 11_21528 | 7H | 38.99 |
| 11_11481 | 1H | 128.04 |
| 11_11483 | 6H | 70.5 |
| 11_11488 | 6H | 133.9 |
| 11_11489 | 0 | 0 |
| 11_11490 | 5H | 150.29 |
| 11_11495 | 7H | 6.79 |
| 11_11497 | 5H | 151.35 |
| 11_11500 | 4H | 89.36 |
| 11_11501 | 3H | 67.86 |
| 11_11502 | 3H | 67.86 |
| 11_11503 | 3H | 125.11 |
| 11_11505 | 2H | 51.87 |
| 11_11507 | 5H | 118.16 |
| 11_11509 | 1H | 138.86 |
| 11_11513 | 4H | 80.73 |
| 11_21533 | 3H | 47.37 |
| 11_11516 | 3H | 174.99 |
| 11_11521 | 7H | 127.74 |
| 11_11522 | 2H | 58.15 |
| 11_21536 | 5H | 46.38 |
| 11_11528 | 1H | 122.34 |
| 11_11530 | 3H | 66.62 |
| 11_11533 | 2H | 100.05 |
| 11_11534 | 6H | 122.74 |
| 12_10014 | 3H | 173.43 |
| 12_10016 | 5H | 149.94 |
| 12_10022 | 4H | 95.27 |
| 12_10032 | 0 | 0 |
| 12_10034 | 5H | 51.51 |
| 12_10053 | 4H | 76.31 |
| 12_10063 | 4H | 44.99 |
| 12_10071 | 6H | 130.38 |
| 12_10077 | 5H | 95.11 |
| 12_10088 | 4H | 63.79 |
| 12_10089 | 7H | 91.12 |

| | | |
|---|---|---|
| 12_10099 | 2H | 68.07 |
| 12_10100 | 3H | 125.11 |
| 12_10103 | 3H | 3.97 |
| 12_10105 | 0 | 0 |
| 12_10122 | 3H | 143.33 |
| 12_10125 | 7H | 83.42 |
| 12_10149 | 0 | 0 |
| 12_10151 | 0 | 0 |
| 12_10154 | 2H | 69.05 |
| 12_10155 | 3H | 67.86 |
| 12_10159 | 1H | 42.42 |
| 12_10166 | 1H | 71.81 |
| 12_10170 | 4H | 88.7 |
| 12_10171 | 4H | 43.72 |
| 12_10179 | 0 | 0 |
| 12_10181 | 2H | 160.46 |
| 12_10188 | 3H | 137.74 |
| 12_10195 | 4H | 56.22 |
| 12_10198 | 1H | 51.2 |
| 12_10199 | 6H | 49.67 |
| 12_10201 | 1H | 58.59 |
| 12_10203 | 5H | 59.72 |
| 12_10205 | 3H | 141.22 |
| 12_10207 | 1H | 128.04 |
| 12_10210 | 0 | 0 |
| 12_10218 | 7H | 32.13 |
| 12_10219 | 0 | 0 |
| 12_10228 | 5H | 121.67 |
| 12_10230 | 2H | 7.92 |
| 12_10235 | 1H | 40.4 |
| 12_10241 | 7H | 112.99 |
| 12_10257 | 0 | 0 |
| 12_10264 | 5H | 47.04 |
| 12_10267 | 7H | 61.67 |
| 12_10268 | 7H | 81.78 |
| 12_10271 | 4H | 102.93 |
| 12_10273 | 5H | 157.61 |
| 12_10278 | 6H | 60.65 |
| 12_10284 | 0 | 0 |
| 12_10300 | 1H | 42.42 |
| 12_10306 | 0 | 0 |
| 12_10308 | 0 | 0 |
| 12_10313 | 0 | 0 |
| 12_10314 | 1H | 38.36 |
| 12_10322 | 5H | 189.22 |
| 12_10333 | 5H | 147.49 |
| 12_10337 | 0 | 0 |
| 12_10344 | 3H | 114.36 |
| 12_10345 | 6H | 60.65 |

| | | |
|---|---|---|
| 12_10347 | 4H | 43.72 |
| 12_10348 | 0 | 0 |
| 12_10362 | 7H | 110.11 |
| 12_10367 | 0 | 0 |
| 12_10368 | 7H | 38.57 |
| 12_10371 | 4H | 44.33 |
| 12_10374 | 0 | 0 |
| 12_10375 | 7H | 4.5 |
| 12_10378 | 7H | 162.03 |
| 12_10392 | 6H | 72.17 |
| 12_10393 | 0 | 0 |
| 12_10395 | 4H | 30.37 |
| 12_10403 | 7H | 55.64 |
| 12_10406 | 7H | 7.33 |
| 12_10408 | 5H | 90.07 |
| 12_10410 | 1H | 4.71 |
| 12_10420 | 1H | 0.54 |
| 12_10426 | 4H | 62.81 |
| 12_10430 | 0 | 0 |
| 12_10452 | 3H | 85.4 |
| 12_10459 | 7H | 81.78 |
| 12_10472 | 2H | 147.37 |
| 12_10474 | 2H | 67.63 |
| 12_10485 | 2H | 63.55 |
| 12_10486 | 2H | 49.5 |
| 12_10487 | 2H | 159.62 |
| 12_10489 | 0 | 0 |
| 12_10491 | 0 | 0 |
| 12_10493 | 0 | 0 |
| 12_10497 | 6H | 56.06 |
| 12_10499 | 5H | 28.98 |
| 12_10502 | 2H | 10.86 |
| 12_10505 | 3H | 137.74 |
| 12_10508 | 5H | 47.04 |
| 12_10535 | 1H | 91.73 |
| 12_10543 | 7H | 121.36 |
| 12_10545 | 2H | 77.76 |
| 12_10554 | 6H | 23.62 |
| 12_10562 | 4H | 26.2 |
| 12_10564 | 0 | 0 |
| 12_10571 | 3H | 18.21 |
| 12_10579 | 2H | 149.93 |
| 12_10581 | 7H | 82.41 |
| 12_10583 | 3H | 102.66 |
| 12_10591 | 6H | 59.25 |
| 12_10596 | 6H | 78.52 |
| 12_10605 | 7H | 58.53 |
| 12_10609 | 3H | 87.8 |
| 12_10613 | 0 | 0 |

| | | |
|---|---|---|
| 12_10623 | 0 | 0 |
| 12_10629 | 3H | 172.82 |
| 12_10633 | 5H | 69.29 |
| 12_10634 | 5H | 68.21 |
| 12_10636 | 1H | 5.41 |
| 12_10637 | 4H | 62.81 |
| 12_10640 | 2H | 72.03 |
| 12_10649 | 2H | 108.58 |
| 12_10650 | 2H | 84.96 |
| 12_10652 | 7H | 112.99 |
| 12_10657 | 7H | 61.67 |
| 12_10662 | 3H | 114.36 |
| 12_10666 | 4H | 109.65 |
| 12_10670 | 4H | 94.07 |
| 12_10674 | 5H | 80.18 |
| 12_10677 | 7H | 142.46 |
| 12_10678 | 3H | 71.26 |
| 12_10680 | 3H | 128.01 |
| 12_10689 | 0 | 0 |
| 12_10693 | 1H | 130.42 |
| 12_10698 | 7H | 81.78 |
| 12_10704 | 6H | 117.51 |
| 12_10717 | 2H | 82.98 |
| 12_10718 | 2H | 3.1 |
| 12_10725 | 5H | 52.86 |
| 12_10732 | 5H | 187.2 |
| 12_10735 | 0 | 0 |
| 12_10739 | 2H | 134.29 |
| 12_10746 | 1H | 141.46 |
| 12_10752 | 5H | 103.74 |
| 12_10758 | 6H | 65.83 |
| 12_10766 | 2H | 158.18 |
| 12_10769 | 5H | 163.72 |
| 12_10803 | 6H | 63.71 |
| 12_10808 | 1H | 129.71 |
| 12_10810 | 4H | 37.88 |
| 12_10811 | 6H | 49.67 |
| 12_10824 | 4H | 102.93 |
| 12_10836 | 0 | 0 |
| 12_10844 | 5H | 97.49 |
| 12_10847 | 2H | 34.24 |
| 12_10857 | 5H | 188.7 |
| 12_10859 | 2H | 93.26 |
| 12_10860 | 4H | 37.88 |
| 12_10864 | 5H | 35.39 |
| 12_10878 | 0 | 0 |
| 12_10880 | 0 | 0 |
| 12_10887 | 0 | 0 |
| 12_10888 | 7H | 157.85 |

| | | |
|---|---|---|
| 12_10897 | 0 | 0 |
| 12_10899 | 5H | 51.51 |
| 12_10904 | 5H | 147.49 |
| 12_10905 | 1H | 116.16 |
| 12_10915 | 2H | 154.59 |
| 12_10923 | 5H | 40.14 |
| 12_10927 | 2H | 61.49 |
| 12_10930 | 5H | 83.17 |
| 12_10937 | 2H | 166.6 |
| 12_10938 | 1H | 44.59 |
| 12_10948 | 2H | 63.55 |
| 12_10950 | 2H | 159.62 |
| 12_10953 | 5H | 63.93 |
| 12_10959 | 7H | 52.52 |
| 12_10968 | 3H | 38.91 |
| 12_10969 | 2H | 102.38 |
| 12_10970 | 2H | 0 |
| 12_10973 | 7H | 137.31 |
| 12_10979 | 7H | 36.92 |
| 12_10981 | 0 | 0 |
| 12_10982 | 7H | 83.42 |
| 12_11011 | 1H | 6.98 |
| 12_11030 | 2H | 6.09 |
| 12_11035 | 7H | 6.71 |
| 12_11039 | 0 | 0 |
| 12_11044 | 7H | 97.65 |
| 12_11047 | 3H | 150.11 |
| 12_11051 | 7H | 105.73 |
| 12_11055 | 7H | 85.28 |
| 12_11062 | 1H | 64.44 |
| 12_11063 | 4H | 56.22 |
| 12_11069 | 3H | 53.2 |
| 12_11077 | 0 | 0 |
| 12_11078 | 0 | 0 |
| 12_11084 | 3H | 75.27 |
| 12_11091 | 7H | 85.28 |
| 12_11095 | 0 | 0 |
| 12_11096 | 2H | 79.92 |
| 12_11103 | 7H | 70.21 |
| 12_11104 | 6H | 56.06 |
| 12_11106 | 5H | 83.17 |
| 12_11107 | 1H | 47.21 |
| 12_11108 | 4H | 65.28 |
| 12_11119 | 2H | 10.86 |
| 12_11121 | 2H | 76.61 |
| 12_11131 | 2H | 57.29 |
| 12_11137 | 0 | 0 |
| 12_11138 | 3H | 98.05 |
| 12_11139 | 4H | 111.81 |

| | | |
|---|---|---|
| 12_11140 | 6H | 59.91 |
| 12_11144 | 1H | 89.77 |
| 12_11146 | 7H | 81.78 |
| 12_11150 | 3H | 75.27 |
| 12_11151 | 5H | 51.51 |
| 12_11154 | 3H | 147.57 |
| 12_11155 | 2H | 66.2 |
| 12_11164 | 0 | 0 |
| 12_11171 | 6H | 53.54 |
| 12_11173 | 1H | 103.99 |
| 12_11175 | 4H | 26.71 |
| 12_11177 | 0 | 0 |
| 12_11181 | 6H | 62.71 |
| 12_11183 | 4H | 111.81 |
| 12_11184 | 7H | 118.44 |
| 12_11190 | 4H | 56.22 |
| 12_11192 | 5H | 186.66 |
| 12_11194 | 4H | 117.61 |
| 12_11208 | 0 | 0 |
| 12_11217 | 1H | 48.29 |
| 12_11235 | 4H | 133.52 |
| 12_11237 | 3H | 29.09 |
| 12_11245 | 5H | 113.51 |
| 12_11253 | 6H | 62.71 |
| 12_11254 | 0 | 0 |
| 12_11255 | 0 | 0 |
| 12_11267 | 1H | 76.22 |
| 12_11269 | 0 | 0 |
| 12_11271 | 1H | 136.7 |
| 12_11274 | 0 | 0 |
| 12_11278 | 2H | 73.89 |
| 12_11279 | 7H | 129.32 |
| 12_11285 | 2H | 108.58 |
| 12_11288 | 2H | 67.08 |
| 12_11295 | 3H | 67.27 |
| 12_11297 | 3H | 156.46 |
| 12_11300 | 4H | 12.34 |
| 12_11301 | 1H | 41.74 |
| 12_11309 | 7H | 151.1 |
| 12_11310 | 3H | 13.13 |
| 12_11311 | 1H | 3.21 |
| 12_11315 | 0 | 0 |
| 12_11316 | 2H | 73.89 |
| 12_11318 | 5H | 34.69 |
| 12_11321 | 6H | 65.83 |
| 12_11322 | 0 | 0 |
| 12_11324 | 2H | 72.99 |
| 12_11325 | 5H | 157.61 |
| 12_11353 | 6H | 56.06 |

| | | |
|---|---|---|
| 12_11357 | 1H | 30.15 |
| 12_11368 | 2H | 160.46 |
| 12_11377 | 7H | 84.3 |
| 12_11382 | 4H | 42.22 |
| 12_11383 | 0 | 0 |
| 12_11385 | 5H | 52.86 |
| 12_11386 | 0 | 0 |
| 12_11399 | 5H | 42.41 |
| 12_11408 | 0 | 0 |
| 12_11409 | 1H | 117.24 |
| 12_11413 | 5H | 187.2 |
| 12_11414 | 3H | 34.15 |
| 12_11429 | 3H | 71.26 |
| 12_11433 | 7H | 7.33 |
| 12_11434 | 3H | 6.31 |
| 12_11437 | 7H | 94.34 |
| 12_11443 | 1H | 125.29 |
| 12_11444 | 1H | 54.54 |
| 12_11449 | 2H | 86.58 |
| 12_11450 | 5H | 169.14 |
| 12_11452 | 2H | 33.82 |
| 12_11454 | 3H | 90.1 |
| 12_11455 | 6H | 44.96 |
| 12_11462 | 5H | 42.41 |
| 12_11463 | 1H | 88.88 |
| 12_11466 | 2H | 114.34 |
| 12_11468 | 0 | 0 |
| 12_11472 | 5H | 123.98 |
| 12_11475 | 6H | 70.5 |
| 12_11485 | 4H | 10.77 |
| 12_11487 | 6H | 59.25 |
| 12_11492 | 0 | 0 |
| 12_11494 | 6H | 111.74 |
| 12_11498 | 1H | 34.45 |
| 12_11499 | 7H | 85.28 |
| 12_11510 | 3H | 162.17 |
| 12_11511 | 3H | 61.94 |
| 12_11512 | 5H | 51.51 |
| 12_11517 | 3H | 93.81 |
| 12_11525 | 7H | 61.67 |
| 12_11526 | 4H | 90.1 |
| 12_11529 | 7H | 85.28 |
| 12_11535 | 5H | 121.67 |
| 12_11536 | 7H | 81.78 |
| 12_20006 | 0 | 0 |
| 12_20016 | 7H | 0 |
| 12_20027 | 2H | 155.68 |
| 12_20031 | 7H | 48.06 |
| 12_20045 | 5H | 119.65 |

| | | |
|---|---|---|
| 12_20059 | 5H | 45.64 |
| 12_20079 | 7H | 156.13 |
| 12_20090 | 3H | 3.97 |
| 12_20108 | 3H | 69.4 |
| 12_20142 | 6H | 59.25 |
| 12_20143 | 4H | 86.01 |
| 12_20158 | 0 | 0 |
| 12_20183 | 2H | 145.03 |
| 12_20187 | 1H | 105.77 |
| 12_20196 | 2H | 67.08 |
| 12_20201 | 7H | 2.13 |
| 12_20217 | 7H | 120.52 |
| 12_20227 | 7H | 8.45 |
| 12_20234 | 0 | 0 |
| 12_20235 | 2H | 61.49 |
| 12_20237 | 4H | 130.81 |
| 12_20241 | 7H | 142.46 |
| 12_20257 | 0 | 0 |
| 12_20274 | 4H | 6.86 |
| 12_20278 | 5H | 59.72 |
| 12_20285 | 0 | 0 |
| 12_20295 | 0 | 0 |
| 12_20297 | 5H | 63.33 |
| 12_20323 | 0 | 0 |
| 12_20326 | 2H | 42.01 |
| 12_20345 | 3H | 180.12 |
| 12_20350 | 5H | 61.88 |
| 12_20368 | 2H | 30.42 |
| 12_20369 | 3H | 138.39 |
| 12_20381 | 6H | 56.06 |
| 12_20403 | 5H | 94.4 |
| 12_20413 | 3H | 135.43 |
| 12_20416 | 0 | 0 |
| 12_20421 | 3H | 162.17 |
| 12_20424 | 0 | 0 |
| 12_20429 | 1H | 143.2 |
| 12_20436 | 0 | 0 |
| 12_20448 | 6H | 121.44 |
| 12_20489 | 2H | 90.48 |
| 12_20505 | 3H | 162.83 |
| 12_20574 | 3H | 67.86 |
| 12_20591 | 3H | 67.27 |
| 12_20593 | 2H | 29.05 |
| 12_20611 | 7H | 104.32 |
| 12_20613 | 1H | 105.77 |
| 12_20632 | 0 | 0 |
| 12_20640 | 7H | 148.28 |
| 12_20641 | 0 | 0 |
| 12_20649 | 0 | 0 |

| | | |
|---|---|---|
| 12_20684 | 7H | 109.99 |
| 12_20685 | 7H | 94.34 |
| 12_20688 | 2H | 55.46 |
| 12_20760 | 4H | 138.7 |
| 12_20770 | 5H | 35.88 |
| 12_20775 | 0 | 0 |
| 12_20793 | 2H | 103.13 |
| 12_20818 | 5H | 64.79 |
| 12_20825 | 0 | 0 |
| 12_20830 | 5H | 142.54 |
| 12_20831 | 4H | 56.22 |
| 12_20849 | 3H | 92.73 |
| 12_20863 | 3H | 73.3 |
| 12_20867 | 5H | 167.4 |
| 12_20917 | 2H | 61.49 |
| 12_20949 | 7H | 142.46 |
| 12_20954 | 0 | 0 |
| 12_20981 | 5H | 51.51 |
| 12_20985 | 0 | 0 |
| 12_20989 | 2H | 130.38 |
| 12_21003 | 0 | 0 |
| 12_21009 | 5H | 168.44 |
| 12_21019 | 0 | 0 |
| 12_21036 | 5H | 57.04 |
| 12_21049 | 2H | 31.17 |
| 12_21114 | 6H | 60.65 |
| 12_21115 | 0 | 0 |
| 12_21117 | 4H | 0 |
| 12_21131 | 1H | 59.99 |
| 12_21137 | 4H | 57.54 |
| 12_21157 | 0 | 0 |
| 12_21167 | 7H | 75.52 |
| 12_21172 | 1H | 121.56 |
| 12_21186 | 5H | 74.83 |
| 12_21208 | 7H | 116.28 |
| 12_21234 | 7H | 68.89 |
| 12_21290 | 5H | 167.4 |
| 12_21319 | 7H | 82.41 |
| 12_21328 | 7H | 151.76 |
| 12_21337 | 2H | 67.08 |
| 12_21372 | 5H | 39.48 |
| 12_21376 | 3H | 159.54 |
| 12_21386 | 3H | 154.44 |
| 12_21393 | 5H | 177.9 |
| 12_21415 | 2H | 3.84 |
| 12_21442 | 4H | 56.22 |
| 12_21462 | 5H | 143.29 |
| 12_21463 | 1H | 57.27 |
| 12_21471 | 5H | 125.09 |

| | | |
|---|---|---|
| 12_21475 | 3H | 67.86 |
| 12_21476 | 2H | 74.55 |
| 12_21477 | 6H | 125.99 |
| 12_21479 | 0 | 0 |
| 12_21482 | 6H | 59.25 |
| 12_21492 | 7H | 72.18 |
| 12_21497 | 5H | 83.17 |
| 12_21500 | 3H | 178.25 |
| 12_21522 | 0 | 0 |
| 12_21527 | 2H | 114.34 |
| 12_21531 | 3H | 158.05 |
| 12_30001 | 5H | 4.15 |
| 12_30002 | 0 | 0 |
| 12_30003 | 0 | 0 |
| 12_30004 | 7H | 81.78 |
| 12_30005 | 3H | 77.37 |
| 12_30007 | 5H | 60.21 |
| 12_30010 | 6H | 18.89 |
| 12_30011 | 5H | 52.86 |
| 12_30021 | 6H | 58.48 |
| 12_30025 | 6H | 127.01 |
| 12_30026 | 7H | 94.34 |
| 12_30032 | 6H | 56.06 |
| 12_30039 | 3H | 67.27 |
| 12_30040 | 7H | 26.14 |
| 12_30046 | 4H | 105.83 |
| 12_30048 | 1H | 143.2 |
| 12_30049 | 2H | 118.39 |
| 12_30053 | 7H | 81.78 |
| 12_30055 | 3H | 178.99 |
| 12_30056 | 5H | 107.19 |
| 12_30057 | 6H | 131.64 |
| 12_30060 | 4H | 62.81 |
| 12_30062 | 5H | 147.49 |
| 12_30063 | 7H | 26.14 |
| 12_30064 | 3H | 50.19 |
| 12_30065 | 7H | 36.26 |
| 12_30067 | 5H | 123.78 |
| 12_30068 | 2H | 67.08 |
| 12_30072 | 1H | 87.43 |
| 12_30080 | 5H | 60.21 |
| 12_30081 | 3H | 136.92 |
| 12_30083 | 7H | 29.7 |
| 12_30084 | 3H | 134.71 |
| 12_30090 | 3H | 108 |
| 12_30092 | 3H | 143.33 |
| 12_30095 | 2H | 117.17 |
| 12_30096 | 3H | 135.43 |
| 12_30097 | 2H | 139.29 |

| | | |
|---|---|---|
| 12_30098 | 5H | 94.4 |
| 12_30102 | 2H | 172.92 |
| 12_30108 | 2H | 72.99 |
| 12_30109 | 5H | 42.41 |
| 12_30110 | 1H | 45.2 |
| 12_30111 | 5H | 51.51 |
| 12_30113 | 3H | 17.66 |
| 12_30115 | 0 | 0 |
| 12_30116 | 0 | 0 |
| 12_30119 | 3H | 116.24 |
| 12_30120 | 6H | 56.06 |
| 12_30125 | 7H | 67.56 |
| 12_30126 | 3H | 67.86 |
| 12_30129 | 0 | 0 |
| 12_30130 | 3H | 67.27 |
| 12_30133 | 6H | 52.19 |
| 12_30135 | 3H | 179.81 |
| 12_30137 | 3H | 151.84 |
| 12_30138 | 4H | 98.7 |
| 12_30140 | 4H | 5.54 |
| 12_30141 | 7H | 29.05 |
| 12_30142 | 4H | 100.97 |
| 12_30143 | 7H | 38.57 |
| 12_30144 | 6H | 61.19 |
| 12_30148 | 6H | 89.58 |
| 12_30149 | 7H | 57.57 |
| 12_30150 | 4H | 16.96 |
| 12_30151 | 6H | 111.08 |
| 12_30153 | 0 | 0 |
| 12_30155 | 2H | 10.86 |
| 12_30158 | 4H | 109.65 |
| 12_30162 | 5H | 157.61 |
| 12_30163 | 5H | 0 |
| 12_30164 | 7H | 118.44 |
| 12_30165 | 5H | 155.45 |
| 12_30166 | 0 | 0 |
| 12_30167 | 5H | 20.48 |
| 12_30168 | 7H | 104.32 |
| 12_30169 | 5H | 121.67 |
| 12_30170 | 3H | 92.73 |
| 12_30178 | 2H | 87.25 |
| 12_30181 | 7H | 51.23 |
| 12_30183 | 5H | 147.49 |
| 12_30186 | 0 | 0 |
| 12_30197 | 0 | 0 |
| 12_30199 | 7H | 88.06 |
| 12_30200 | 2H | 113.53 |
| 12_30203 | 0 | 0 |
| 12_30204 | 1H | 89.77 |

| | | |
|---|---|---|
| 12_30205 | 2H | 82.44 |
| 12_30206 | 2H | 67.08 |
| 12_30213 | 7H | 83.42 |
| 12_30214 | 5H | 44.89 |
| 12_30216 | 2H | 107.92 |
| 12_30219 | 7H | 29.05 |
| 12_30221 | 0 | 0 |
| 12_30222 | 0 | 0 |
| 12_30223 | 3H | 129.83 |
| 12_30224 | 0 | 0 |
| 12_30226 | 4H | 89.36 |
| 12_30230 | 6H | 59.25 |
| 12_30231 | 1H | 141.46 |
| 12_30232 | 4H | 100.97 |
| 12_30236 | 6H | 83.28 |
| 12_30237 | 4H | 70.77 |
| 12_30239 | 4H | 143.35 |
| 12_30242 | 7H | 30.69 |
| 12_30243 | 1H | 48.99 |
| 12_30244 | 7H | 153.91 |
| 12_30250 | 3H | 106.67 |
| 12_30251 | 2H | 61.49 |
| 12_30259 | 2H | 56.64 |
| 12_30260 | 0 | 0 |
| 12_30268 | 1H | 36.64 |
| 12_30271 | 3H | 169.95 |
| 12_30274 | 3H | 133.36 |
| 12_30275 | 2H | 72.99 |
| 12_30276 | 3H | 125.11 |
| 12_30278 | 3H | 95.14 |
| 12_30283 | 0 | 0 |
| 12_30284 | 3H | 29.75 |
| 12_30285 | 0 | 0 |
| 12_30295 | 1H | 69.08 |
| 12_30298 | 1H | 73.13 |
| 12_30304 | 1H | 59.29 |
| 12_30305 | 6H | 59.91 |
| 12_30306 | 0 | 0 |
| 12_30310 | 2H | 140.69 |
| 12_30314 | 5H | 74.83 |
| 12_30316 | 6H | 52.19 |
| 12_30318 | 3H | 67.86 |
| 12_30319 | 6H | 0 |
| 12_30323 | 2H | 72.99 |
| 12_30325 | 3H | 101.37 |
| 12_30329 | 7H | 22.84 |
| 12_30331 | 4H | 53.94 |
| 12_30335 | 7H | 102.97 |
| 12_30336 | 1H | 35.11 |

| | | |
|---|---|---|
| 12_30337 | 5H | 42.41 |
| 12_30342 | 3H | 114.36 |
| 12_30343 | 1H | 51.91 |
| 12_30344 | 7H | 77.02 |
| 12_30346 | 6H | 65.83 |
| 12_30348 | 1H | 51.2 |
| 12_30350 | 1H | 45.2 |
| 12_30351 | 0 | 0 |
| 12_30352 | 2H | 156.77 |
| 12_30354 | 5H | 41.45 |
| 12_30358 | 6H | 38.66 |
| 12_30360 | 5H | 187.2 |
| 12_30362 | 7H | 114.96 |
| 12_30367 | 3H | 149.45 |
| 12_30368 | 7H | 118.52 |
| 12_30370 | 3H | 164.15 |
| 12_30371 | 7H | 47.1 |
| 12_30375 | 3H | 125.11 |
| 12_30377 | 5H | 121.01 |
| 12_30378 | 2H | 171.6 |
| 12_30379 | 2H | 48.8 |
| 12_30382 | 5H | 188.04 |
| 12_30385 | 4H | 118.31 |
| 12_30388 | 0 | 0 |
| 12_30389 | 7H | 81.78 |
| 12_30390 | 4H | 93.08 |
| 12_30394 | 4H | 26.2 |
| 12_30396 | 2H | 151.92 |
| 12_30399 | 3H | 86.57 |
| 12_30400 | 5H | 143.94 |
| 12_30402 | 2H | 10.86 |
| 12_30403 | 1H | 128.04 |
| 12_30404 | 1H | 42.42 |
| 12_30406 | 1H | 45.2 |
| 12_30408 | 5H | 42.41 |
| 12_30409 | 0 | 0 |
| 12_30410 | 5H | 31.2 |
| 12_30411 | 7H | 81.78 |
| 12_30414 | 6H | 137.41 |
| 12_30420 | 2H | 40.69 |
| 12_30423 | 3H | 120.73 |
| 12_30424 | 0 | 0 |
| 12_30425 | 4H | 139.97 |
| 12_30427 | 4H | 59.8 |
| 12_30430 | 6H | 56.06 |
| 12_30431 | 3H | 35.95 |
| 12_30432 | 2H | 44.04 |
| 12_30438 | 1H | 42.42 |
| 12_30441 | 6H | 58.48 |

| | | |
|---|---|---|
| 12_30444 | 0 | 0 |
| 12_30445 | 7H | 81.78 |
| 12_30449 | 7H | 81.78 |
| 12_30450 | 4H | 56.88 |
| 12_30454 | 5H | 41.45 |
| 12_30455 | 4H | 77 |
| 12_30456 | 5H | 107.19 |
| 12_30459 | 2H | 131.03 |
| 12_30467 | 3H | 53.86 |
| 12_30473 | 6H | 56.06 |
| 12_30474 | 3H | 59.83 |
| 12_30475 | 7H | 82.41 |
| 12_30476 | 4H | 139.97 |
| 12_30477 | 0 | 0 |
| 12_30488 | 4H | 56.22 |
| 12_30491 | 2H | 49.5 |
| 12_30492 | 7H | 81.78 |
| 12_30494 | 5H | 171.58 |
| 12_30496 | 7H | 79.08 |
| 12_30498 | 1H | 42.42 |
| 12_30502 | 0 | 0 |
| 12_30503 | 0 | 0 |
| 12_30504 | 5H | 173.5 |
| 12_30505 | 1H | 73.78 |
| 12_30506 | 7H | 83.42 |
| 12_30508 | 6H | 59.25 |
| 12_30509 | 1H | 107.09 |
| 12_30510 | 6H | 52.85 |
| 12_30514 | 2H | 68.07 |
| 12_30516 | 6H | 46.44 |
| 12_30517 | 0 | 0 |
| 12_30521 | 6H | 41.55 |
| 12_30522 | 1H | 47.21 |
| 12_30524 | 5H | 116.66 |
| 12_30525 | 4H | 56.22 |
| 12_30528 | 7H | 42.26 |
| 12_30530 | 7H | 20.29 |
| 12_30532 | 1H | 105.77 |
| 12_30533 | 5H | 86.74 |
| 12_30538 | 5H | 50.53 |
| 12_30543 | 5H | 1.91 |
| 12_30544 | 7H | 81.78 |
| 12_30545 | 7H | 46.44 |
| 12_30546 | 1H | 94.15 |
| 12_30550 | 7H | 81.78 |
| 12_30554 | 4H | 102.93 |
| 12_30556 | 5H | 139 |
| 12_30557 | 2H | 62.85 |
| 12_30561 | 2H | 68.07 |

| | | |
|---|---|---|
| 12_30562 | 1H | 42.42 |
| 12_30563 | 7H | 81.78 |
| 12_30564 | 4H | 53.28 |
| 12_30565 | 7H | 83.42 |
| 12_30566 | 5H | 162.68 |
| 12_30567 | 6H | 59.25 |
| 12_30571 | 3H | 32.92 |
| 12_30573 | 6H | 88.88 |
| 12_30574 | 7H | 82.41 |
| 12_30575 | 5H | 42.41 |
| 12_30576 | 7H | 52.52 |
| 12_30577 | 5H | 177.9 |
| 12_30580 | 5H | 143.94 |
| 12_30581 | 7H | 79.08 |
| 12_30582 | 2H | 68.07 |
| 12_30583 | 3H | 58.51 |
| 12_30584 | 4H | 105.17 |
| 12_30588 | 1H | 12.94 |
| 12_30589 | 7H | 81.78 |
| 12_30590 | 5H | 121.67 |
| 12_30591 | 5H | 6.36 |
| 12_30593 | 7H | 146.93 |
| 12_30594 | 0 | 0 |
| 12_30598 | 2H | 135.68 |
| 12_30600 | 7H | 81.78 |
| 12_30603 | 0 | 0 |
| 12_30604 | 2H | 55.46 |
| 12_30605 | 4H | 56.22 |
| 12_30609 | 3H | 52.17 |
| 12_30611 | 5H | 121.67 |
| 12_30616 | 3H | 78.25 |
| 12_30618 | 3H | 64.6 |
| 12_30619 | 5H | 108.28 |
| 12_30620 | 4H | 76.31 |
| 12_30621 | 7H | 81.78 |
| 12_30622 | 0 | 0 |
| 12_30626 | 6H | 103.32 |
| 12_30631 | 2H | 12.07 |
| 12_30634 | 2H | 65.53 |
| 12_30635 | 5H | 135.62 |
| 12_30637 | 6H | 72.17 |
| 12_30640 | 3H | 108.7 |
| 12_30642 | 5H | 157.61 |
| 12_30644 | 5H | 50.53 |
| 12_30645 | 7H | 84.3 |
| 12_30646 | 0 | 0 |
| 12_30651 | 6H | 7.87 |
| 12_30653 | 0 | 0 |
| 12_30654 | 5H | 38.78 |

| | | |
|---|---|---|
| 12_30655 | 0 | 0 |
| 12_30656 | 5H | 169.72 |
| 12_30657 | 2H | 42.01 |
| 12_30658 | 6H | 52.19 |
| 12_30663 | 3H | 100.29 |
| 12_30666 | 5H | 162.03 |
| 12_30668 | 5H | 130.98 |
| 12_30672 | 0 | 0 |
| 12_30674 | 2H | 85.52 |
| 12_30677 | 3H | 93.81 |
| 12_30680 | 3H | 61.94 |
| 12_30683 | 1H | 40.4 |
| 12_30685 | 0 | 0 |
| 12_30690 | 2H | 145.69 |
| 12_30693 | 4H | 79.47 |
| 12_30694 | 1H | 45.2 |
| 12_30696 | 2H | 89.68 |
| 12_30697 | 6H | 32.27 |
| 12_30698 | 6H | 98.68 |
| 12_30700 | 5H | 50.53 |
| 12_30703 | 2H | 50.99 |
| 12_30705 | 5H | 103.01 |
| 12_30707 | 5H | 36.72 |
| 12_30709 | 5H | 42.41 |
| 12_30710 | 1H | 49.65 |
| 12_30714 | 5H | 13.83 |
| 12_30715 | 1H | 3.21 |
| 12_30716 | 0 | 0 |
| 12_30717 | 5H | 42.41 |
| 12_30718 | 4H | 102.26 |
| 12_30721 | 3H | 55.22 |
| 12_30723 | 7H | 14.39 |
| 12_30724 | 2H | 72.99 |
| 12_30728 | 5H | 42.41 |
| 12_30729 | 5H | 41.45 |
| 12_30734 | 6H | 119.56 |
| 12_30736 | 3H | 173.43 |
| 12_30737 | 3H | 59.83 |
| 12_30742 | 1H | 74.81 |
| 12_30743 | 3H | 87.8 |
| 12_30745 | 5H | 55.44 |
| 12_30748 | 0 | 0 |
| 12_30750 | 1H | 45.2 |
| 12_30752 | 7H | 46.44 |
| 12_30754 | 3H | 84.22 |
| 12_30755 | 4H | 77.66 |
| 12_30760 | 7H | 81.78 |
| 12_30761 | 7H | 141.15 |
| 12_30762 | 1H | 42.42 |

| | | |
|---|---|---|
| 12_30765 | 6H | 59.25 |
| 12_30768 | 5H | 42.41 |
| 12_30772 | 2H | 66.2 |
| 12_30777 | 4H | 53.94 |
| 12_30779 | 0 | 0 |
| 12_30780 | 7H | 24.81 |
| 12_30781 | 2H | 9.12 |
| 12_30782 | 6H | 53.54 |
| 12_30783 | 6H | 44.96 |
| 12_30786 | 1H | 47.21 |
| 12_30788 | 3H | 78.25 |
| 12_30792 | 5H | 42.41 |
| 12_30793 | 0 | 0 |
| 12_30794 | 7H | 81.78 |
| 12_30795 | 5H | 148.58 |
| 12_30796 | 1H | 40.4 |
| 12_30797 | 7H | 116.94 |
| 12_30799 | 3H | 63.94 |
| 12_30802 | 6H | 58.48 |
| 12_30804 | 6H | 65.83 |
| 12_30806 | 7H | 99.94 |
| 12_30809 | 3H | 67.86 |
| 12_30818 | 0 | 0 |
| 12_30819 | 0 | 0 |
| 12_30820 | 1H | 4.71 |
| 12_30821 | 1H | 54.54 |
| 12_30822 | 0 | 0 |
| 12_30823 | 2H | 164.35 |
| 12_30824 | 4H | 146.48 |
| 12_30825 | 4H | 146.48 |
| 12_30826 | 7H | 162.03 |
| 12_30827 | 0 | 0 |
| 12_30828 | 2H | 67.08 |
| 12_30829 | 3H | 73.3 |
| 12_30830 | 5H | 149.27 |
| 12_30831 | 7H | 135.86 |
| 12_30832 | 7H | 79.08 |
| 12_30833 | 5H | 140.14 |
| 12_30834 | 5H | 88.05 |
| 12_30835 | 7H | 81.78 |
| 12_30836 | 7H | 5.43 |
| 12_30837 | 6H | 59.25 |
| 12_30838 | 5H | 35.39 |
| 12_30839 | 4H | 62.81 |
| 12_30840 | 0 | 0 |
| 12_30842 | 6H | 20.62 |
| 12_30843 | 6H | 24.91 |
| 12_30845 | 0 | 0 |
| 12_30847 | 5H | 98.2 |

| | | |
|---|---|---|
| 12_30850 | 5H | 98.2 |
| 12_30851 | 7H | 9.73 |
| 12_30852 | 5H | 98.2 |
| 12_30853 | 2H | 66.2 |
| 12_30854 | 5H | 98.2 |
| 12_30855 | 5H | 96.12 |
| 12_30857 | 6H | 61.19 |
| 12_30858 | 0 | 0 |
| 12_30859 | 0 | 0 |
| 12_30860 | 3H | 145.84 |
| 12_30863 | 4H | 35.24 |
| 12_30865 | 4H | 35.24 |
| 12_30866 | 4H | 56.88 |
| 12_30867 | 0 | 0 |
| 12_30871 | 2H | 25.33 |
| 12_30872 | 2H | 25.33 |
| 12_30873 | 4H | 142.6 |
| 12_30877 | 0 | 0 |
| 12_30878 | 4H | 56.22 |
| 12_30879 | 7H | 55.64 |
| 12_30880 | 7H | 54.99 |
| 12_30893 | 7H | 31.35 |
| 12_30894 | 7H | 31.35 |
| 12_30895 | 7H | 31.35 |
| 12_30896 | 2H | 99.39 |
| 12_30904 | 4H | 77.66 |
| 12_30905 | 4H | 77.66 |
| 12_30907 | 4H | 30.37 |
| 12_30908 | 0 | 0 |
| 12_30910 | 3H | 14 |
| 12_30913 | 3H | 48.88 |
| 12_30914 | 2H | 156.77 |
| 12_30917 | 0 | 0 |
| 12_30919 | 1H | 11.35 |
| 12_30921 | 3H | 163.49 |
| 12_30922 | 3H | 60.58 |
| 12_30923 | 3H | 60.58 |
| 12_30926 | 0 | 0 |
| 12_30927 | 3H | 127.26 |
| 12_30929 | 5H | 132.32 |
| 12_30930 | 5H | 132.32 |
| 12_30931 | 6H | 104.5 |
| 12_30933 | 1H | 6.98 |
| 12_30934 | 1H | 143.2 |
| 12_30935 | 0 | 0 |
| 12_30939 | 0 | 0 |
| 12_30940 | 6H | 88.88 |
| 12_30942 | 2H | 147.37 |
| 12_30944 | 0 | 0 |

| | | |
|---|---|---|
| 12_30945 | 0 | 0 |
| 12_30948 | 1H | 15.91 |
| 12_30949 | 0 | 0 |
| 12_30950 | 1H | 11.35 |
| 12_30953 | 3H | 40.99 |
| 12_30956 | 6H | 142.2 |
| 12_30957 | 0 | 0 |
| 12_30958 | 5H | 189.22 |
| 12_30959 | 7H | 0 |
| 12_30962 | 0 | 0 |
| 12_30963 | 3H | 137.74 |
| 12_30965 | 0 | 0 |
| 12_30967 | 0 | 0 |
| 12_30969 | 1H | 0.31 |
| 12_30972 | 0 | 0 |
| 12_30973 | 3H | 141.26 |
| 12_30974 | 7H | 157.44 |
| 12_30975 | 5H | 4.15 |
| 12_30976 | 5H | 1.91 |
| 12_30977 | 5H | 4.15 |
| 12_30979 | 5H | 4.15 |
| 12_30980 | 5H | 1.91 |
| 12_30981 | 0 | 0 |
| 12_30988 | 4H | 111.81 |
| 12_30993 | 4H | 49.43 |
| 12_30995 | 4H | 62.81 |
| 12_30996 | 7H | 94.34 |
| 12_30998 | 7H | 85.28 |
| 12_30999 | 0 | 0 |
| 12_31000 | 7H | 81.78 |
| 12_31004 | 6H | 58.48 |
| 12_31005 | 6H | 58.48 |
| 12_31006 | 6H | 60.65 |
| 12_31008 | 3H | 65.25 |
| 12_31009 | 3H | 50.85 |
| 12_31010 | 3H | 67.86 |
| 12_31011 | 3H | 71.26 |
| 12_31012 | 3H | 65.25 |
| 12_31014 | 3H | 67.86 |
| 12_31015 | 3H | 60.58 |
| 12_31016 | 3H | 71.26 |
| 12_31017 | 3H | 67.86 |
| 12_31018 | 3H | 100.29 |
| 12_31020 | 2H | 82.44 |
| 12_31021 | 2H | 82.44 |
| 12_31023 | 5H | 4.15 |
| 12_31032 | 5H | 52.86 |
| 12_31033 | 5H | 52.86 |
| 12_31035 | 5H | 52.86 |

| | | |
|---|---|---|
| 12_31041 | 0 | 0 |
| 12_31042 | 6H | 112.39 |
| 12_31043 | 6H | 112.39 |
| 12_31044 | 6H | 112.39 |
| 12_31048 | 6H | 112.39 |
| 12_31049 | 6H | 112.39 |
| 12_31050 | 5H | 143.29 |
| 12_31053 | 0 | 0 |
| 12_31054 | 0 | 0 |
| 12_31055 | 0 | 0 |
| 12_31059 | 0 | 0 |
| 12_31062 | 5H | 51.51 |
| 12_31064 | 5H | 51.51 |
| 12_31065 | 0 | 0 |
| 12_31066 | 0 | 0 |
| 12_31071 | 0 | 0 |
| 12_31081 | 1H | 143.2 |
| 12_31086 | 0 | 0 |
| 12_31088 | 6H | 83.81 |
| 12_31092 | 6H | 52.85 |
| 12_31094 | 5H | 11.45 |
| 12_31095 | 2H | 133.04 |
| 12_31096 | 0 | 0 |
| 12_31099 | 0 | 0 |
| 12_31100 | 2H | 142.67 |
| 12_31101 | 6H | 80.86 |
| 12_31109 | 0 | 0 |
| 12_31111 | 6H | 83.28 |
| 12_31113 | 0 | 0 |
| 12_31115 | 6H | 112.39 |
| 12_31117 | 5H | 46.38 |
| 12_31120 | 7H | 75.52 |
| 12_31122 | 3H | 59.83 |
| 12_31123 | 5H | 189.89 |
| 12_31126 | 6H | 131.64 |
| 12_31127 | 0 | 0 |
| 12_31134 | 1H | 45.2 |
| 12_31138 | 4H | 118.95 |
| 12_31139 | 4H | 114.61 |
| 12_31144 | 1H | 4.71 |
| 12_31148 | 4H | 88.3 |
| 12_31151 | 0 | 0 |
| 12_31153 | 3H | 80.49 |
| 12_31159 | 3H | 40.99 |
| 12_31160 | 1H | 74.81 |
| 12_31161 | 0 | 0 |
| 12_31163 | 1H | 95.14 |
| 12_31164 | 4H | 33.35 |
| 12_31165 | 5H | 147.49 |

| | | |
|---|---|---|
| 12_31166 | 7H | 145.6 |
| 12_31167 | 0 | 0 |
| 12_31173 | 7H | 5.43 |
| 12_31174 | 6H | 60.65 |
| 12_31175 | 2H | 68.07 |
| 12_31179 | 1H | 61.39 |
| 12_31180 | 2H | 168.26 |
| 12_31181 | 0 | 0 |
| 12_31182 | 5H | 141.88 |
| 12_31183 | 5H | 50.53 |
| 12_31186 | 4H | 72.36 |
| 12_31187 | 6H | 59.25 |
| 12_31189 | 2H | 67.08 |
| 12_31194 | 0 | 0 |
| 12_31200 | 0 | 0 |
| 12_31202 | 0 | 0 |
| 12_31203 | 0 | 0 |
| 12_31205 | 2H | 99.39 |
| 12_31206 | 5H | 144.6 |
| 12_31207 | 0 | 0 |
| 12_31208 | 1H | 42.42 |
| 12_31209 | 2H | 156.77 |
| 12_31210 | 5H | 187.2 |
| 12_31215 | 7H | 81.78 |
| 12_31218 | 2H | 67.08 |
| 12_31219 | 0 | 0 |
| 12_31220 | 3H | 129.83 |
| 12_31221 | 5H | 148.58 |
| 12_31222 | 0 | 0 |
| 12_31224 | 2H | 4.59 |
| 12_31225 | 6H | 105.05 |
| 12_31229 | 0 | 0 |
| 12_31230 | 0 | 0 |
| 12_31231 | 4H | 86.01 |
| 12_31234 | 5H | 141.88 |
| 12_31235 | 6H | 103.32 |
| 12_31236 | 5H | 103.01 |
| 12_31237 | 5H | 132.32 |
| 12_31238 | 3H | 135.43 |
| 12_31239 | 0 | 0 |
| 12_31240 | 0 | 0 |
| 12_31242 | 3H | 82.62 |
| 12_31246 | 4H | 92.41 |
| 12_31249 | 6H | 59.25 |
| 12_31250 | 6H | 80.06 |
| 12_31251 | 3H | 147.57 |
| 12_31254 | 0 | 0 |
| 12_31256 | 2H | 73.89 |
| 12_31257 | 5H | 39.48 |

| | | |
|---|---|---|
| 12_31259 | 5H | 42.41 |
| 12_31261 | 7H | 108.78 |
| 12_31262 | 3H | 93.81 |
| 12_31264 | 2H | 135.68 |
| 12_31267 | 0 | 0 |
| 12_31268 | 2H | 147.37 |
| 12_31270 | 0 | 0 |
| 12_31271 | 5H | 86.08 |
| 12_31274 | 6H | 52.85 |
| 12_31276 | 1H | 25.5 |
| 12_31277 | 6H | 129.07 |
| 12_31279 | 0 | 0 |
| 12_31280 | 5H | 52.86 |
| 12_31284 | 2H | 15.98 |
| 12_31286 | 0 | 0 |
| 12_31288 | 2H | 63.55 |
| 12_31289 | 6H | 77.53 |
| 12_31292 | 5H | 184.99 |
| 12_31293 | 2H | 93.26 |
| 12_31294 | 7H | 105.73 |
| 12_31297 | 4H | 62.81 |
| 12_31298 | 3H | 40.99 |
| 12_31299 | 3H | 98.05 |
| 12_31305 | 7H | 34.15 |
| 12_31308 | 6H | 33.72 |
| 12_31312 | 5H | 38.78 |
| 12_31313 | 4H | 32.92 |
| 12_31315 | 0 | 0 |
| 12_31319 | 1H | 100.4 |
| 12_31323 | 3H | 83.58 |
| 12_31324 | 4H | 0.75 |
| 12_31325 | 7H | 142.46 |
| 12_31326 | 0 | 0 |
| 12_31327 | 0 | 0 |
| 12_31329 | 3H | 125.86 |
| 12_31333 | 0 | 0 |
| 12_31340 | 5H | 51.51 |
| 12_31346 | 3H | 88.5 |
| 12_31350 | 7H | 3.49 |
| 12_31351 | 7H | 24.15 |
| 12_31352 | 5H | 181.21 |
| 12_31353 | 6H | 111.08 |
| 12_31356 | 3H | 85.4 |
| 12_31357 | 0 | 0 |
| 12_31360 | 4H | 56.22 |
| 12_31361 | 5H | 80.18 |
| 12_31362 | 4H | 83.75 |
| 12_31363 | 7H | 116.94 |
| 12_31366 | 5H | 136.94 |

| | | |
|---|---|---|
| 12_31367 | 3H | 106 |
| 12_31368 | 3H | 67.86 |
| 12_31374 | 7H | 126.83 |
| 12_31375 | 5H | 157.61 |
| 12_31377 | 1H | 128.04 |
| 12_31380 | 2H | 85.52 |
| 12_31381 | 1H | 44.59 |
| 12_31382 | 4H | 53.94 |
| 12_31383 | 2H | 84.96 |
| 12_31385 | 4H | 77.66 |
| 12_31387 | 1H | 129.71 |
| 12_31390 | 5H | 41.45 |
| 12_31392 | 6H | 127.76 |
| 12_31393 | 3H | 71.26 |
| 12_31394 | 2H | 85.52 |
| 12_31395 | 7H | 99.94 |
| 12_31396 | 0 | 0 |
| 12_31398 | 2H | 89.68 |
| 12_31401 | 1H | 61.39 |
| 12_31406 | 2H | 136.33 |
| 12_31408 | 0 | 0 |
| 12_31409 | 3H | 9.56 |
| 12_31410 | 0 | 0 |
| 12_31411 | 0 | 0 |
| 12_31414 | 0 | 0 |
| 12_31417 | 5H | 96.12 |
| 12_31418 | 7H | 81.78 |
| 12_31422 | 4H | 144.09 |
| 12_31423 | 5H | 41.45 |
| 12_31424 | 2H | 101.72 |
| 12_31427 | 5H | 79.52 |
| 12_31428 | 3H | 0 |
| 12_31431 | 0 | 0 |
| 12_31432 | 6H | 107.49 |
| 12_31433 | 6H | 52.85 |
| 12_31440 | 7H | 105.07 |
| 12_31441 | 7H | 64.98 |
| 12_31443 | 6H | 59.25 |
| 12_31445 | 2H | 90.48 |
| 12_31446 | 2H | 0.66 |
| 12_31448 | 3H | 6.31 |
| 12_31450 | 7H | 7.87 |
| 12_31452 | 7H | 69.55 |
| 12_31458 | 4H | 12.34 |
| 12_31461 | 2H | 155.68 |
| 12_31462 | 4H | 62.81 |
| 12_31463 | 7H | 61.67 |
| 12_31464 | 1H | 64.44 |
| 12_31467 | 1H | 40.4 |

| | | |
|---|---|---|
| 12_31469 | 6H | 136.66 |
| 12_31473 | 0 | 0 |
| 12_31477 | 5H | 56.77 |
| 12_31479 | 0 | 0 |
| 12_31484 | 0 | 0 |
| 12_31485 | 6H | 32.98 |
| 12_31486 | 4H | 6.86 |
| 12_31490 | 0 | 0 |
| 12_31493 | 4H | 77.66 |
| 12_31495 | 6H | 122.14 |
| 12_31498 | 6H | 136.66 |
| 12_31499 | 3H | 131.24 |
| 12_31500 | 3H | 160.19 |
| 12_31502 | 3H | 67.27 |
| 12_31506 | 2H | 166.6 |
| 12_31509 | 6H | 59.25 |
| 12_31511 | 0 | 0 |
| 12_31512 | 5H | 41.45 |
| 12_31513 | 0 | 0 |
| 12_31515 | 4H | 76.31 |
| 12_31517 | 0 | 0 |
| 12_31520 | 5H | 42.41 |
| 12_31521 | 0 | 0 |
| 12_31523 | 0 | 0 |
| 12_31525 | 3H | 134.05 |
| 12_31527 | 2H | 165.28 |
| 12_31529 | 3H | 85.4 |
| 12_31535 | 7H | 124.5 |
| 12_31536 | 4H | 79.47 |
