# Supplementary material for: Two Genomic Regions Contribute Disproportionately to Geographic Differentiation in Wild Barley
Source: G3 (Bethesda). 2014 Apr 22;4(7):1193–203. doi: 10.1534/g3.114.010561 (PMC4455769; doi:10.1534/g3.114.010561)
Supplement: Supporting Information [file supp_g3.114.010561_TableS6.pdf]

Table S6 SNPs with F_{ST} based on the Eastern and Western populations above 95th percentile genome-wide, including genetic position, GenBank ID, gene short name, in non-coding or coding region (1st, 2nd or 3rd positions), and silent or replacement information

SNP Name	Chr	cM	F_{ST}	GenBank ID	Gene Short Name	Position	Silent
11_10017	2H	81.31	0.32	AK353943	-	3	yes
11_10056	7H	33.49	0.41	AK364330	-	non-coding	yes
11_10116	5H	41.45	0.36	AK356434	-	3	yes
11_10243	2H	67.08	0.41	XM_003579657	LOC100838855	non-coding	yes
11_10253	3H	102.66	0.36	-	-	-	-
11_10424	4H	56.22	0.42	AK355297	-	3	yes
11_10498	2H	53.09	0.35	AK354555	-	1	yes
11_10536	5H	155.23	0.49	AK354787	-	3	yes
11_10614	4H	111.81	0.33	AK373775	-	3	yes
11_10644	1H	128.63	0.32	AK356375	-	non-coding	yes
11_10685	2H	72.99	0.47	AK362284	-	3	yes
11_10773	7H	81.78	0.36	AK373162	-	non-coding	yes
11_10813	3H	83.58	0.35	-	-	-	-
11_10956	7H	27.69	0.38	AK365588	-	3	yes
11_11024	5H	121.67	0.48	XM_003561753	LOC100839369	3	yes
11_11094	2H	119.72	0.38	-	-	-	-
11_11111	6H	139.09	0.39	AK366470	-	non-coding	yes
11_11243	7H	121.36	0.33	-	-	-	-
11_11432	5H	38.78	0.56	-	-	-	-
11_20029	6H	133.25	0.51	AK354118	-	1	no
11_20086	2H	110.93	0.32	AK358640	-	non-coding	yes
11_20133	1H	132.16	0.47	AK361091	-	non-coding	yes
11_20251	2H	68.56	0.36	AK370757	-	non-coding	yes
11_20283	5H	51.51	0.32	XM_003576993	LOC100831472	3	yes
11_20347	5H	121.67	0.48	AK359986	-	3	yes
11_20390	2H	72.99	0.46	FN179383	SBE2a	3	yes
11_20476	2H	67.08	0.33	BT087333	-	non-coding	yes
11_20482	4H	69.24	0.31	AK355084	-	3	yes
11_20498	2H	116.5	0.4	AK359654	-	3	yes
11_20577	6H	80.06	0.31	AK355031	-	non-coding	yes
11_20620	6H	78.52	0.31	AK366751	-	non-coding	yes
11_20669	2H	68.56	0.34	AK356298	-	non-coding	yes
11_20798	1H	48.99	0.32	AK354088	-	non-coding	yes
11_20904	6H	72.17	0.36	-	-	-	-
11_21302	7H	81.78	0.33	XM_003560861	LOC100844834	non-coding	yes

11_21399	2H	72.99	0.69	AK357878	-	1	no
11_21406	2H	142.67	0.34	AK370573	-	2	no
11_21447	5H	41.45	0.53	AK364513	-	2	no
11_21452	5H	155.23	0.53	AK372156	-	1	no
11_21502	3H	76.43	0.55	AK356987	-	3	yes
11_21504	4H	80.73	0.45	-	-	-	-
12_10053	4H	76.31	0.46	AK370758	-	3	yes
12_10071	6H	130.38	0.47	AK355485	-	non-coding	yes
12_10154	2H	69.05	0.71	AK355324	-	non-coding	yes
12_10170	4H	88.7	0.44	AK373474	-	non-coding	yes
12_10171	4H	43.72	0.34	AK368127	-	1	no
12_10199	6H	49.67	0.64	AK376992	-	non-coding	yes
12_10203	5H	59.72	0.52	AK356265	-	3	yes
12_10219	0	0	0.33	AY039003	Xantha-f	3	yes
12_10264	5H	47.04	0.52	WHTE1A	E1	non-coding	yes
12_10284	0	0	0.47	XM_003564030	LOC100843138	3	yes
12_10347	4H	43.72	0.5	AK362515	-	non-coding	yes
12_10472	2H	147.37	0.31	AK356296	-	non-coding	yes
12_10497	6H	56.06	0.48	XM_003570288	LOC100839823	1	no
12_10543	7H	121.36	0.4	DQ529207	Xantha-h	non-coding	yes
12_10591	6H	59.25	0.44	AK361815	-	1	no
12_10633	5H	69.29	0.32	AK371801	-	non-coding	yes
12_10634	5H	68.21	0.32	AK360310	-	3	yes
12_10689	0	0	0.38	AK369452	-	non-coding	yes
12_10810	4H	37.88	0.69	AK366265	-	non-coding	yes
12_11030	2H	6.09	0.31	AK364311	-	non-coding	yes
12_11107	1H	47.21	0.35	AK364999	-	non-coding	yes
12_11139	4H	111.81	0.38	Y14573	Mlo	3	yes
12_11151	5H	51.51	0.88	AK354730	-	3	yes
12_11184	7H	118.44	0.42	AK358083	-	non-coding	yes
12_11269	0	0	0.41	AK361959	-	1	no
12_11271	1H	136.7	0.4	-	-	-	-
12_11288	2H	67.08	0.52	AK366035	-	3	no
12_11310	3H	13.13	0.36	AK372013	-	3	yes
12_11316	2H	73.89	0.49	AK354712	-	non-coding	yes
12_11324	2H	72.99	0.91	AK356277	-	non-coding	yes
12_11377	7H	84.3	0.34	AK361273	-	3	yes
12_11408	0	0	0.41	-	-	-	-
12_20196	2H	67.08	0.55	AK362518	-	non-coding	yes

12_20235	2H	61.49	0.4	AK376421	-	non-coding	yes
12_20278	5H	59.72	0.52	AK356265	-	1	no
12_20326	2H	42.01	0.36	AK365480	-	non-coding	yes
12_20593	2H	29.05	0.44	AK367163	-	non-coding	yes
12_20760	4H	138.7	0.39	-	-	-	-
12_20981	5H	51.51	0.85	AK370568	-	3	yes
12_20989	2H	130.38	0.51	XM_003580352	LOC100845048	2	no
12_21003	0	0	0.76	-	-	-	-
12_21117	4H	0	0.32	AK359776	-	non-coding	yes
12_21234	7H	68.89	0.54	AK356095	-	2	no
12_21319	7H	82.41	0.37	XM_003573274	LOC100845308	non-coding	yes
12_30004	7H	81.78	0.34	-	-	-	-
12_30049	2H	118.39	0.41	-	-	-	-
12_30056	5H	107.19	0.35	AK356430	-	non-coding	yes
12_30068	2H	67.08	0.52	AK364966	-	3	yes
12_30164	7H	118.44	0.41	AK360970	-	non-coding	yes
12_30250	3H	106.67	0.65	AK356601	-	non-coding	yes
12_30441	6H	58.48	0.36	XM_003570517	LOC100832867	3	yes
12_30492	7H	81.78	0.41	EU961304	-	1	no
12_30504	5H	173.5	0.32	-	-	-	-
12_30563	7H	81.78	0.33	AJ582181	core3ft	2	no
12_30581	7H	79.08	0.45	AK356791	-	3	yes
12_30600	7H	81.78	0.54	AK362488	-	non-coding	yes
12_30616	3H	78.25	0.58	AK365474	-	non-coding	yes
12_30694	1H	45.2	0.6	-	-	-	-
12_30737	3H	59.83	0.39	AK361759	-	3	yes
12_30765	6H	59.25	0.44	AK356029	-	3	yes
12_30823	2H	164.35	0.32	AK375658	-	3	no
12_30850	5H	98.2	0.63	DQ480160	CBF4B	non-coding	yes
12_30956	6H	142.2	0.35	-	-	-	-
12_30988	4H	111.81	0.52	Y14573	Mlo	non-coding	yes
12_31017	3H	67.86	0.31	AK356796	-	non-coding	yes
12_31021	2H	82.44	0.51	AF112963	Cht2	non-coding	yes
12_31032	5H	52.86	0.33	AF326715	adh3	non-coding	yes
12_31035	5H	52.86	0.33	DQ195967	adh3	non-coding	yes
12_31043	6H	112.39	0.31	AY349220	Dhn5	3	yes
12_31062	5H	51.51	0.48	AK365941	-	3	yes
12_31064	5H	51.51	0.68	AK365941	-	3	yes
12_31100	2H	142.67	0.34	AK370573	-	2	no

12_31202	0	0	0.33	AK358464	-	non-coding	yes
12_31203	0	0	0.34	AK368679	-	non-coding	yes
12_31242	3H	82.62	0.36	AK369382	-	non-coding	yes
12_31246	4H	92.41	0.48	AK362249	-	non-coding	yes
12_31274	6H	52.85	0.38	AK357603	-	non-coding	yes
12_31385	4H	77.66	0.32	AK357832	-	non-coding	yes
