# Supplementary material for: Two Genomic Regions Contribute Disproportionately to Geographic Differentiation in Wild Barley
Source: G3 (Bethesda). 2014 Apr 22;4(7):1193–203. doi: 10.1534/g3.114.010561 (PMC4455769; doi:10.1534/g3.114.010561)
Supplement: Supporting Information [file supp_g3.114.010561_TableS7.pdf]

Table S7 SNPs with Bayes Factor from environmental association analysis (Bayenv) above 95th percentile genome-wide, including genetic position, GenBank ID, gene short name, in non-coding or coding region (1st, 2nd or 3rd positions), and silent or replacement information. (A) PC1; (B) PC2

(A)

SNP Name	Chr	cM	Bayes Factor	GenBank ID	Gene Short Name	Position	Silent
11_10011	3H	67.86	9.22	AK371824	-	non-coding	yes
11_10090	4H	86.01	3.91	AK376851	-	non-coding	yes
11_10172	3H	82.62	5.04	AK366339	-	non-coding	yes
11_10184	3H	110.75	3.72	AK359211	-	3	yes
11_10208	4H	2.6	3.76	AK359180	-	3	yes
11_10232	7H	29.05	3.89	-	-	-	-
11_10247	4H	87.01	4.32	AJ585385	bcb	3	no
11_10253	3H	102.66	6.72	-	-	-	-
11_10446	2H	140.69	4.00	XM_003560174	LOC100837523	1	no
11_10641	5H	59.72	4.10	AK355250	-	3	yes
11_10653	3H	69.4	4.18	AK356876	-	2	no
11_10744	1H	23.62	4.80	AK353610	-	non-coding	yes
11_10813	3H	83.58	8.40	-	-	-	-
11_10818	2H	90.48	5.18	AK360545	-	non-coding	yes
11_10925	3H	66.62	3.83	AK363708	-	non-coding	yes
11_10989	2H	125.97	6.42	BLYCLDAB	cold-regulated	non-coding	yes
11_11111	6H	139.09	4.38	AK366470	-	non-coding	yes
11_11211	2H	67.08	4.06	-	-	-	-
11_11361	0	0	9.94	JF796668	CBF4	2	no
11_11497	5H	151.35	6.31	AK358288	-	3	yes
11_11521	7H	127.74	18.19	AK376764	-	3	yes
11_20012	4H	43.72	3.87	AK374823	-	non-coding	yes
11_20078	5H	155.66	5.77	AK376547	-	3	yes
11_20113	7H	49.61	5.40	AK355306	-	non-coding	yes
11_20133	1H	132.16	3.67	AK361091	-	non-coding	yes
11_20251	2H	68.56	5.27	AK370757	-	non-coding	yes
11_20306	5H	50.53	5.22	AK365203	-	non-coding	yes
11_20495	7H	18.73	3.96	AK359539	-	non-coding	yes
11_20537	6H	142.2	4.60	FJ853600	GSL2	non-coding	yes
11_20583	3H	66.62	4.92	AK365999	-	non-coding	yes
11_20620	6H	78.52	7.50	AK366751	-	non-coding	yes
11_20626	3H	109.43	3.65	AK366712	-	3	yes
11_20722	7H	13.7	5.24	AK377052	-	3	yes
11_20797	3H	5.36	3.96	AK375179	-	non-coding	yes

11_21030	6H	44.96	3.79	AK375921	-	2	no
11_21083	3H	111.49	4.11	AK372580	-	non-coding	yes
11_21174	1H	8.96	4.49	-	-	-	-
11_21191	4H	70.77	3.46	AK355907	-	non-coding	yes
11_21192	1H	89.77	5.99	AK357712	-	non-coding	yes
11_21193	1H	42.42	7.48	AK363338	-	non-coding	yes
11_21201	7H	98.95	5.56	-	-	-	-
11_21216	6H	59.25	5.25	AK376749	-	3	yes
11_21274	2H	154.39	3.85	AK354727	-	non-coding	yes
11_21296	4H	71.71	4.88	AK353992	-	non-coding	yes
11_21325	5H	123.12	3.84	AK362100	-	non-coding	yes
11_21399	2H	72.99	8.21	AK357878	-	1	no
11_21406	2H	142.67	3.68	AK370573	-	2	no
11_21459	2H	143.18	4.78	AM039897	ahh1	2	no
11_21502	3H	76.43	3.57	AK356987	-	3	yes
12_10122	3H	143.33	4.09	AK369540	-	non-coding	yes
12_10154	2H	69.05	14.43	AK355324	-	non-coding	yes
12_10199	6H	49.67	4.42	AK376992	-	non-coding	yes
12_10268	7H	81.78	5.32	XM_003574799	LOC100825330	3	yes
12_10535	1H	91.73	9.41	AK358527	-	3	yes
12_10678	3H	71.26	4.44	AK356724	-	non-coding	yes
12_10810	4H	37.88	3.49	AK366265	-	non-coding	yes
12_10824	4H	102.93	4.39	XM_003577507	LOC100843401	non-coding	yes
12_10938	1H	44.59	4.96	AK359548	-	1	no
12_11030	2H	6.09	4.18	AK364311	-	non-coding	yes
12_11051	7H	105.73	7.91	-	-	-	-
12_11151	5H	51.51	3.94	AK354730	-	3	yes
12_11154	3H	147.57	3.85	-	-	-	-
12_11271	1H	136.7	11.25	-	-	-	-
12_11288	2H	67.08	5.01	AK366035	-	3	no
12_11324	2H	72.99	41.73	AK356277	-	non-coding	yes
12_11386	0	0	5.77	-	-	-	-
12_11408	0	0	4.50	-	-	-	-
12_11455	6H	44.96	4.34	AK362081	-	non-coding	yes
12_11494	6H	111.74	3.53	AK368552	-	non-coding	yes
12_20235	2H	61.49	5.61	AK376421	-	non-coding	yes
12_20760	4H	138.7	8.63	-	-	-	-
12_20867	5H	167.4	3.85	AK359088	-	non-coding	yes
12_20981	5H	51.51	4.68	AK370568	-	3	yes

12_21117	4H	0	5.34	AK359776	-	non-coding	yes
12_21234	7H	68.89	3.83	AK356095	-	2	no
12_21319	7H	82.41	4.40	XM_003573274	LOC100845308	non-coding	yes
12_21337	2H	67.08	3.88	AK358015	-	3	yes
12_30004	7H	81.78	4.04	-	-	-	-
12_30060	4H	62.81	9.76	AK365085	-	non-coding	yes
12_30064	3H	50.19	3.70	AK367843	-	non-coding	yes
12_30068	2H	67.08	5.01	AK364966	-	3	yes
12_30080	5H	60.21	3.66	-	-	-	-
12_30135	3H	179.81	3.94	AK356358	-	non-coding	yes
12_30170	3H	92.73	3.50	AK353673	-	3	yes
12_30206	2H	67.08	3.88	-	-	-	-
12_30367	3H	149.45	15.26	AK353825	-	non-coding	yes
12_30404	1H	42.42	4.06	AK355367	-	3	yes
12_30475	7H	82.41	5.64	AK372209	-	non-coding	yes
12_30491	2H	49.5	3.90	-	-	-	-
12_30492	7H	81.78	5.94	EU961304	-	1	no
12_30504	5H	173.5	6.24	-	-	-	-
12_30554	4H	102.93	5.72	AK354013	-	2	no
12_30574	7H	82.41	4.96	AK370386	-	3	yes
12_30581	7H	79.08	18.82	AK356791	-	3	yes
12_30674	2H	85.52	4.34	AK369978	-	non-coding	yes
12_30715	1H	3.21	4.57	AK362579	-	non-coding	yes
12_30724	2H	72.99	3.51	AK368064	-	3	yes
12_30743	3H	87.8	4.49	AK364775	-	non-coding	yes
12_30745	5H	55.44	3.61	AK366468	-	3	yes
12_30779	0	0	5.07	AK376832	-	non-coding	yes
12_30781	2H	9.12	4.05	AK361785	-	non-coding	yes
12_30782	6H	53.54	6.44	-	-	-	-
12_30806	7H	99.94	4.96	-	-	-	-
12_31032	5H	52.86	3.59	AF326715	adh3	non-coding	yes
12_31066	0	0	4.85	AK372734	-	3	yes
12_31081	1H	143.2	21.95	AK356376	-	3	yes
12_31100	2H	142.67	3.68	AK370573	-	2	no
12_31218	2H	67.08	5.54	AK368932	-	2	no
12_31234	5H	141.88	9.78	AK358248	-	non-coding	yes
12_31357	0	0	6.10	-	-	-	-
12_31363	7H	116.94	3.45	AK375416	-	non-coding	yes
12_31381	1H	44.59	3.44	-	-	-	-

12_31383	2H	84.96	3.84	AK363632	-	non-coding	yes
12_31385	4H	77.66	4.68	AK357832	-	non-coding	yes
12_31392	6H	127.76	5.64	AK354269	-	non-coding	yes
12_31424	2H	101.72	3.69	AK375713	-	non-coding	yes
12_31486	4H	6.86	30.04	-	-	-	-

(B)

SNP Name	Chr	cM	Bayes Factor	GenBank ID	Gene Short Name	Position	Silent
11_10006	1H	76.92	4.55	AM502852	tub4	3	yes
11_10052	4H	76.31	3.08	AK370758	-	non-coding	yes
11_10129	6H	44.96	4.94	AK370146	-	3	yes
11_10172	3H	82.62	3.72	AK366339	-	non-coding	yes
11_10194	2H	67.08	2.86	AK359415	-	3	yes
11_10217	5H	149.94	3.16	AK354403	-	non-coding	yes
11_10240	5H	42.41	2.81	AK359441	-	non-coding	yes
11_10329	2H	168.26	3.71	AK362809	-	non-coding	yes
11_10424	4H	56.22	2.83	AK355297	-	3	yes
11_10522	1H	101.34	6.60	AK360842	-	1	yes
11_10580	5H	29.9	18.54	AK372891	-	non-coding	yes
11_10641	5H	59.72	5.49	AK355250	-	3	yes
11_10653	3H	69.4	2.96	AK356876	-	2	no
11_10767	3H	179.16	4.15	-	-	-	-
11_10780	2H	137.03	6.67	AK359167	-	3	yes
11_10919	2H	42.01	2.92	AK368475	-	non-coding	yes
11_11019	4H	146.48	2.85	FN179393	BAM1	2	no
11_11111	6H	139.09	4.09	AK366470	-	non-coding	yes
11_11147	6H	95.7	2.92	GU258512	-	non-coding	yes
11_11219	7H	82.2	5.81	AK368527	-	2	no
11_11406	6H	6.54	5.20	AF166121	Big1	3	yes
11_11521	7H	127.74	4.21	AK376764	-	3	yes
11_20114	4H	44.99	3.66	AK360127	-	3	yes
11_20129	5H	42.41	2.90	AK362176	-	non-coding	yes
11_20179	5H	42.41	2.85	AK357883	-	non-coding	yes
11_20260	1H	42.42	7.95	AK355367	-	3	yes
11_20306	5H	50.53	4.87	AK365203	-	non-coding	yes
11_20332	5H	49.16	3.04	WHTE1A	E1	non-coding	yes
11_20347	5H	121.67	4.55	AK359986	-	3	yes
11_20355	6H	120.69	3.68	AK353922	-	non-coding	yes

11_20383	1H	134.96	5.76	XM_003567791	LOC100830416	3	yes
11_20390	2H	72.99	10.41	FN179383	SBE2a	3	yes
11_20438	2H	69.05	4.78	AK364463	-	non-coding	yes
11_20485	7H	91.67	4.67	-	-	-	-
11_20498	2H	116.5	2.96	AK359654	-	3	yes
11_20527	3H	142.17	5.48	XM_003572099	LOC100831350	3	yes
11_20620	6H	78.52	12.93	AK366751	-	non-coding	yes
11_20709	6H	74.65	2.86	AK363507	-	3	yes
11_20736	5H	72.68	3.59	AK372237	-	3	yes
11_20797	3H	5.36	4.23	AK375179	-	non-coding	yes
11_20889	6H	86.54	3.18	XM_002454244	-	non-coding	yes
11_20924	4H	75.44	5.18	AK376504	-	1	no
11_20958	5H	42.41	2.90	AK372310	-	3	yes
11_20996	6H	104.5	5.87	AK360096	-	3	yes
11_21000	1H	45.85	6.15	AK370060	-	3	yes
11_21040	5H	42.41	2.81	AK370183	-	non-coding	yes
11_21181	2H	155.68	6.75	AK354575	-	3	yes
11_21193	1H	42.42	7.31	AK363338	-	non-coding	yes
11_21201	7H	98.95	10.97	-	-	-	-
11_21399	2H	72.99	8.83	AK357878	-	1	no
11_21494	7H	81.78	3.46	-	-	-	-
12_10053	4H	76.31	6.89	AK370758	-	3	yes
12_10063	4H	44.99	3.28	AK360127	-	non-coding	yes
12_10089	7H	91.12	3.90	-	-	-	-
12_10154	2H	69.05	3.05	AK355324	-	non-coding	yes
12_10159	1H	42.42	2.78	FN555319	pdil4-1	1	no
12_10300	1H	42.42	4.35	EU131177	PR-17c	non-coding	yes
12_10313	0	0	4.12	NM_001061695	Os05g0311000	-	-
12_10392	6H	72.17	3.45	AK361836	-	3	yes
12_10657	7H	61.67	5.82	AK366264	-	3	yes
12_10717	2H	82.98	6.74	-	-	-	-
12_10810	4H	37.88	3.63	AK366265	-	non-coding	yes
12_10824	4H	102.93	2.92	XM_003577507	LOC100843401	non-coding	yes
12_10938	1H	44.59	5.96	AK359548	-	1	no
12_11078	0	0	2.81	AK372406	-	non-coding	yes
12_11151	5H	51.51	5.38	AK354730	-	3	yes
12_11271	1H	136.7	6.00	-	-	-	-
12_11324	2H	72.99	23.02	AK356277	-	non-coding	yes
12_11357	1H	30.15	7.86	AK357047	-	1	no

12_11368	2H	160.46	4.06	AK360506	-	non-coding	yes
12_11468	0	0	3.13	AK359839	-	non-coding	yes
12_20235	2H	61.49	6.27	AK376421	-	non-coding	yes
12_20424	0	0	4.52	AK373140	-	non-coding	yes
12_20649	0	0	3.53	-	-	-	-
12_20760	4H	138.7	5.50	-	-	-	-
12_20793	2H	103.13	3.97	AK371934	-	2	no
12_20981	5H	51.51	4.73	AK370568	-	3	yes
12_21003	0	0	3.04	-	-	-	-
12_21131	1H	59.99	3.29	AK362065	-	3	yes
12_21462	5H	143.29	2.83	AF109194	-	non-coding	yes
12_30005	3H	77.37	2.98	-	-	-	-
12_30060	4H	62.81	10.00	AK365085	-	non-coding	yes
12_30170	3H	92.73	2.95	AK353673	-	3	yes
12_30204	1H	89.77	3.69	AK356336	-	3	yes
12_30226	4H	89.36	8.78	-	-	-	-
12_30242	7H	30.69	3.15	XM_003580160	LOC100839055	1	yes
12_30250	3H	106.67	4.57	AK356601	-	non-coding	yes
12_30275	2H	72.99	3.23	-	-	-	-
12_30342	3H	114.36	3.00	-	-	-	-
12_30389	7H	81.78	3.29	FN179370	AGP-S1a	3	yes
12_30403	1H	128.04	6.73	-	-	-	-
12_30404	1H	42.42	10.32	AK355367	-	3	yes
12_30438	1H	42.42	2.78	AK376647	-	3	yes
12_30475	7H	82.41	23.08	AK372209	-	non-coding	yes
12_30491	2H	49.5	11.08	-	-	-	-
12_30494	5H	171.58	8.49	JX046065	ERS1a	3	yes
12_30504	5H	173.5	4.08	-	-	-	-
12_30528	7H	42.26	9.06	-	-	-	-
12_30574	7H	82.41	4.07	AK370386	-	3	yes
12_30581	7H	79.08	7.89	AK356791	-	3	yes
12_30651	6H	7.87	4.28	AK358128	-	non-coding	yes
12_30674	2H	85.52	2.89	AK369978	-	non-coding	yes
12_30768	5H	42.41	2.81	AK371364	-	non-coding	yes
12_30781	2H	9.12	3.22	AK361785	-	non-coding	yes
12_30976	5H	1.91	3.40	SEG_AY643842S	-	non-coding	yes
12_30988	4H	111.81	3.04	Y14573	Mlo	non-coding	yes
12_30993	4H	49.43	4.61	XM_003577562	LOC100833831	3	yes
12_31032	5H	52.86	8.13	AF326715	adh3	non-coding	yes

12_31035	5H	52.86	9.13	DQ195967	adh3	non-coding	yes
12_31064	5H	51.51	3.07	AK365941	-	3	yes
12_31081	1H	143.2	3.39	AK356376	-	3	yes
12_31183	5H	50.53	3.78	AK362952	-	non-coding	yes
12_31207	0	0	5.27	AK356841	-	3	yes
12_31234	5H	141.88	2.78	AK358248	-	non-coding	yes
12_31270	0	0	3.11	AK361597	-	non-coding	yes
12_31377	1H	128.04	6.56	AK374439	-	3	yes
12_31424	2H	101.72	3.59	AK375713	-	non-coding	yes
12_31486	4H	6.86	9.19	-	-	-	-
