# Supplementary material for: Two Genomic Regions Contribute Disproportionately to Geographic Differentiation in Wild Barley
Source: G3 (Bethesda). 2014 Apr 22;4(7):1193–203. doi: 10.1534/g3.114.010561 (PMC4455769; doi:10.1534/g3.114.010561)
Supplement: Supporting Information [file supp_g3.114.010561_TableS8.pdf]

Table S8 SNPs with SPA score above 95th percentile genome-wide, including genetic position, GenBank ID, gene short name, in non-coding or coding region (1st, 2nd or 3rd positions), and silent or replacement information

SNP Name	Chr	cM	SPA score	GenBank ID	Gene Short Name	Position	Silent
11_10006	1H	76.92	2.83	AM502852	tub4	3	yes
11_10017	2H	81.31	2.96	AK353943	-	3	yes
11_10056	7H	33.49	3.78	AK364330	-	non-coding	yes
11_10104	5H	141.88	2.86	-	-	-	-
11_10169	7H	106.8	3.34	AK367663	-	non-coding	yes
11_10196	2H	89.68	3.14	AK362580	-	3	yes
11_10253	3H	102.66	2.84	-	-	-	-
11_10477	5H	107.19	3.19	AK354978	-	3	yes
11_10518	5H	88.05	2.93	AK372467	-	1	no
11_10536	5H	155.23	3.25	AK354787	-	3	yes
11_10547	7H	160.97	3.48	AK357875	-	3	yes
11_10557	5H	144.6	2.95	AK371672	-	3	yes
11_10580	5H	29.9	3.02	AK372891	-	non-coding	yes
11_10614	4H	111.81	3.80	AK373775	-	3	yes
11_10619	2H	95.58	3.00	AK373516	-	non-coding	yes
11_10653	3H	69.4	3.67	AK356876	-	2	no
11_10668	4H	50.22	3.04	-	-	-	-
11_10685	2H	72.99	4.37	AK362284	-	3	yes
11_10687	7H	139.9	3.32	AK366838	-	3	yes
11_10939	6H	37.29	3.46	AK371919	-	non-coding	yes
11_11012	7H	149.31	2.93	AK360584	-	1	no
11_11111	6H	139.09	2.80	AK366470	-	non-coding	yes
11_11243	7H	121.36	2.79	-	-	-	-
11_11354	2H	68.07	2.76	AK371682	-	non-coding	yes
11_11432	5H	38.78	3.59	-	-	-	-
11_20018	5H	93.66	3.32	AK376008	-	non-coding	yes
11_20029	6H	133.25	3.16	AK354118	-	1	no
11_20086	2H	110.93	2.96	AK358640	-	non-coding	yes
11_20109	4H	29.34	2.97	AK360657	-	3	yes
11_20133	1H	132.16	2.74	AK361091	-	non-coding	yes
11_20145	4H	1.2	2.76	-	-	-	-
11_20260	1H	42.42	2.73	AK355367	-	3	yes
11_20390	2H	72.99	3.47	FN179383	SBE2a	3	yes
11_20485	7H	91.67	2.97	-	-	-	-
11_20620	6H	78.52	2.78	AK366751	-	non-coding	yes
11_21005	2H	54.92	2.76	-	-	-	-

11_21121	5H	58.65	2.93	AK356836	-	3	yes
11_21192	1H	89.77	4.03	AK357712	-	non-coding	yes
11_21244	5H	51.51	4.22	AK359027	-	3	yes
11_21325	5H	123.12	3.06	AK362100	-	non-coding	yes
11_21340	2H	116.5	2.85	-	-	-	-
11_21399	2H	72.99	4.94	AK357878	-	1	no
11_21406	2H	142.67	3.26	AK370573	-	2	no
11_21447	5H	41.45	3.46	AK364513	-	2	no
11_21452	5H	155.23	2.88	AK372156	-	1	no
11_21502	3H	76.43	3.01	AK356987	-	3	yes
12_10014	3H	173.43	3.22	-	-	-	-
12_10089	7H	91.12	2.76	-	-	-	-
12_10154	2H	69.05	4.02	AK355324	-	non-coding	yes
12_10170	4H	88.7	4.17	AK373474	-	non-coding	yes
12_10199	6H	49.67	3.54	AK376992	-	non-coding	yes
12_10203	5H	59.72	3.93	AK356265	-	3	yes
12_10219	0	0	2.91	AY039003	Xantha-f	3	yes
12_10264	5H	47.04	3.42	WHTE1A	E1	non-coding	yes
12_10284	0	0	3.50	XM_003564030	LOC100843138	3	yes
12_10347	4H	43.72	2.81	AK362515	-	non-coding	yes
12_10392	6H	72.17	3.20	AK361836	-	3	yes
12_10581	7H	82.41	3.14	AK375754	-	3	yes
12_10725	5H	52.86	2.95	AK366248	-	non-coding	yes
12_10810	4H	37.88	3.93	AK366265	-	non-coding	yes
12_10824	4H	102.93	3.58	XM_003577507	LOC100843401	non-coding	yes
12_11151	5H	51.51	4.30	AK354730	-	3	yes
12_11271	1H	136.7	2.77	-	-	-	-
12_11288	2H	67.08	3.93	AK366035	-	3	no
12_11310	3H	13.13	3.29	AK372013	-	3	yes
12_11316	2H	73.89	3.35	AK354712	-	non-coding	yes
12_11324	2H	72.99	5.54	AK356277	-	non-coding	yes
12_11408	0	0	2.73	-	-	-	-
12_11444	1H	54.54	2.76	AJ965495	mcb1	non-coding	yes
12_11498	1H	34.45	3.24	-	-	-	-
12_20278	5H	59.72	3.93	AK356265	-	1	no
12_20413	3H	135.43	2.80	AK356460	-	3	yes
12_20649	0	0	2.80	-	-	-	-
12_20685	7H	94.34	3.24	-	-	-	-
12_20981	5H	51.51	4.22	AK370568	-	3	yes

12_21003	0	0	3.57	-	-	-	-
12_21117	4H	0	4.62	AK359776	-	non-coding	yes
12_21131	1H	59.99	2.90	AK362065	-	3	yes
12_21234	7H	68.89	3.77	AK356095	-	2	no
12_21319	7H	82.41	2.92	XM_003573274	LOC100845308	non-coding	yes
12_30046	4H	105.83	2.94	-	-	-	-
12_30068	2H	67.08	3.93	AK364966	-	3	yes
12_30226	4H	89.36	2.89	-	-	-	-
12_30250	3H	106.67	3.75	AK356601	-	non-coding	yes
12_30344	7H	77.02	2.91	AK359310	-	non-coding	yes
12_30404	1H	42.42	2.79	AK355367	-	3	yes
12_30524	5H	116.66	3.39	AK366243	-	non-coding	yes
12_30577	5H	177.9	2.73	-	-	-	-
12_30581	7H	79.08	2.89	AK356791	-	3	yes
12_30637	6H	72.17	4.10	AK368823	-	non-coding	yes
12_30640	3H	108.7	2.78	AK369817	-	non-coding	yes
12_30644	5H	50.53	2.83	AK374038	-	3	yes
12_30724	2H	72.99	2.83	AK368064	-	3	yes
12_30737	3H	59.83	3.50	AK361759	-	3	yes
12_30745	5H	55.44	3.06	AK366468	-	3	yes
12_30834	5H	88.05	3.20	AK372467	-	non-coding	yes
12_30850	5H	98.2	3.28	DQ480160	CBF4B	non-coding	yes
12_30956	6H	142.2	3.20	-	-	-	-
12_30988	4H	111.81	3.36	Y14573	Mlo	non-coding	yes
12_31017	3H	67.86	3.28	AK356796	-	non-coding	yes
12_31021	2H	82.44	2.85	AF112963	Cht2	non-coding	yes
12_31023	5H	4.15	2.80	AK355143	-	non-coding	yes
12_31032	5H	52.86	3.81	AF326715	adh3	non-coding	yes
12_31035	5H	52.86	3.80	DQ195967	adh3	non-coding	yes
12_31043	6H	112.39	2.77	AY349220	Dhn5	3	yes
12_31062	5H	51.51	3.86	AK365941	-	3	yes
12_31064	5H	51.51	4.23	AK365941	-	3	yes
12_31081	1H	143.2	3.60	AK356376	-	3	yes
12_31100	2H	142.67	3.26	AK370573	-	2	no
12_31179	1H	61.39	2.79	AY738115	cbp1	3	yes
12_31189	2H	67.08	2.76	AK364573	-	3	yes
12_31202	0	0	2.73	AK358464	-	non-coding	yes
12_31246	4H	92.41	3.24	AK362249	-	non-coding	yes
12_31411	0	0	2.81	AK372597	-	2	no

12_31486	4H	6.86	3.75	-	-	-	-
12_31511	0	0	3.01	AK360621	-	non-coding	yes
12_31525	3H	134.05	3.32	AK364139	-	2	no
